# Therapeutic potential of the Proprotein Convertase Subtilisin/Kexin family in vascular disease

**DOI:** 10.3389/fphar.2022.988561

**Published:** 2022-09-15

**Authors:** Bianca E. Suur, Melody Chemaly, Moritz Lindquist Liljeqvist, Djordje Djordjevic, Markus Stenemo, Otto Bergman, Eva Karlöf, Mariette Lengquist, Jacob Odeberg, Eva Hurt-Camejo, Per Eriksson, Daniel F.J. Ketelhuth, Joy Roy, Ulf Hedin, Michael Nyberg, Ljubica Matic

**Affiliations:** ^1^ Department of Molecular Medicine and Surgery, Karolinska Institutet, Stockholm, Sweden; ^2^ Global Research Technologies, Novo Nordisk A/S, Maaloev, Denmark; ^3^ Department of Medical Epidemiology and Biostatistics, Karolinska Institutet, Stockholm, Sweden; ^4^ Department of Medicine, Center for Molecular Medicine, Karolinska Institutet, Stockholm, Sweden; ^5^ Science for Life Laboratory, Department of Proteomics, School of Biotechnology, Royal Institute of Technology, Stockholm, Sweden; ^6^ Biopharmaceutical R&D, AstraZeneca, Mölndal, Sweden; ^7^ Department of Cardiovascular and Renal Research, University of Southern Denmark, Odense, Denmark; ^8^ Global Drug Discovery, Novo Nordisk A/S, Maaloev, Denmark

**Keywords:** PCSK, vascular disease, carotid plaque, aortic aneurysm, therapeutic targeting

## Abstract

Proprotein convertase subtilisin/kexins (PCSKs) constitute a family of nine related proteases: PCSK1-7, MBTPS1, and PCSK9. Apart from PCSK9, little is known about PCSKs in cardiovascular disease. Here, we aimed to investigate the expression landscape and druggability potential of the entire PCSK family for CVD. We applied an integrative approach, combining genetic, transcriptomic and proteomic data from three vascular biobanks comprising carotid atherosclerosis, thoracic and abdominal aneurysms, with patient clinical parameters and immunohistochemistry of vascular biopsies. Apart from *PCSK4*, all PCSK family members lie in genetic regions containing variants associated with human cardiovascular traits. Transcriptomic analyses revealed that *FURIN*, *PCSK5, MBTPS1* were downregulated, while *PCSK6/7* were upregulated in plaques vs. control arteries. In abdominal aneurysms, *FURIN, PCSK5*, *PCSK7*, *MBTPS1* were downregulated, while *PCSK6* was enriched in diseased media. In thoracic aneurysms, only *FURIN* was significantly upregulated. Network analyses of the upstream and downstream pathways related to PCSKs were performed on the omics data from vascular biopsies, revealing mechanistic relationships between this protein family and disease. Cell type correlation analyses and immunohistochemistry showed that PCSK transcripts and protein levels parallel each other, except for PCSK9 where transcript was not detected, while protein was abundant in vascular biopsies. Correlations to clinical parameters revealed a positive association between *FURIN* plaque levels and serum LDL, while *PCSK6* was negatively associated with Hb. *PCSK5/6/7* were all positively associated with adverse cardiovascular events. Our results show that *PCSK6* is abundant in plaques and abdominal aneurysms, while *FURIN* upregulation is characteristic for thoracic aneurysms. PCSK9 protein, but not the transcript, was present in vascular lesions, suggesting its accumulation from circulation. Integrating our results lead to the development of a novel ‘molecular’ 5D framework. Here, we conducted the first integrative study of the proprotein convertase family in this context. Our results using this translational pipeline, revealed primarily PCSK6, followed by PCSK5, PCSK7 and FURIN, as proprotein convertases with the highest novel therapeutic potential.

## Introduction

Vascular conditions contribute to the high overall mortality from cardiovascular disease (CVD), dominated by complications of atherosclerosis such as myocardial infarction or stroke, and aneurysm rupture (Wang et al., 2016). Late-stage human atherosclerotic plaques display inflammation, necrotic lipid rich core, calcification, neovessels and intraplaque bleeding, thinning of the fibrous cap, which altogether may lead to rupture and clinical manifestations (Libby et al., 2019). Aneurysms are characterized by smooth muscle cell (SMC) apoptosis and elastin degradation, resulting in a dilatation of the vessel wall and subsequent weakening, followed by rupture of the aorta. While aetiology and pathophysiological mechanisms among vascular diseases can differ, their commonalities are evident on epidemiological level and include similar risk factors, such as smoking, diabetes, male sex, age, plasma lipid levels and hypertension (Fruchart et al., 2004; Kent et al., 2010). Despite the widespread use of lipid lowering and anti-hypertensive medication, CVD mortality has only decreased with approximately 30% since their introduction (Collaboration, 2019). Novel options for lipid-lowering and inflammation-control are scarce and require specific patient characteristics to qualify for the treatment (Ridker et al., 2017; Sabatine et al., 2017). To date, pharmacological interventions used in primary and secondary prevention have shown no benefit, therefore surgery remains the golden standard for both atherosclerosis and aneurysm patients. Thus, there is a need for deeper understanding of molecular pathways capable to render attractive novel therapeutic targets for CVD.

Some of the challenges associated with prioritization of potential therapeutic targets were highlighted in a study from 2014, where the establishment of a link between the target and the disease via genetic causal association coupled to the proof-of-mechanism, was shown to be crucial in early stages of drug development to ensure successful clinical translation (Cook et al., 2014). As an example, recent therapeutic approaches that are clinically tested in atherosclerosis include reducing the residual inflammatory risk by targeting IL-1b, IL-6 or the NLRP3 inflammasome (Ridker et al., 2020), reducing oxidative stress and cellular ageing (Zhang et al., 2020), or decreasing lipoprotein (a) levels (Tsimikas et al., 2015) that unlike HDL-C levels show a genetic link with the disease (Libby et al., 2019). However, there is a paucity of assets in clinical development that directly target vessel wall cell-specific mechanisms, and a very limited number that are at pre-clinical stage. Our rationale is that this approach holds great potential in terms of therapeutic attractiveness, given the novel, specific and likely differentiated mechanisms of action compared to systemic inflammation and/or metabolic dysfunction.

**FIGURE 1:**
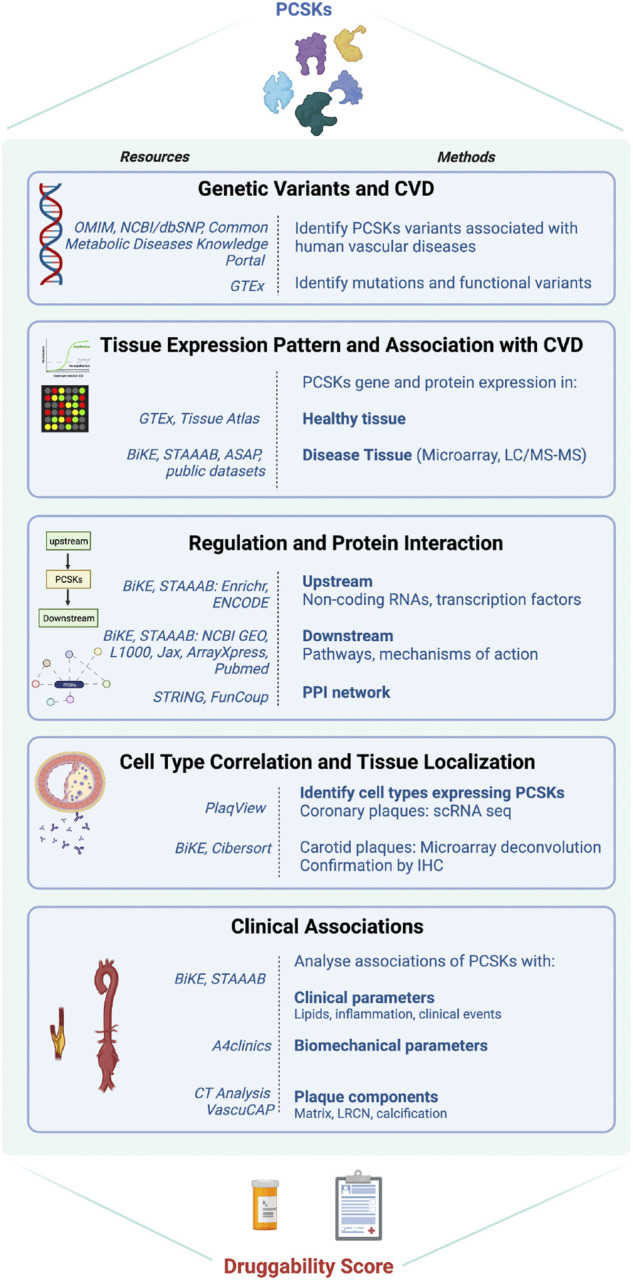
Flowchart of the project. Schematic illustration of the different work-packages, methods, resources used in each package.

We recently discovered Proprotein Convertase Subtilisin/Kexin 6 (PCSK6) as one of the most enriched molecules in human carotid plaque instability, expressed by migrating SMCs upon induction by growth factors and inflammatory cytokines (Perisic et al., 2013). The family of PCSKs emerges as a class of nine proteases with yet unexplored functions in CVD, apart from PCSK9 that is critically involved in regulation of lipoprotein metabolism (Seidah and Prat, 2012). Major recent advances in CVD prevention are linked to PCSK9 inhibitors, which improve removal of LDL-particles from plasma. When it comes to other PCSK members, because of their structural similarities, both redundant and distinct functions have been reported in the cancer field, where they have been shown to activate cytokines, affect cell proliferation/migration and matrix remodeling, which are all key features also in vascular diseases (Seidah and Prat, 2012) (reviewed in Supplementary Table S1). Thus, here we aimed to i) provide a comprehensive landscape of the entire PCSK family in human vascular disease, and ii) evaluate their therapeutic potential for ameliorating CVD by designing a druggability score that could be applied for early “molecular” target assessment, even before the initiation of proof-of-concept and validation studies. Utilising large atherosclerosis and aneurysm biobanks, we explored genetic associations of PCSKs with vascular diseases and their tissue expression pattern. To understand their mechanistic relationships with the disease, layered bioinformatic analyses were done on the omics data from vascular biopsies. To investigate their association with patient parameters, correlation analyses were performed between PCSKs tissue expression levels and various clinical data. Finally, the outcome of all analyses was integrated in a druggability score, summarising the therapeutic potential of each PCSK in vascular disease, with downprioritization of PCSK9 as it is already clinically applied for this purpose (Figure 1).

## Materials and methods

### Data availability statement

All human studies have been approved by the regional Ethical Committees and follow the guidelines of the Declaration of Helsinki. All human samples and data have been collected with informed consent from patients or organ donors’ guardians. The individual human data underlying this article cannot be shared publicly due to the GDPR and ethics laws that regulate the privacy of individuals that participated in the study. The microarray datasets have been made public via the Gene Expression Omnibus (accession numbers GSE21545, GSE125771, GSE26155). Other group level data will be shared pertaining a reasonable request to the corresponding author.

### Human biobanks

Carotid atherosclerosis: Patients undergoing surgery for high-grade (>50% NASCET (North American Symptomatic Carotid Endarterectomy Trial) (Naylor et al., 2003) carotid stenosis at the Department of Vascular Surgery, Karolinska University Hospital, Stockholm, Sweden were consecutively enrolled in the study and clinical data recorded on admission. Symptoms (S) were defined as transitory ischemic attack (TIA), minor stroke (MS) and *amaurosis fugax* (retinal TIA). Patients without qualifying symptoms within 6 months prior to surgery were categorised as asymptomatic (AS) and indication for carotid endarterectomy (CEA) based on results from the Asymptomatic Carotid Surgery Trial (ACST) (Halliday et al., 2010). Carotid endarterectomies (carotid plaques, CP) were collected at surgery and retained within the Biobank of Karolinska Endarterectomies (BiKE). The study cohort demographics, details of sample collection and processing, transcriptomic analyses by microarrays and proteomic analyses were as previously described in detail (Folkersen et al., 2012; Perisic et al., 2016). Briefly, plaques were divided transversally at the most stenotic part; the proximal half of the lesion used for RNA preparation while the distal half was processed for histology. The microarray dataset is available from Gene Expression Omnibus (GSE21545). This dataset contains n = 127 carotid plaques (87 from S and 40 from AS patients) and n = 10 normal arteries (9 iliac and 1 aorta). A second independent microarray dataset was used for correlating gene expression to plaque phenotype parameters assessed by diagnostic computed tomography (CT, n = 40) (Sheahan et al., 2018; Karlöf et al., 2019). This dataset is available from Gene Expression Omnibus (GSE125771). For proteomic analysis CEA tissue specimens from 18 patients (n = 9 S, n = 9 AS) matched for sex (male), age, and statin medication were analysed using LC-MS/MS as previously described (Branca et al., 2014; Perisic Matic et al., 2016). Human studies from BiKE are approved by the Ethical Committee of North Stockholm and follow the guidelines of the Declaration of Helsinki. All human samples and data in BiKE are collected with informed consent from patients or organ donors’ guardians. Tissue and blood sampling are conducted as part of the ordinary medical and surgical procedures and does not put the patients at unnecessary risk.

Abdominal aortic aneurysm: Samples of the anterior vessel wall of AAA were obtained from n = 76 patients with AAA undergoing elective open surgery and control samples were procured from the abdominal aorta of n = 13 solid organ transplant donors. The samples were immediately immersed in RNA stabilization solution (RNAlater, Thermo Fisher Scientific, Waltham, MA), separated into (intima)/medial, referred to as media, and adventitial wall layers, and then stored in −80°C within the framework of the Stockholm AAA Biobank (StAAAB). RNA was subsequently isolated by Qiazol Lysis Reagent (Qiagen) and purified using the miRNeasy or RNeasy minikits (Qiagen). The concentration of RNA was measured with Nanodrop ND 1000 (Thermo Fisher Scientific). Gene expression was analyzed with Affymetrix HTA 2.0 Genechip arrays (Thermo Fisher Scientific). Raw CEL intensity files were normalized with Guanine Cytosine Count Normalization and Signal Space Transformation using the Transcriptome Analysis Console software (Thermo Fisher Scientific). All samples passed quality control in the same software. Preoperative computed tomography angiography (CTA) of high quality was available for n = 64 of the included patients with AAA. Patients with AAA gave informed consent and organ donors consented to tissue being used for research at the time of enrollment to the Swedish national organ donor register. The procurement, biobanking and study of these samples was approved by the regional Ethics Review Board in Stockholm.

Thoracic aortic aneurysm: The **A**dvanced **S**tudy of **A**ortic **P**athology (**ASAP**) is a prospective, single-centre, observational cohort study of patients with aortic valve and ascending aortic disease undergoing elective open-heart surgery at the Cardiothoracic Surgery Unit, Karolinska University Hospital in Stockholm, Sweden. Inclusion criteria are applied on patients aged 18 or above with aortic valve disease (i.e., aortic stenosis or regurgitation) and/or ascending aorta dilatation (aneurysm or ectasia of the ascending aorta including the aortic root) but devoid of coronary artery disease (defined as lacking significant stenosis on coronary angiogram) and primarily not planned for another concomitant valve surgery (Folkersen et al., 2010; Folkersen et al., 2011). Only samples from patients with a tricuspid aortic valve (n = 77) were analysed for this study. The microarray dataset is available from Gene Expression Omnibus (GSE26155). All human samples were collected with informed consent from patients; studies were approved by the regional Ethical Committee and follow the guidelines of the Declaration of Helsinki.

### Immunohistochemical analysis

Antibodies: Primary antibodies anti-PCSK5 (HPA031072, 1/10) and anti-PCSK6 (HPA004774, 1/25) were obtained from the Human Protein Atlas (HPA). Commercially purchased rabbit anti-human antibodies used in this study were anti-FURIN (sc-133142, B-6, Santa Cruz Biotechnology, 1/25), anti-PCSK7 (12044-1-AP, Proteintech, 1/20), anti-PCSK9 (PA5-14218, Invitrogen, 1/50). The following mouse anti-human antibodies were used, anti-CD3 (CM110A, Biocare Medical, 1/100), anti-CD163 (CM353A, Biocare Medical, 1/50), anti-SMA (M0851, DAKO, 1/1500) and anti-vWF (M0616, DAKO, 1/400).

Immunohistochemistry (IHC) staining*:* All IHC reagents were purchased from Biocare Medical (Concord, CA, United States). Tissues were fixed in 4% Zn-formaldehyde for 48 h, placed in 70% ethanol, dehydrated in a LOGOS microwave hybrid tissue processor (Milestone Medical, Sorisole, Italy) and embedded in paraffin blocks. Briefly, 5 μm sections were deparaffinized in Histolab Clear and rehydrated in gradually increasing ethanol dilutions. For antigen retrieval, slides were subjected to high-pressure boiling in DIVA buffer (pH 6.0). After blocking with Background Sniper, primary antibodies diluted in Da Vinci Green or Renoir Red solution were applied on slides and incubated at room temperature for 1 h. Isotype rabbit and mouse IgG were used as negative controls. A double-stain probe-polymer detection kit (Mach 2) containing both alkaline phosphatase and horseradish peroxidase was applied, with subsequent detection using Warp Red and Vina Green. All slides were counterstained with Hematoxylin QS (Vector Laboratories), dehydrated and mounted in Pertex (Histolab, Gothenburg, Sweden). Images were taken using a Nikon Eclipse E800 microscope, using NIS-elements software.

### Statistical, bioinformatic and modelling analyses

Genetic variant and CVD trait association data was downloaded from the Common Metabolic Diseases Knowledge Portal https://hugeamp.org/ on 16 April 2021, including associations for all variants within 50 kb of the gene body and gene level associations computed by MAGMA (de Leeuw et al., 2015). Significant single tissue eQTL data for each gene was downloaded from the GTEx Portal https://gtexportal.org/ on 16 April 2021. A set of CVD trait categories (anthropometric, atrial fibrillation, cardiovascular, glycemic, hematological, hepatic, lipids, renal, stroke as described on https://hugeamp.org/) and tissues (adipose – subcutaneous and visceral, aorta, tibial artery, heart, whole blood, liver, pancreas and spleen) were defined and used to filter the eQTL and genetic association data before analysis. Integration and analysis of genetic association and eQTL data was performed in R (2020) using custom scripts. In cases of redundant or duplicate associations between variants, genes, tissues and phenotypes, the lowest *p*-value was considered representative for the sake of visualization.

Transcriptomic dataset analyses were performed with GraphPad Prism 6 software using a two-sided Student’s t-test assuming non-equal deviation, with correction for multiple comparisons according to Bonferroni, as previously described (Perisic et al., 2016; Perisic Matic et al., 2016). Pearson or Spearman correlations were calculated to determine the association between mRNA expression levels from microarrays, as appropriate. FunCoup software was used to identify connections between the members of the PCSK family across healthy tissues based on published literature (http://funcoup.sbc.su.se) (Ogris et al., 2017). Cell compartments localisation data was analysed using the SubCellBarCode software (http://subcellbarcode.org) (Orre et al., 2019) and based on the genes identified in the FunCoup connections within the PCSK family. Gene set enrichment analyses on Gene Ontology (GO) terms were performed using GOrilla (http://cbl-gorilla.cs.technion.ac.il) and ENRICHR (http://amp.pharm.mssm.edu/Enrichr) software. Filtering of overlapping GO categories was performed using Revigo software (http://revigo.irb.hr). Overall Pearson correlation analyses between PCSKs and global mRNA expression levels from microarrays were conducted using Morpheus software (http://software.broadinstitute.org/morpheus). Transcription factors binding motif analysis was performed on the ranked lists of genes from global correlations using MotifMap (http://motifmap.ics.uci.edu) and ENRICHR (http://amp.pharm.mssm.edu/Enrichr) software (JASPAR and TRANSFAC). Protein-protein interactions were also predicted from the ranked lists of genes from global correlations using ENRICHR (http://amp.pharm.mssm.edu/Enrichr) software, considering FDR<0.05 as the cutoff value. In all analyses adjusted *p* < 0.05 was considered to indicate statistical significance.

A single cells RNA (scRNA) sequencing dataset from human coronary artery atherosclerotic plaques (*Wirka et al. 2019*) made available by Ma et al. (2021) via the PlaqView website (https://millerlab.shinyapps.io/PlaqView/), was used to explore expression levels of FURIN, PCSK5, PCSK6 and PCSK7 and generate t-SNE plots. To enumerate the relative abundance of cellular populations in plaque composition, we applied the deconvolution strategy to BiKE microarrays using the Cibersort web software (https://cibersort.stanford.edu). Cell populations were defined using pre-assigned cell-type signature markers and used to estimate the relative frequencies deconvolved from microarray data. We used two publicly available signature markers files, one generated from scRNA sequencing of atherosclerotic human coronary arteries resolving the 15 major cell types (Wirka et al., 2019), and the other defining 22 immune cell populations (Newman et al., 2015).The output fractions obtained from deconvolution were used to perform a correlation analysis between the different cell types and PCSKs and create heatmaps using GraphPad Prism 6 software.

Carotid plaques were assessed in pre-operative computed tomography angiographies (CTAs) using a semi-automated, histology-validated software as previously described (vascuCAP, Elucid Bioimaging, Wenham, Mass, United States) (Sheahan et al., 2018) rendering tissue and structural characteristics of the plaque, e.g. calcification (CALC), lipid-rich necrotic core (LRNC) and the remaining tissue named matrix (MATX) classified as neither LRNC or CALC; in their absolute and proportional volume (Karlöf et al., 2019; Karlof et al., 2021).

The druggability score was estimated based on compiled data from each section of the study workflow (Figure 1). Each PCSK received a score between 0 and 3, where 0 reflects no association and 3 represents a strong association with the corresponding section. For example, for the section related to genetic variants associated with CVD (workflow section 1), a score of 0 refers to no described associations between SNPs in a certain PCSK and CVD or CVD risk factors, whereas a score of 3 represents strong associations. For the section corresponding to the tissue expression pattern of PCSKs and their association with CVD, a PCSK received a score of 3 if it presented a restricted tissue expression pattern in CVD related tissues and if it demonstrated high expression in carotid plaques and specifically in symptomatic patients in BiKE. PCSKs received a lower score if they presented a ubiquitous tissue expression pattern and if they were not highly expressed in carotid plaques in BiKE. A detailed description of the druggability score estimation is provided in (Supplementary Table S2). The study workflow and druggability score figures were created with www.BioRender.com.

## Results

### Genetic variants in PCSK family members associate with CVD related human traits

**FIGURE 2:**
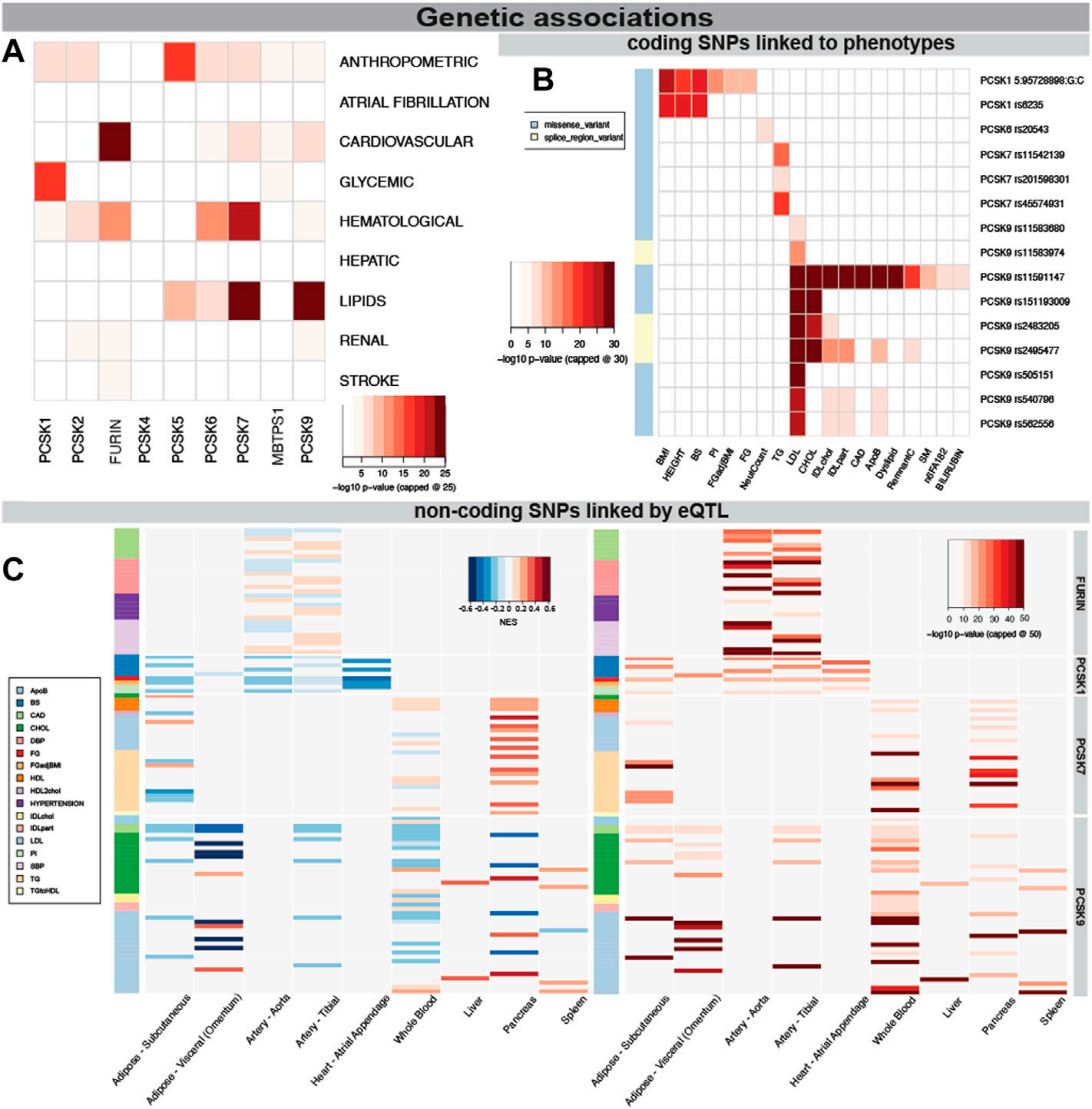
Genetic associations of PCSK family members with cardiovascular traits. PCSK gene variants analysed by MAGMA software for top CVD associated categories. The lowest *p*-value per disease category is shown for each member of the PCSK family, -log10 *p*-value was capped at 25 for clarity **(A)**. Associations of coding SNPs in the PCSK family linked to various cardiovascular phenotypes. -log10 *p*-value was capped at 30 for clarity **(B)**. Non-coding PCSK SNPs linked via eQTL analyses to various CVD related tissues. Heatmap on the left shows the GTEX eQTL Normalised Effect Size (NES) to indicate direction of the expression change. The heatmap on the right shows the GWAS *p*-value for each non-coding SNP. -log10 *p*-value was capped at 50 for clarity **(C)**. Additional individual SNP information may be found in Supplementary Table S2. Single nucleotide polymorphism (SNP), expression quantitative trait loci (eQTL), genome wide association study (GWAS).

Because of the power of genetic associations to disease for selection of successful therapeutic targets, we first searched for genetic links between PCSK family members and CVD related human phenotypes. We found that SNPs in *PCSK1* genomic region were mainly associated with glycemic phenotypes, while in *PCSK2* with hematological and anthropometric phenotypes. Variants in *FURIN*, *PCSK6* and *PCSK7* were also associated with hematological phenotypes, and SNPs in *PCSK5-7* and *PCSK9* genes associated mainly with lipid levels (Figure 2A). Several coding SNPs were linked to specific phenotypic manifestations, i.e. missense variants in *PCSK1* associated with BMI and height. A SNP in the *PCSK6* gene was associated with neutrophil count. SNPs in *PCSK7* associated specifically with triglycerides (TG), while SNPs in PCSK9 strongly associated with LDL, cholesterol levels, and coronary artery disease (CAD) (Figure 2B, Supplementary Table S3).

We also investigated whether disease linked variations surrounding the different PCSKs were directly linked to gene expression levels in relevant cardiovascular organs through expression quantitative trait loci (eQTL) analysis. By integrating the eQTL Normalised Effect Size (NES) and GWAS beta values for the same variants, we found that SNPs associated with increased risk of CAD and hypertension were associated with higher *FURIN* expression in the arteries, suggesting that FURIN is detrimental for these phenotypes. In addition, we observed SNPs linked with increased fasting glucose and decreased proinsulin levels were associated with lower *PCSK1* expression in the adipose tissue, arteries and in the heart. SNPs surrounding the *PCSK7* gene were found to be associated with its higher expression in pancreas and also with increased TG levels. Non-coding SNPs surrounding the *PCSK9* gene associated mainly with increases in cholesterol and LDL, similar to the coding SNPs. Through eQTL they were associated with decreased expression of this gene in nearly all investigated tissues, with the exception of liver (Figure 2C, Supplementary Figure S1, Supplementary Table S4).

### *PCSK6* is the most enriched proprotein convertase in atherosclerotic plaques and abdominal aneurysms, while *FURIN* is upregulated in thoracic aneurysms

We next accessed the mRNA expression pattern of all nine PCSK family members across various normal tissues using public RNA sequencing (RNAseq) data. Whereas *MBTPS1* showed ubiquitously wide expression, *PCSK1* was undetectable in most tissues, *PCSK2* was only detected in thyroid gland and *PCSK4* in testis. *PCSK5* and *PCSK7* were expressed mostly in normal cardiovascular tissues. *PCSK6* and *PCSK9* showed a similar restricted pattern, being detected at high levels in liver and cerebrospinal system and *PCSK6* also in the spleen (Supplementary Figure S2).

**FIGURE 3:**
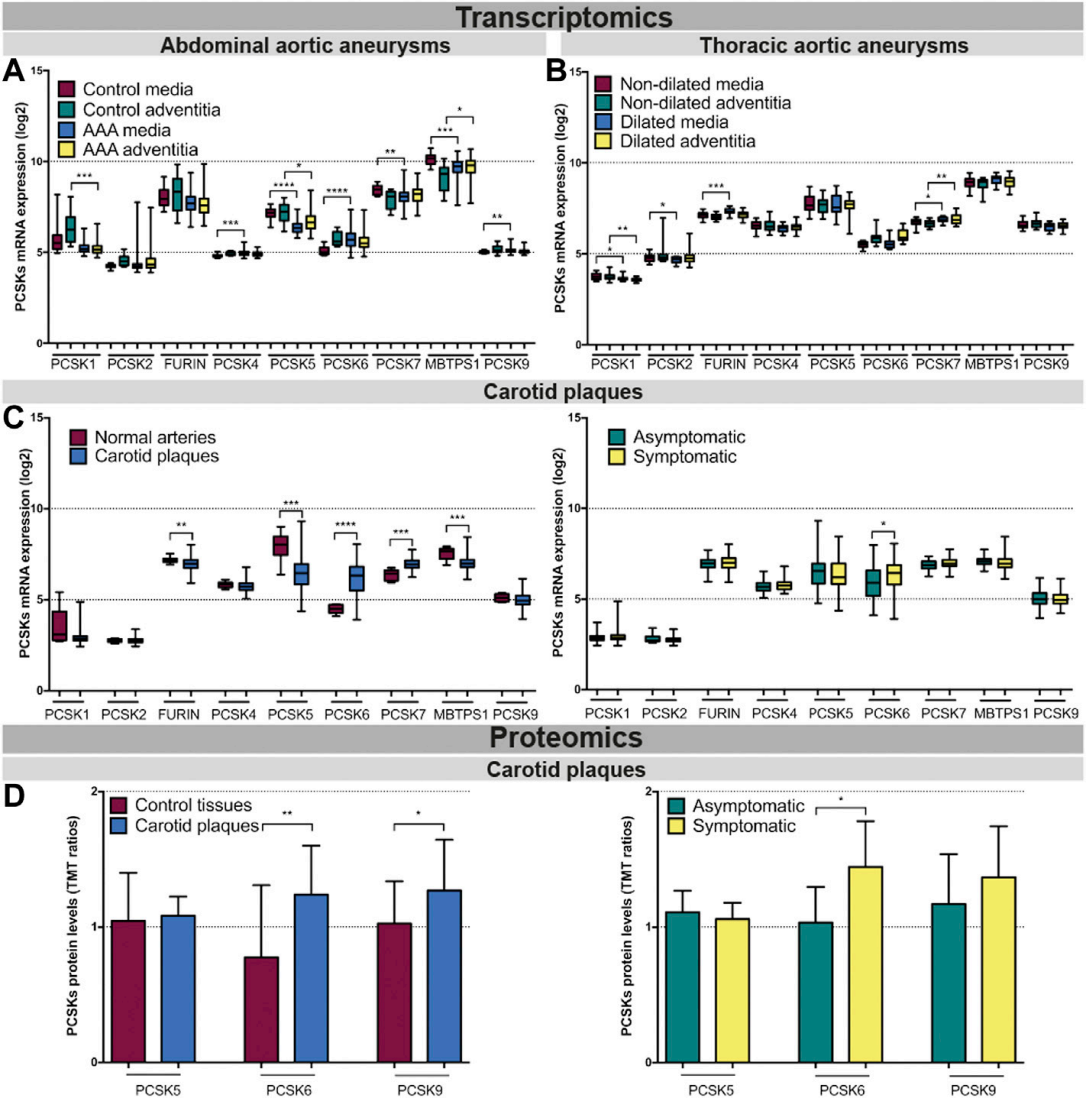
Transcriptomic and proteomic analyses of the PCSK family in human vascular tissues. PCSK mRNA expression in abdominal aortic aneurysm tissues comparing AAA media (n = 76) with control media (n = 13) and AAA adventitia (n = 76) with control adventitia (n = 13) **(A)**. PCSKs mRNA expression in thoracic aortic aneurysm tissues from TAV patients comparing dilated media (n = 22) with non-dilated media (n = 24) and dilated adventitia (n = 22) with non-dilated adventitia (n = 19) **(B)**. mRNA levels of the different members of the PCSK family in atherosclerotic plaque tissues comparing carotid plaques (n = 127) with normal arteries (n = 10) and plaques from symptomatic patients (n = 87) with those from asymptomatic ones (n = 40) **(C)**. Proteomic analysis of the PCSK family members in atherosclerosis comparing plaques vs. matched adjacent arterial control tissues as well as plaques from symptomatic vs. asymptomatic patients **(D)**. Data is shown as mean with SD, *p*-values were calculated using unpaired *t*-test with Welch’s correction. *p < 0.05, **p < 0.01, ***p < 0.001, ****p < 0.0001. Abdominal aortic aneurysm (AAA), tricuspid aortic valve (TAV).

PCSK transcriptomic levels were then investigated in microarrays from several independent human biobanks of vascular diseases. We showed that *PCSK1*, *PCSK2*, *PCSK4* and *PCSK9* were expressed at low to moderate levels in most of the disease tissues examined, and without any major changes between diseased and control tissues. In AAA, we observed that *PCSK5*, *PCSK7* and *MBTPS1* mRNA levels were generally downregulated in diseased compared to control tissues, while only *MBTPS1* was increased in AAA adventitia vs. controls. Here, *PCSK6* was the only proprotein convertase upregulated in the AAA media compared to controls (Figure 3A). In TAA tissues, *FURIN* and *PCSK7* transcripts were increased particularly in dilated media compared to non-dilated, and *PCSK7* was also increased in dilated adventitia (Figure 3B). In atherosclerosis, we found that the expression of *FURIN*, *PCSK5* and *MBTPS1* was significantly lower in carotid plaques vs. normal artery controls, while *PCSK6* and *PCSK7* were increased (Figure 3C, left panel). Moreover, *PCSK6* was the only proprotein convertase which remained significantly upregulated when plaques were stratified according to patient symptoms (Figure 3C, right panel). This could be extended also by LC/MS-MS for a few PCSKs that were detected, where PCSK6 protein was consistently upregulated in both plaques vs. control tissue comparison and in plaques from symptomatic patients (Figure 3D). Interestingly, proteomic data also revealed increased levels of PCSK9 in plaques.

### FURIN, PCSK5, PCSK6 and PCSK7 associate with key pathways in vascular disease

**FIGURE 4:**
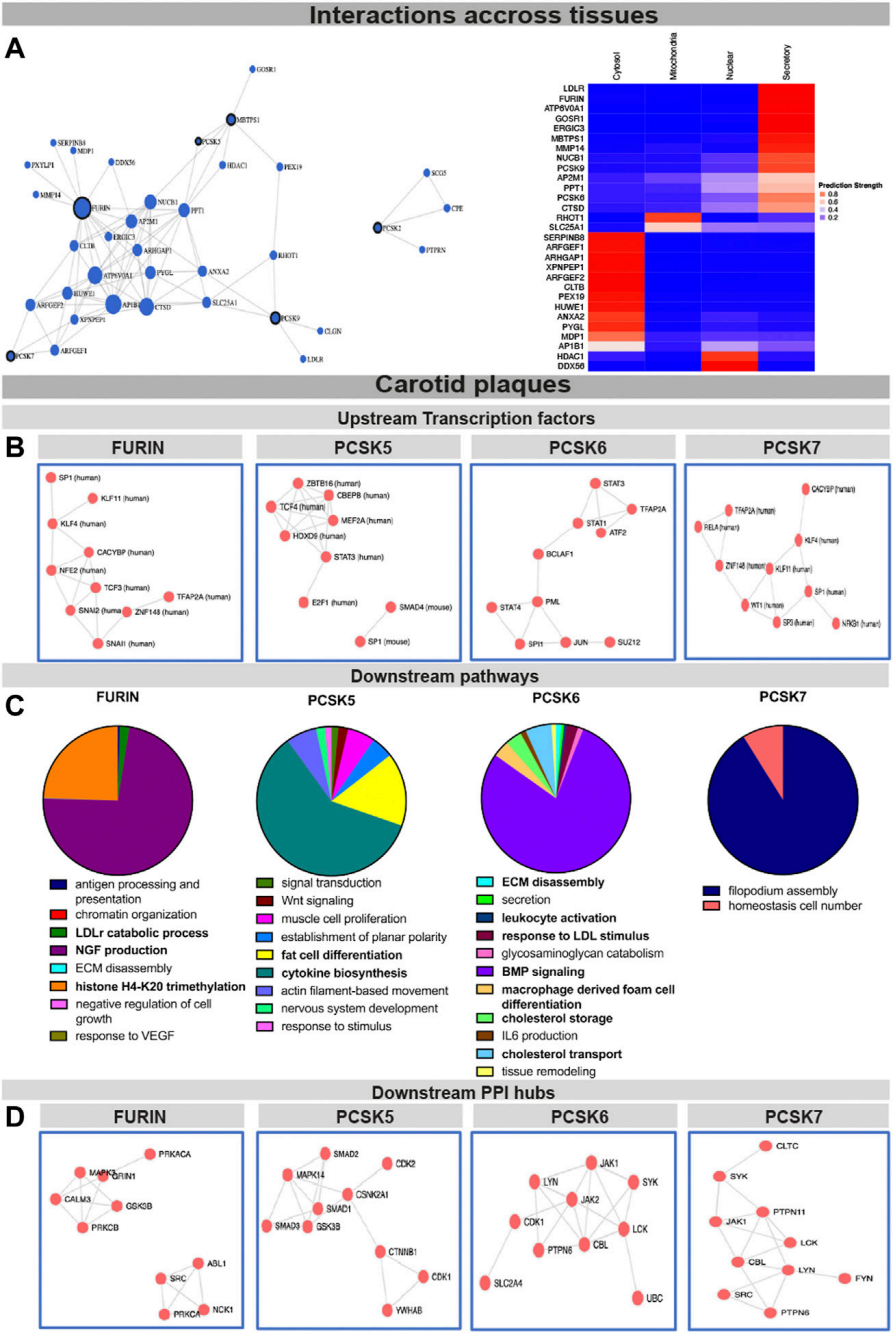
Interconnectivity and expression correlations within the PCSK family in healthy tissues and carotid plaques. Interconnectivity analysis among the nine members of the PCSK family, based on the FunCoup software. Size of the nodes corresponds to the enrichment of functional connections with other proteins in the network. Subcellular localisation of the proteins from the network was determined and plotted using SubCellBarCode **(A)**. Transcription factors predicted to regulate the genes positively correlated with FURIN and PCSK5-7 in carotid plaques **(B)** Gene set enrichment analysis of genes positively correlated with FURIN and PCSK5-7 in carotid plaques **(C)**. Predicted PPI hubs of genes positively correlated with FURIN and PCSK5-7 in carotid plaques **(D)**. Genes used for these analyses show Pearson correlation r > 0.5 to each selected PCSK. Protein-protein interaction (PPI).

To begin assessing the molecular mechanisms as well as interconnectivity related to the PCSK family, we constructed a network based on their couplings across all publicly available data, defined as co-interactions, co-expression, or functional links. We found that FURIN, PCSK5, PCSK7, MBTPS1 and PCSK9 form a functional network with each other through direct (i.e. PCSK5-MBTPS1) or indirect protein-protein interactions (Figure 4A, left panel, Supplementary Table S5), with FURIN and MBTPS1 being the largest nodes in this network. Predictions of cellular localization suggested that the majority of proteins in this network are part of the secretory system or found within the cytosol (Figure 4A, right panel). Based on the combination of genetic, expression and functional coupling data, we concentrated further in-depth analyses on fewer selected PCSKs with highest potential for targeting in CVD: *FURIN*, *PCSK5*, *PCSK6* and *PCSK7*.

To gather insight about the possible upstream regulators of these PCSKs and their functionally related genes, transcription factors were predicted based on all transcripts positively correlated with *FURIN* and *PCSK5-7* expression in carotid plaques. We found that the *FURIN* correlated genes were largely regulated by transcription factors KLF4, KLF11 and TCF3, while *PCSK5* correlated genes were associated with transcription factors TCF4 and STAT3. Transcription factors linked with *PCSK6* correlated network included STAT1, STAT2, STAT4 and BCLAF1, while *PCSK7* network similarly as *FURIN* was associated with KLF4 and KLF11. These PCSK networks were also associated with one common transcription factor SP1 (Figure 4B).

To begin unravelling the possible mechanistic roles of each PCSK in plaques, we further performed GSEA of the global microarray correlations. In plaques, *FURIN* expression was strongly correlated with NGF production, LDLr catabolism and histone H4K20 signatures. *PCSK5* correlated mainly with cytokine biosynthesis and fat cell transdifferentiation. With respect to *PCSK6*, we found that BMP signaling, lipid metabolism, as well as secretion, ECM degradation and leukocyte activation were the major pathways correlating to its expression. The major pathway associated with *PCSK7* in carotid plaques was filopodium assembly (Figure 4C). These globally correlated genes for each PCSK were also used to form predicted protein-protein interaction (PPI) networks. Here, *FURIN* expression correlated with PPI networks containing MAPK3, CALM3 and GRIN1 as well as a separate network containing ABL1, NCK1 and SRC. *PCSK5* expression correlated to a PPI network containing several members of the SMAD family (SMAD1-3) as well as CDK1-2. *PCSK6* expression correlated to a PPI network containing JAK1-2, CDK1 and PTPN5, and *PCSK7* with a network containing JAK1, PTPN6 and PTPN11 (Figure 4D).

### Single-cell analyses indicate cell types that express PCSKs in vascular disease

**FIGURE 5:**
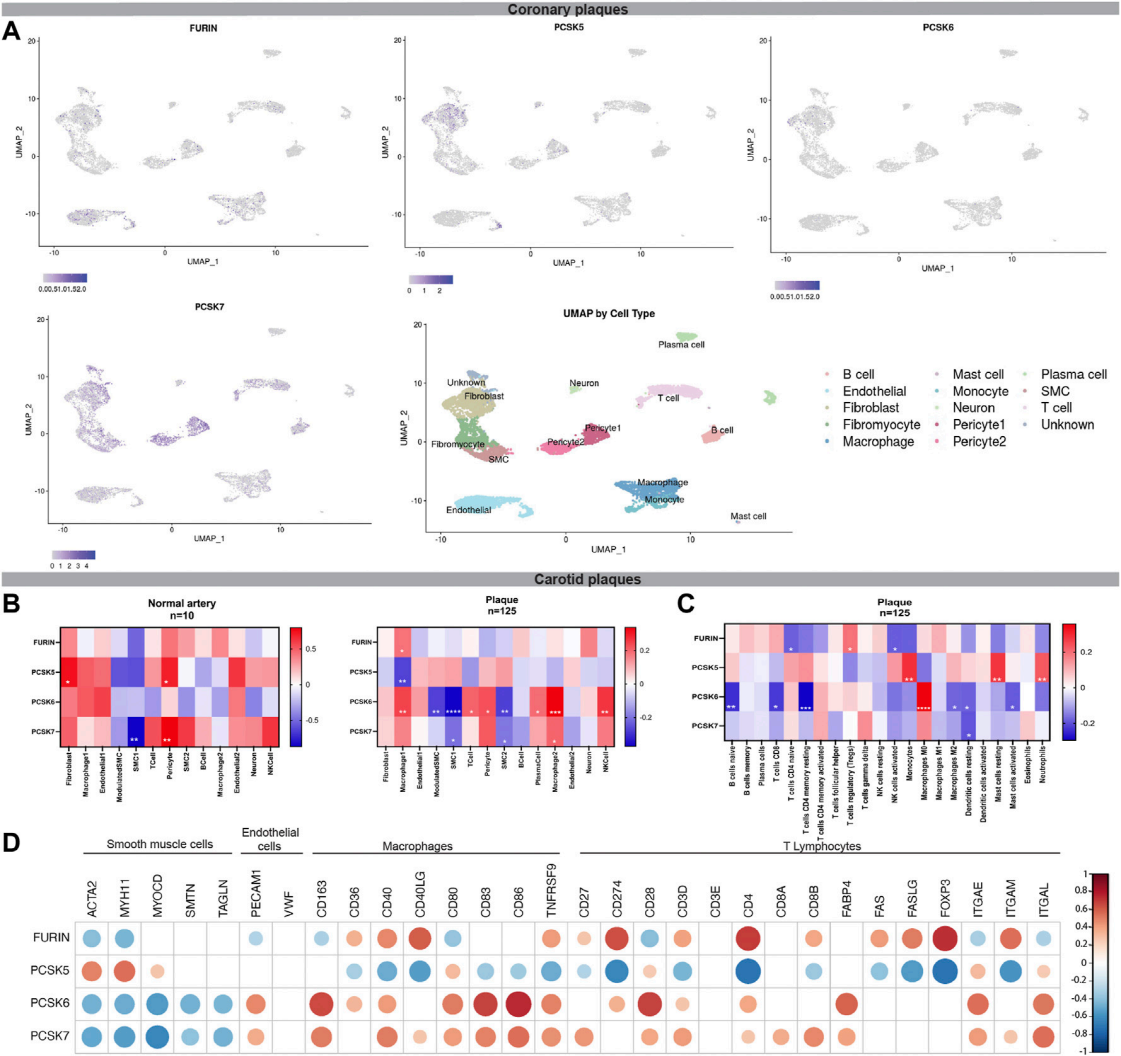
Single cell analysis of FURIN and PCSK5-7 expression in atherosclerotic lesions. Public single-cell RNA sequencing data interrogated using Plaqview for the expression of FURIN and PCSK5-7. Data is visualised in t-SNE plots showing cell types that express the respective PCSK in coronary plaques **(A)**. Heatmaps representing correlation of FURIN and PCSK5-7 expression to major cell type fractions deconvolved from carotid plaque microarray data based on single cell RNA sequencing data from coronary plaques. Squares with stars represent statistically significant correlations **(B)**. Heatmap representing correlation of FURIN and PCSK5-7 expression specifically with inflammatory cell type fractions in carotid plaques **(C)**. Correlations of FURIN and PCSK5-7 transcripts to the expression of individual typical cell markers in carotid plaques **(D)**. t-distributed stochastic neighbour embedding (t-SNE).

While these molecular analyses provide an insight into the possible functions for PCSKs in vascular disease, the majority of these pathways or mechanisms are not cell-type specific. To gain clues into which cells express the selected PCSKs in vascular biopsies, we investigated their expression in publicly available scRNA sequencing data of coronary plaques (n = 5). Here, *FURIN* appeared to be broadly expressed by fibroblasts, SMCs, monocytes and macrophages, T cells, endothelial cells and pericytes. *PCSK5* expression seemed more confined to the fibroblast cluster, with some detection in endothelial cells, SMCs, T cells, monocytes, macrophages and pericytes. *PCSK6* expression was mainly confined to the fibroblast cluster with some expression in the T cells, monocyte, macrophage and endothelial cells. *PCSK7* was quite abundant in most cell types found in coronary plaques (Figure 5A).

To increase the cohort for cell-specific analyses, deconvolution was also performed on BiKE bulk microarray data using this scRNAseq data, as well as specific immune cell signatures, thereby estimating the relative cell fractions from a larger number of both normal arteries (n = 10) and plaques (n = 127). In normal arteries, *FURIN* and *PCSK6* expression showed no significant correlations to any of the deconvoluted cell type fractions. *PCSK5* expression correlated positively to fibroblasts and pericytes, while *PCSK7* expression correlated negatively to the contractile SMC1 fraction and positively to pericytes (Figure 5B, left panel). In carotid plaques, *FURIN* expression was positively correlated to the macrophage 1 fraction, while *PCSK5* correlated negatively with these cells. *PCSK6* correlated positively to both macrophage 1 and 2 fractions, T cells, pericytes, plasma cells and NK cells, but negatively with contractile (SMC1) and modified SMC (SMC2) fractions. *PCSK7* expression was positively correlated with the macrophage two fraction and negatively with contractile and modified SMCs (Figure 5B, right panel). Focusing only on inflammatory cells, we found that *FURIN* was positively correlated to the regulatory T cells, but negatively to naïve CD4 T cells and activated NK cells. *PCSK5* correlated positively with monocytes, resting mast cells and neutrophil fractions. *PCSK6* correlated positively to M0 macrophages and negatively to naïve B cells, CD8 T cells, resting memory CD4 T cells, M2 macrophages, resting dendritic cells and activated mast cells. *PCSK7* correlated negatively to resting dendritic cells (Figure 5C).

To validate these scRNAseq findings, expression of selected PCSKs was also correlated directly to various markers classically expressed by SMCs, endothelial cells, macrophages and T lymphocytes, in BiKE plaque bulk microarray data. Here we confirmed that *FURIN* negatively correlated to classical markers of SMCs and endothelial cells, while correlations with macrophages and T lymphocytes were mostly positive. *PCSK5* mRNA expression correlated positively with SMCs and endothelial cell markers, while negative correlations were observed with macrophage and T lymphocyte markers. Overall strong correlations were found for *PCSK6* in plaques, negative with SMC markers and positive with endothelial cells, macrophages and T lymphocytes. *PCSK7* correlated negatively with SMC markers, while correlations with endothelial cells, macrophages and T lymphocytes were systematically positive (Figure 5D).

### Immunohistochemistry localizes PCSKs to major cell types in vascular lesions

**FIGURE 6:**
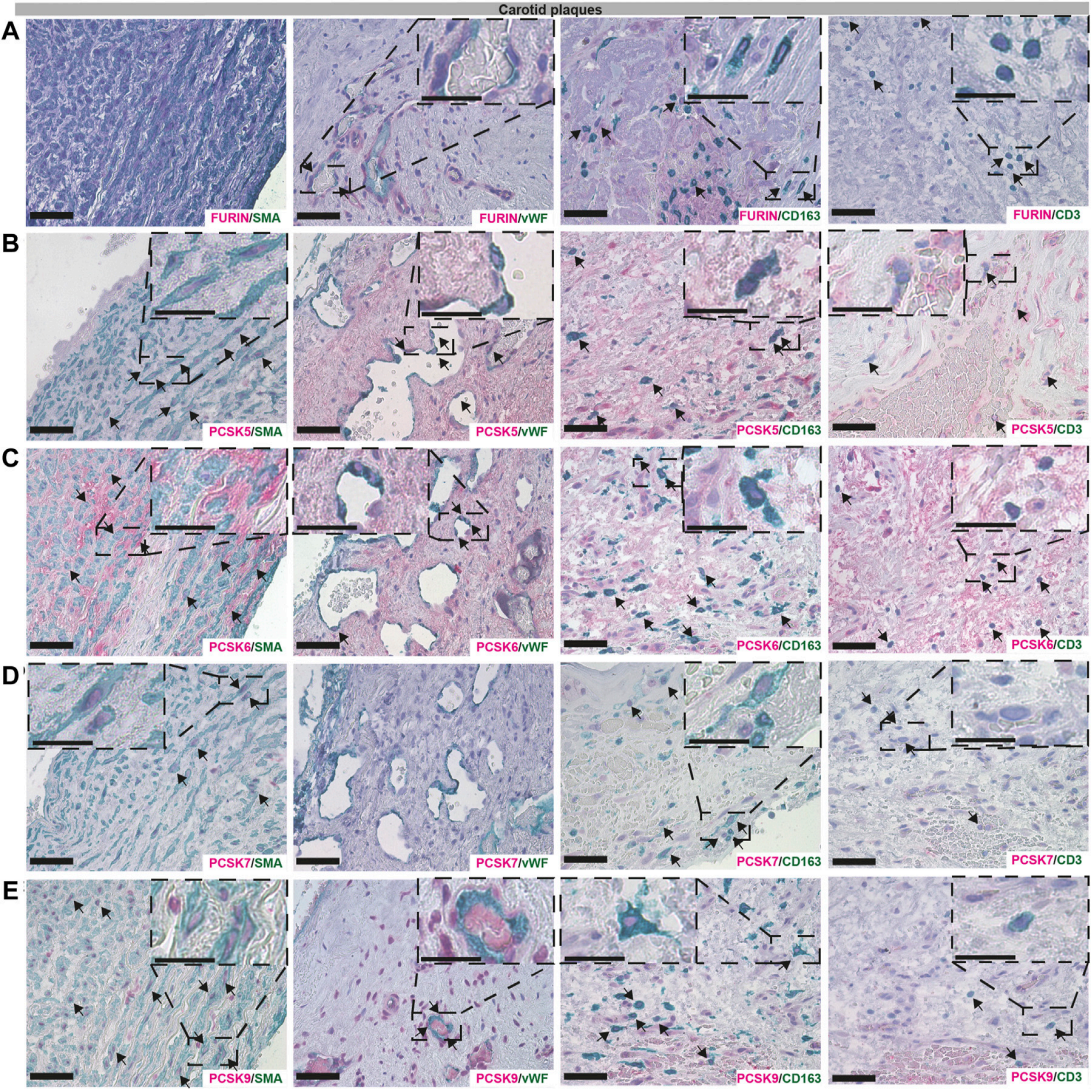
Co-localisation of FURIN, PCSK5-7 and PCSK9 with major cell types in carotid plaques. Immunohistochemistry co-stainings of classical markers CD3 (T cells), CD163 (macrophages), SMA (smooth muscle cells) and vWF (endothelial cells) with FURIN **(A)**, PCSK5 **(B)**, PCSK6 **(C)**, PCSK7 **(D)** and PCSK9 **(E)**. Scale bar represents 50 μm and in the enlarged insets 25 μm.

Next, we sought to validate the bioinformatic data and localize PCSK proteins in vascular biopsies *in situ*, by single and double immunohistochemistry with classical markers of SMCs (α− smooth muscle actin, SMA), endothelial cells (Von Willebrand factor, vWF), macrophages (CD163) and T lymphocytes (CD3). Overall, we observed similar co-localization patterns for PCSKs in plaques (Figure 6, Supplementary Figure S3) and in AAA tissues (Supplementary Figure S4). Staining for FURIN co-localized with vWF, CD163 and CD3 but not with SMA signal (Figure 6A). PCSK5 protein was found to be both extra- and intra-cellular, weakly expressed by SMA+ cells and in areas rich with CD163+ and CD3^+^ cells in plaques, as well as in the matrix around the neovessels. No co-localization was observed for PCSK5 and vWF (Figure 6B). PCSK6 protein was found to be mostly extracellular and co-localization was detected mainly with SMA + cells. As expected based on expression correlations, PCSK6 signal was also detected in vWF+, CD163+ and CD3^+^ areas (Figure 6C). For PCSK7, weak co-localization with SMA+ and CD163+ cells was observed on protein level, but not with vWF+ and CD3^+^ cells (Figure 6D).

Although PCSK9 transcript expression levels were confirmed to be low in all vascular tissues in our study, the protein was detected at high levels as indicated by LC-MS/MS, and mostly intracellular by immunohistochemistry. We found that PCSK9 staining co-localized with SMA+, CD163+ and CD3^+^ cells. In particular, PCSK9 protein was found abundantly in cells surrounding the vWF+ neovessels (Figure 6E).

### PCSK expression in tissues correlates with clinical vascular risk parameters

**FIGURE 7:**
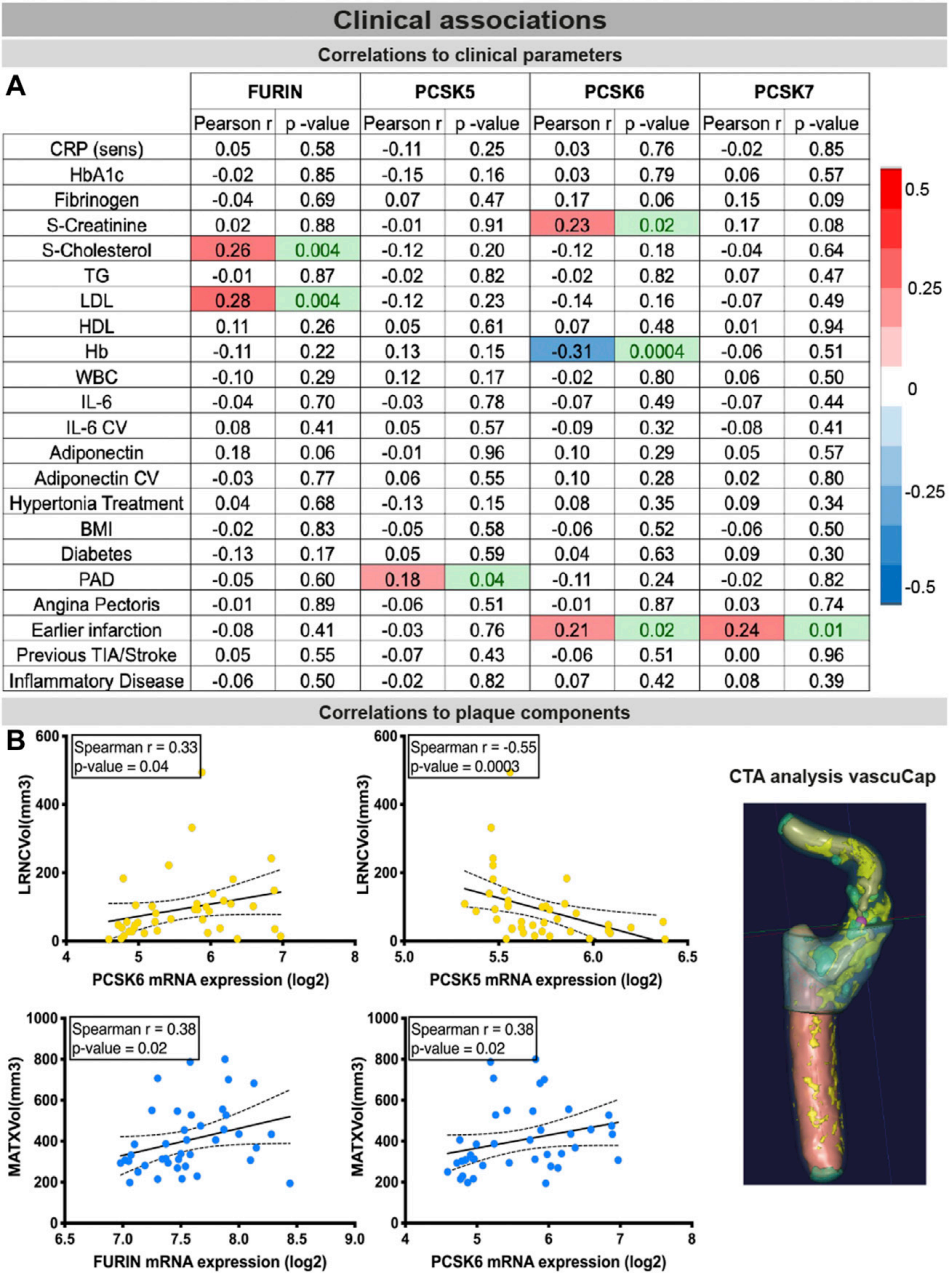
Associations of PCSKs expression in plaques with clinical data from matched patients. Pearson correlations of the FURIN and PCSKs5-7 mRNA expression in carotid plaques with biochemical blood parameters of patients undergoing carotid endarterectomy **(A)**. Expression of FURIN, PCSK5 and PCSK6 in plaque tissues correlated with different morphological plaque components, based on computed tomography image analysis with vascuCap software **(B)**. Peripheral arterial disease (PAD).

Finally, the clinical associations of selected PCSKs were investigated, by analyses of their tissue expression in connection with various patient data, blood biochemistry (Figure 7A), plaque morphological and structural components estimated by diagnostic CT images (Figure 7B). These blood parameters have been extensively linked with CVD and its comorbidities or risk factors, and widely considered to be systemic biomarkers of i.e. inflammation (CRP, IL6) or obesity and lipid levels (adiponectin, LDL, HDL), etc. Although *FURIN* was downregulated in plaques compared to normal arteries, we showed that there is a positive correlation between its plaque expression levels, serum cholesterol/LDL levels (of note, with marginal correlation also to plasma adiponectin) and total plaque MATX content in matched patients. *PCSK5* mRNA expression in plaques showed a positive link to peripheral arterial disease and a negative correlation with lipid rich necrotic core volume. *PCSK6* expression was negatively associated with plasma Hb levels, but positively with serum creatinine levels and plaque lipid rich necrotic core volume. Interestingly, both *PCSK6* and *PCSK7* plaque levels were again associated with patient symptomatology, as positive correlations were found with earlier cardiovascular events (linking marginally also with plasma fibrinogen).

## Discussion

**FIGURE 8:**
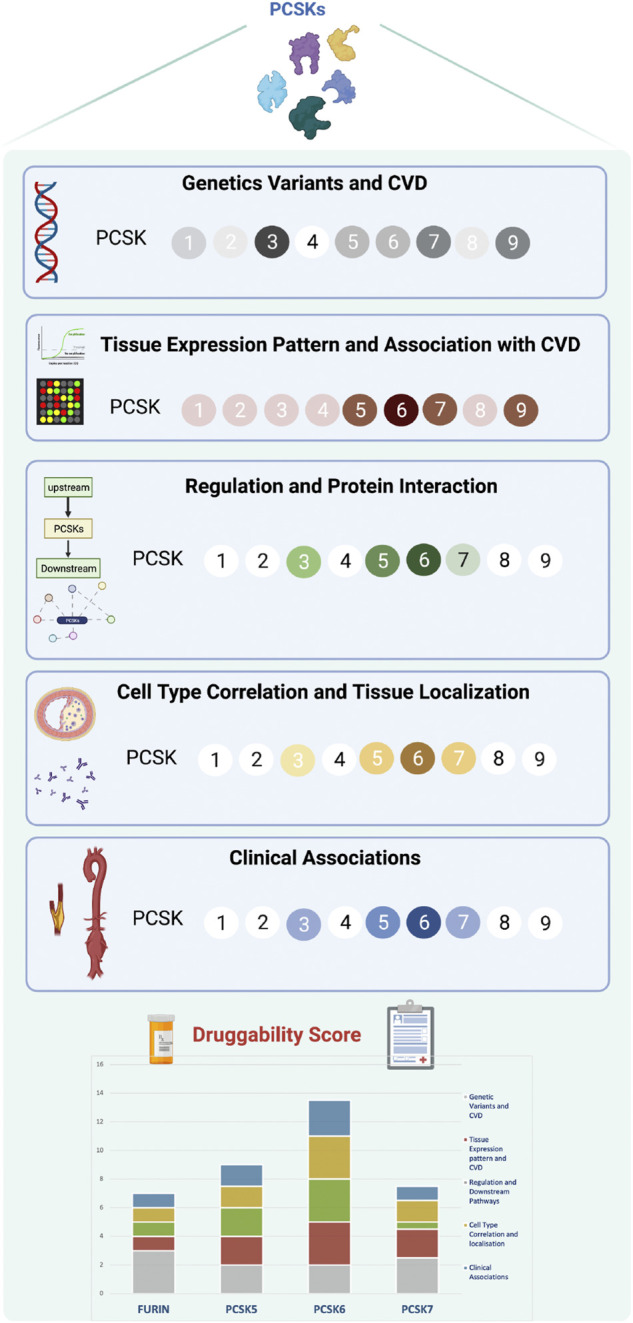
Druggability score for PCSKs. For each PCSK, cumulated evidence on genetic associations with CVD traits, expression pattern in vascular tissues, molecular regulation and clinical associations were integrated in a druggability score assessing their therapeutic targeting potential for CVD. Darker colours in each section refer to a higher score and lighter colours indicate lower score. Scoring details may be found in Supplementary Table S4. Proprotein convertase subtilisin/kexin (PCSK), cardiovascular disease (CVD).

Recent studies have shown that the presence of a genetic link between a therapeutic target and disease, in addition to establishing target specificity for the disease tissue, are the most important features for a successful drug development program (Cook et al., 2014). PCSKs have been extensively studied in cancers, where members of this family are currently explored both as biomarkers and therapeutic targets, but similar research in CVD has so far been lacking. Here we show that, with the exception of *PCSK4,* all other PCSKs genetically associate with various CVD-related phenotypes or traits, and several PCSKs are present on transcript and protein level in vascular biopsies from atherosclerosis and aortic aneurysm patients. In particular, the expression of FURIN and PCSKs5-7 correlated with key molecular pathways and mechanistic networks typical for CVD, indicating their functional relevance for the disease. Of translational interest, plaque expression of these PCSKs associated with biochemical blood parameters and morphological plaque features obtained from diagnostic CTA images, as well as patient symptomatology. Based on cumulative evidence from each section of the study, our results were integrated into a druggability score that highlighted primarily PCSK6, followed by PCSK5, PCSK7 and FURIN, as proprotein convertases with the highest novel CVD therapeutic targeting potential within this family (Figure 8).

Genetic analyses showed that variants in the genomic regions of *PCSK1* and *PCSK2* associate with CVD traits, confirming previous reports about the involvement of these genes in obesity and diabetes (O'Rahilly et al., 1995; Jackson et al., 1997), glucose homeostasis (Leak et al., 2007; Chang et al., 2015) and insulin secretion (Jonsson et al., 2012). However, due to their low expression levels in vascular tissues, the therapeutic targeting potential of these PCSKs for CVD was not further investigated here. No relevant genetic associations for *PCSK4* were observed, also restricting its therapeutic targeting potential in our context. Even though *MBTPS1* is involved in processing of transcription factors critical in the cellular response to cholesterol (Sakai et al., 1998), the ubiquitous expression of this protein creates an increased risk for numerous off target effects with respect to its therapeutic potential.

Conversely, *PCSK9* has already been strongly linked to CVD, both from genetic and functional aspects, being a major target for control of hyperlipidemia and vascular disease in clinical practice, which therefore lowered its novelty for our study. Nevertheless, recent reports showed that *PCSK9* can be found not only in hepatocytes, but in different cell types typically present within the diseased vascular wall and associated with various processes essential for CVD that go beyond its involvement in degradation of the LDLr, such as inflammation, calcification and thrombosis (Ferri et al., 2012; Ding et al., 2015; Li et al., 2017; Ding et al., 2020; Liu et al., 2020; Macchi et al., 2021; Qi et al., 2021; Lupo et al., 2022). Here we were able to confirm and extend these findings, as our study shows for the first time that despite the low mRNA expression levels observed in vascular biopsies, PCSK9 protein was abundant. Immunohistochemistry showed that both intracellular and extracellular protein were present, possibly indicating accumulation from the circulation and uptake into SMCs, macrophages and T cells. Reports that PCSK9 is not only involved in the degradation of LDLr, but also MHCI (Liu et al., 2020) and CD36 (Demers et al., 2015) molecules, could help to functionally explain our observations.

Recently, FURIN was suggested to be a potential biomarker of hypertension (He et al., 2019) and cancer (Zhou and Gao, 2021), reflecting the involvement of this broadly expressed proprotein convertase in many critical biological processes. In our study, genetic variants in *FURIN* associated with CVD and hypertension confirming previous reports (Li et al., 2010). Contrary to other reports (Stawowy et al., 2004b; Stawowy et al., 2005), we found that *FURIN* expression was decreased in carotid atherosclerotic plaques compared to healthy arteries, but it was the only PCSK that was upregulated in thoracic aneurysms, localized to macrophages, endothelial cells and T cells. This opposite expression trend possibly reflects the differences in the etiology of these diseases. The incongruency between our study and other reports, likely comes from the fact that those studies relied on a small number of biopsies from heterogeneous vascular diseases rather than large, well-defined biobanks used here. The interest in the therapeutic targeting of FURIN in atherosclerosis is growing and was recently investigated via a FURIN inhibitor in *ApoE* ^*−/−*^ and *Ldlr* ^*−/−*^ mouse models (Yakala et al., 2019). However, the systemic effect of FURIN inhibition needs to be carefully evaluated due to its ubiquitous expression, especially since high doses of FURIN inhibitors can also inhibit PCSK5/6 (Yakala et al., 2019). Additionally, FURIN inhibition could have an effect on plasma cholesterol levels, as PCSK9 is cleaved by FURIN (Oleaga et al., 2021).

PCSK7 has been involved in lowering ApoA-V levels (Ashraf et al., 2020) and recently a genetic variant in *PCSK7* was associated with dyslipidemia and NAFLD (Dongiovanni et al., 2019). In our investigation, coding variants in *PCSK7* associated with TG levels, while non-coding SNPs linked by eQTL to *PCSK7* correlated with ApoB and HDL levels. The expression of *PCSK7* was high in healthy vascular tissues, and further increased in carotid plaques, while being downregulated in AAA and TAA biopsies. We observed PCSK7 localization in SMA+ cells, although bioinformatic analyses showed negative correlations between *PCSK7* expression and typical SMC markers. This discrepancy may be explained by the process of SMC phenotypic modulation in the disease, during which typical SMC markers are downregulated (Gomez et al., 2013; Nguyen et al., 2013). In addition, the decreased expression and lack of co-localization of PCSK7 in aneurysm tissues may be explained by the characteristic loss of SMCs through apoptosis. The positive correlations observed for *PCSK7* with T cell markers, likely come from its involvement in FoxP3 processing (Elhage et al., 2015), despite a lack of their co-localization due to a relatively low amount of FoxP3+ regulatory T cells in carotid plaques (Elhage et al., 2015). Together, our PCSK7 data indicates that this gene could be essential for the vessel function, connecting lipid genetics with SMCs and T cells in the vessel wall.

Studies of PCSK5 in CVD are restricted to reports associating its genetic variants with HDL levels (Iatan et al., 2009) and describing its role in heart development (Szumska et al., 2017). PCSK5 processes endothelial and lipoprotein lipase, critical enzymes in the metabolism of TGs (Choi and Korstanje, 2013). We observed a decreased *PCSK5* expression in atherosclerotic plaques and AAA coupled to the positive correlation with classical SMC markers, in agreement with another study showing that PCSK5 is found in healthy rat SMCs (Stawowy et al., 2001)*.* Despite lower mRNA levels, PCSK5 staining was present in SMCs in plaques but not in AAA tissues, likely again due to SMC apoptosis. PCSK5 has been shown to be important for SMC proliferation since it is involved in activation of the αv integrin along with FURIN(Stawowy et al., 2003). In addition, PCSK5 participates in the cleavage of several members of the TGF-beta superfamily, such as GDF11 (Tsuda et al., 2011), GDF15 (Li et al., 2018), BMPs (Constam et al., 1996) as well as several MMPs (Stawowy et al., 2004a), all essential factors in CVD development and progression.

Genetic evidence presented in this study showed that a SNP in PCSK6 locus associates with neutrophil count, a cell type which plays a major role in the AAA (Cohen et al., 1991). The expression of *PCSK6* was significantly increased in both AAA and carotid biopsies compared to healthy arteries, confirming and extending previous investigations from our group and others (Perisic et al., 2013; Jorgensen and Bennett, 2020; Rykaczewska et al., 2020; Testa et al., 2021). Additionally, our previous and current work showed that PCSK6 is primarily expressed by phenotypically modulated SMCs (Perisic et al., 2013), but also to some extent by macrophages, T cells and endothelial cells in plaques. Interestingly, this is a similar expression pattern as observed for PCSK7, however with the important difference that PCSK7 levels were found to be high, while PCSK6 was low in healthy arterial tissue, indicating that PCSK6 would be more suitable for therapeutic targeting. We and others have shown that PCSK6 is involved in the cleavage of MMPs (Rykaczewska et al., 2020; Testa et al., 2021) that play a pivotal role in CVD in general (Knox et al., 1997; Annabi et al., 2002). The interest in PCSK6 as a therapeutic target in CVD could be attributed to its processing of various proteins involved in different stages of atherosclerosis development, such as suppressing the activity endothelial and lipoprotein lipase, preventing the release of free fatty acids from HDL particles (Choi and Korstanje, 2013), processing GDF-15 that increases inflammatory response (Wang et al., 2019) as well as PDGFB and MMP14 responsible for SMC migration (Rykaczewska et al., 2020). PCSK6, like PCSK5 and FURIN, is known to cleave several members of the TGF-beta superfamily and several BMPs (Constam and Robertson, 1999), which was also confirmed in our pathway analysis, suggesting a possible new role in vascular calcification.

Exploration of the therapeutic targeting potential for PCSKs revealed certain similarities among the family members, both when it comes to the molecular mechanisms involving these PCSKs and from a clinical perspective. The analysis of transcription factors upstream from the FURIN and PCSK5-7 correlated networks, identified SP1 as a common regulatory transcription factor. SP1 is known to transduce signals from the TGFB superfamily and increase vascular calcification, a key feature of ageing vessels, by promoting BMP2 transcription (Zhang et al., 2018). Our study also identified specific atherosclerosis-related transcription factors KLF4, KLF11 and BCLAF1 upstream from FURIN, PCSK6 or PCSK7. KLF4 and BCLAF1 initiate SMC phenotypic switch in atherosclerosis by inducing SMC lipid transdifferentiation (Rykaczewska et al., 2022), whereas KLF11 has been shown to inhibit arterial thrombosis (Liang et al., 2019). The pathway analysis, along with the exploration of genetic associations, showed another commonality among FURIN, PCSK5 and PCSK6 when it comes to the regulation of lipid metabolism, indicating that PCSK9 is not the only PCSK involved in this process. These observations may be attributed to the role of these PCSKs in regulating the TG metabolism (Choi and Korstanje, 2013), but also to the reported capacity for inactivation of PCSK9 by both FURIN and PCSK5 (Choi and Korstanje, 2013). This highlights a growing interest in these PCSKs as novel targets for lipid lowering therapy, beyond the established interest in PCSK9. Inflammatory pathways (Libby, 2021) were also seen to be upregulated when considering PCSK5 and PCSK6 correlated transcripts in carotid plaques, offering a potential additional benefit when it comes to the application of therapies targeting these PCSKs. Specifically for PCSK6, the correlated transcripts were enriched for ECM disassembly pathways, which corroborates our previous research associating PCSK6 with vascular remodeling (Rykaczewska et al., 2020) and underlines a potential pleiotropic benefit that could be obtained from targeting PCSK6 by modulating SMC function.

From a clinical perspective, our investigations opened for several new notions in PCSK biology that need further exploration. FURIN plaque mRNA levels were shown to correlate to systemic cholesterol and LDL levels, but did not associate with the LRNC size and local cholesterol accumulation within the plaque. A large number of FURIN substrates include matrix proteases (Tian et al., 2011), such as MMP2, which could explain its correlation with plaque MATX in our study. Increased *PCSK7* expression in carotid plaques and in patients with previous myocardial infarction is a novel finding requiring further studies considering recent evidence linking PCSK7 variants to acute coronary syndrome (Vargas-Alarcon et al., 2021). *PCSK5* expression negatively correlated with LRNC volume (Choi and Korstanje, 2013) which, in combination with its reduced expression in plaques and especially those from symptomatic patients, might suggest a protective role of PCSK5 in CVD. Among the highly ranked PCSKs, PCSK6 appeared to be associated with the highest number of clinical parameters. Not only were PCSK6 plaque levels increased in patients with previous myocardial infarction and patients undergoing carotid endarterectomy with stroke symptoms, but the positive correlation observed with MATX components and LRNC volume suggests a possible role of PCSK6 in plaque vulnerability (Testa et al., 2021; Perisic et al., 2013). Our data aligns with a recent report by Kuhn *et al* identifying PCSK6 as a novel player in cardiac remodeling after myocardial infarction (Kuhn et al., 2020), but also with a more recent report showing that *Pcsk6* deficiency results in cardiomyocyte senescence (Zhan et al., 2022) that confirmed our findings in primary vascular SMCs isolated from *Pcsk6* knockouts (Rykaczewska et al., 2020). Interestingly, *PCSK6* expression also associated with serum creatinine levels suggesting an unexplored role in renal function.

Despite large R&D investments in both academia and industry and numerous expensive proof-of-concept studies, the output of new drugs in the CVD space has remained largely static over the past decades. In this context, we reasoned that a defined, comprehensive framework for target assessment could promote more informed decision-making, enabling either the termination of targets based on clearly defined go/no-go criteria, or further investment in those projects which demonstrate the attributes and clinical signals that lead to increased confidence. Inspired by the previously suggested five-dimensional (5D) drug development pipeline (Cook et al., 2014), we designed a new ‘molecular’ 5D framework for early target assessment dedicated to examining the following points: i) genetic link with the disease, ii) target tissue, iii) target cell type, iv) function and mechanism, and v) target associations with patient clinical data. An additional relevant dimension taken into account was the overall IP status surveyed in patent databases. This revealed high IP activity in the field of PCSKs, with patents ranging from biomarkers of diabetes (PCSK1 and PCSK2), to biomarkers and drug targets in cancer (FURIN and PCSK6), with the highest number of patents expectedly related to PCSK9 (Supplementary Table S6). Applying this “molecular” 5D rationale, altogether highlighted primarily PCSK6, followed by PCSK5, PCSK7 and FURIN, as proprotein convertases that successfully bridge across the genetic causality, molecular mechanisms directed to the target tissue/cell and clinical patient phenotypes. Considering the translational potential of our ‘molecular’ 5D framework, we propose that it could be broadly applied in the early stages of a drug target selection process, even before proof-of-concept and experimental validation studies.

### Limitations

Although we had access to some of the largest vascular disease biobanks worldwide, limitations of our study are still related to the cohort sizes, which have disabled stratification of data with respect to gender, medication, smoking and other relevant factors. The lack of healthy controls and especially disease progression data/biopsies for establishing a longitudinal association between the gene and the disease, is also an important limitation. From a technical perspective, the scRNAseq data generated from coronary plaques was used to deconvolve BiKE carotid plaque microarrays, which could be seen positively as extrapolation of results between the carotid and coronary disease. However, this also increases the risk of overestimating some cell populations and underestimating others, while neglecting possibly unique cell sub-phenotypes that are present in carotid compared to coronary plaques. Furthermore, the immune signature also used in the deconvolution may not be entirely suitable for extrapolating into complex tissues.

## Conclusion

In summary, we established a novel “molecular” 5D framework, intended to complement the existing 5D framework proposed for drug development, especially for systematic early assessment of the therapeutic target potential in CVD, and conducted the first integrative study of the proprotein convertase family in this context. Our results using this translational pipeline, revealed primarily PCSK6, followed by PCSK5, PCSK7 and FURIN, as proprotein convertases with the highest novel therapeutic potential. The ‘molecular’ 5D framework is designed to challenge, validate or invalidate the scientific hypothesis around a certain target at its inception, placing a focus on a strong target rationale. Our framework does not directly address potential safety concerns that would be associated with systemic targeting, such as unwanted effect on blood pressure or lipid levels in patients already prescribed extensive anti-hypertensive and lipid lowering medications. We suggest that local tissue delivery should be considered, when possible, to mitigate these risks. Looking to the future, independent validations across human cohorts, engaging diseases that share common molecular mechanisms (here depicted as atherosclerosis, AAA and TAA), may lead to more effective translation of science into medicines at the interface and in collaboration between academia and industry.

## Data Availability

The datasets presented in this study can be found in online repositories. The names of the repository/repositories and accession number(s) can be found below: https://www.ncbi.nlm.nih.gov/, GSE21545, GSE125771, GSE26155.
