# Supplementary material for: Therapeutic potential of the Proprotein Convertase Subtilisin/Kexin family in vascular disease
Source: Front Pharmacol. 2022 Sep 15;13:988561. doi: 10.3389/fphar.2022.988561 (PMC9520287; doi:10.3389/fphar.2022.988561)
Supplement: Supplementary file 1 [file DataSheet1.PDF]

Supplementary Material

1 Supplementary Figures and Tables

Supplementary Figure 1:

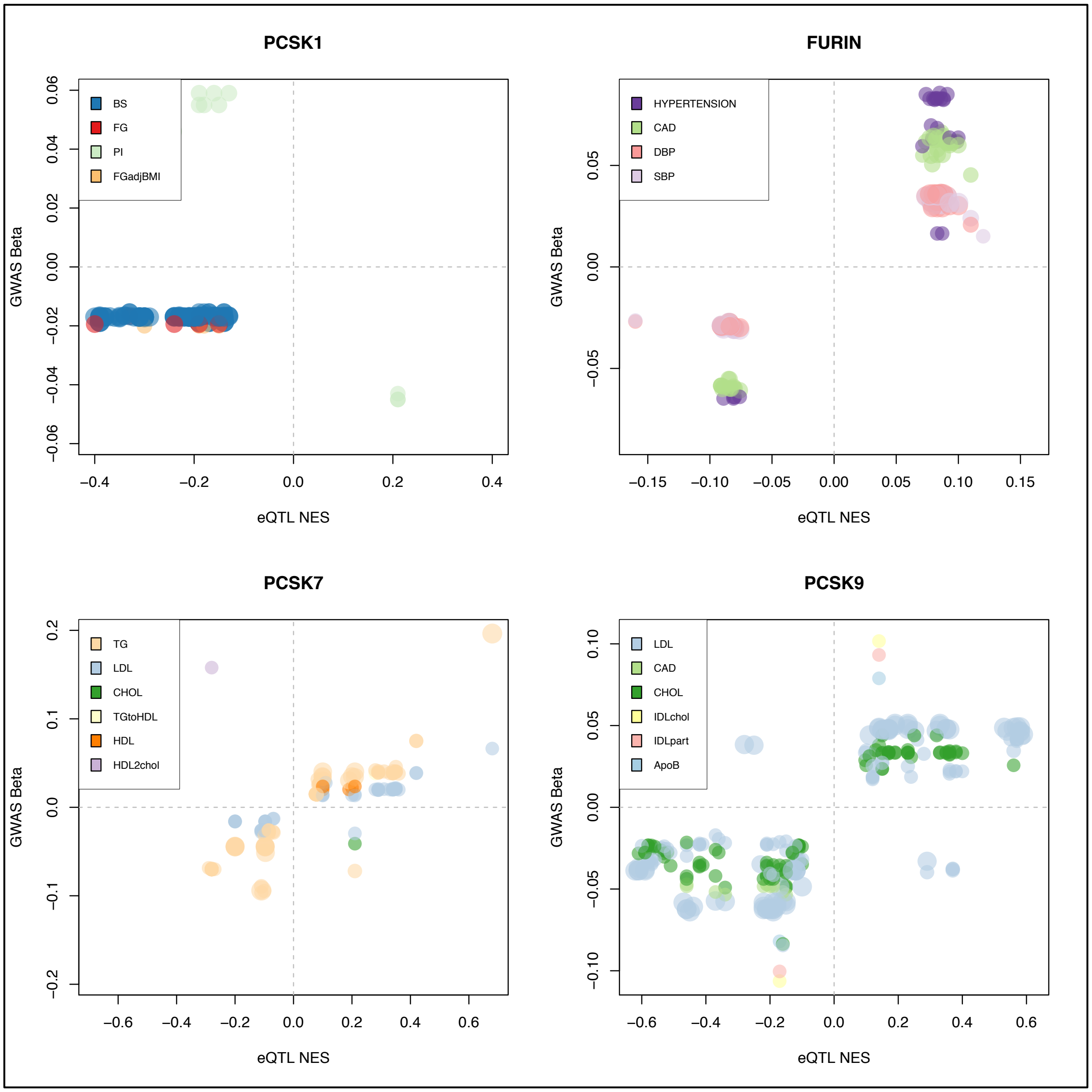

Supplementary Figure 1: Directionality of cardiovascular trait associations with the PCSK family. Integration of GWAS beta values and eQTL Normalised Effect Size (NES) for the same variants allows inference of the directionality of how each gene impacts on the cardiovascular trait. Points occurring in the upper right and lower left quadrants indicate a positive association of the gene with the trait, suggesting that the gene is detrimental (i.e., higher gene expression results in more disease or higher blood pressure). Points occurring in the upper left or lower right quadrants indicate the opposite, an inverse association, suggesting that the gene might be protective.

Supplementary Figure 2:

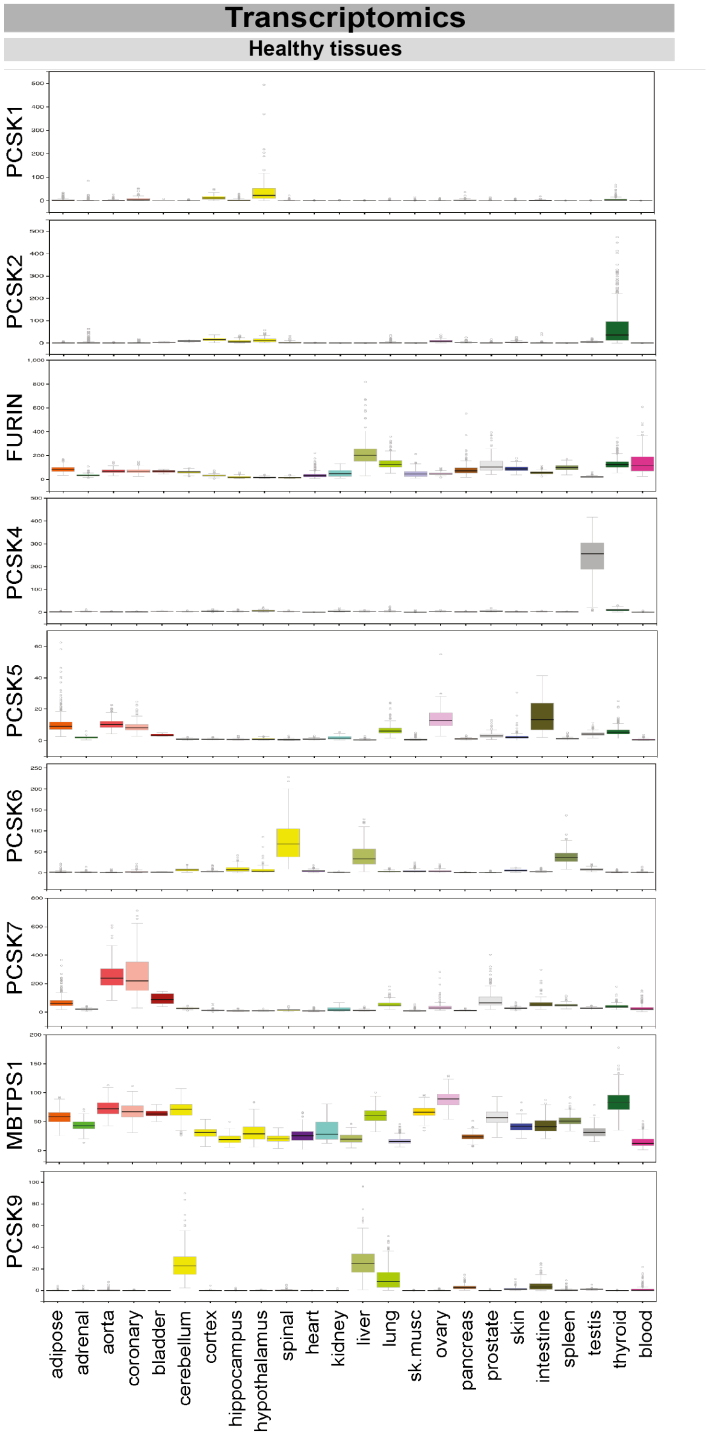

Supplementary Figure 2. Expression of the PCSK family members in healthy human tissues. mRNA levels of the different members of the PCSK family across healthy tissues, based on the public resource GTex RNAseq data.

Supplementary Figure 3:

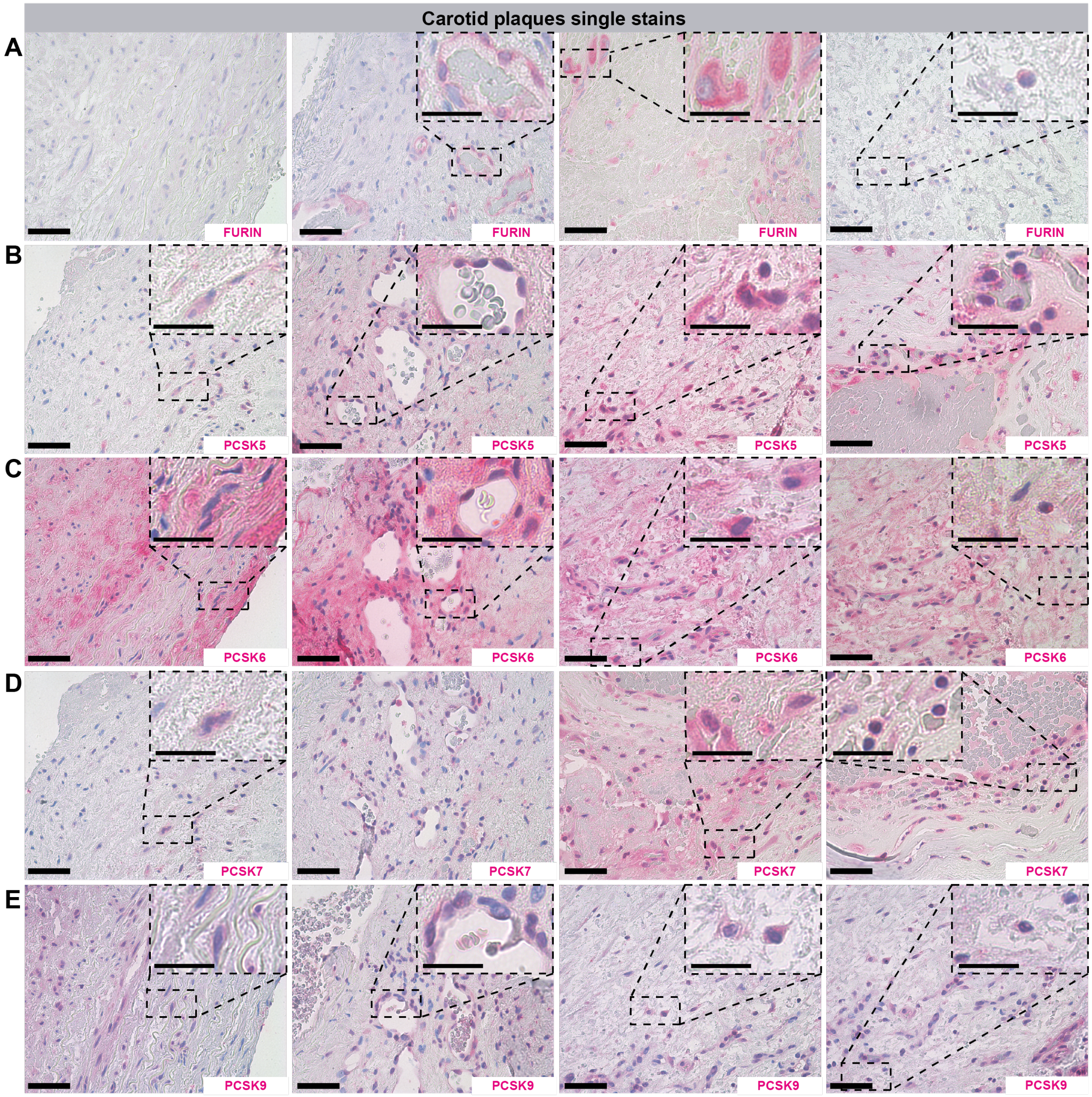

Supplementary Figure 3. Localization of FURIN, PCSK5-7 and PCSK9 in carotid plaques. Single immunohistochemistry stainings of FURIN (A), PCSK5 (B), PCSK6 (C), PCSK7 (D) and PCSK9 (E). Scale bar represents 50 μm and in the enlarged insets 25 μm.

Supplementary Figure 4:

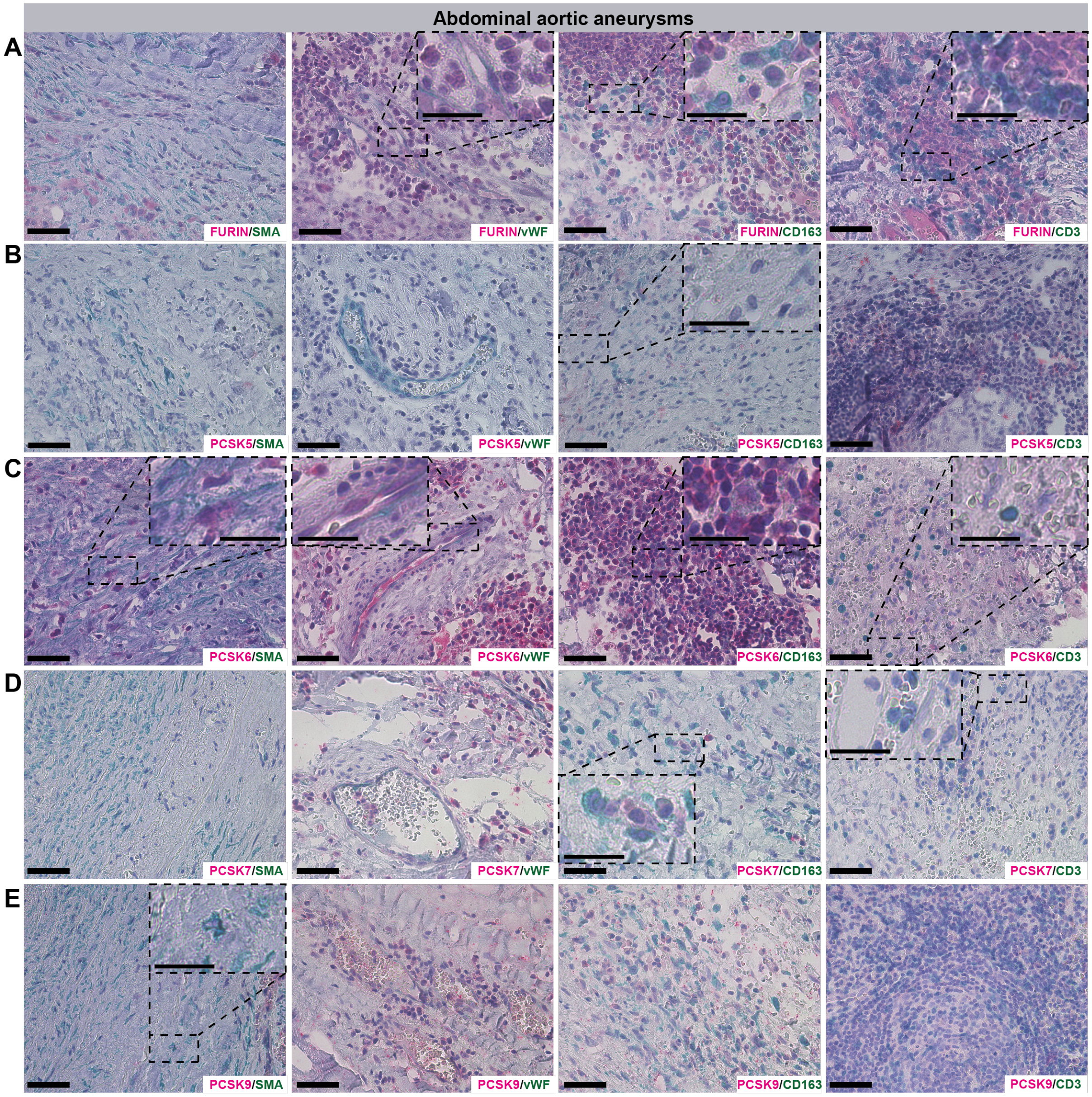

Supplementary Figure 4. Co-localisation of FURIN, PCSK5-7 and PCSK9 with major cell types in aortic aneurysm. Immunohistochemistry co-stainings of classical markers CD3 (T cells), CD163 (macrophages), SMA (smooth muscle cells) and vWF (endothelial cells) with FURIN **(A)**, PCSK5 **(B)**, PCSK6 **(C)**, PCSK7 **(D)** and PCSK9 **(E)**. Scale bar represents 50 μm and in the enlarged insets 25 μm.

Supplementary Table I:

Gene	***Role in cardiovascular disease***	***Associated SNPs***	***Reference***	***#References*** ***PCSK cardiovascular Pubmed 2021***
PCSK1	Early-onset obesity due to impaired glucose homeostasis.	*rs6232, rs6234/6235*	*(O'Rahilly et al., 1995), (Jackson et al., 1997)*	*11*
PCSK2	Genetic associations with diabetes and myocardial infarction.	*rs2021785, rs6080699*	*(Leak et al., 2007), (Fujimaki et al., 2010)*	*6*
FURIN	Present in atherosclerotic plaques. GWAS hit for CAD. Genetic variations associate with hypertension.	*rs17514846, rs747304, rs8027450, rs4932373*	*(Stawowy et al., 2004; Stawowy et al., 2005),(Ghosh et al., 2015), (Li et al., 2010)*	*166*
PCSK4	None reported.			*2*
PCSK5	Present in atherosclerotic plaques. Involved in HDL metabolism.		*(Stawowy et al., 2004; Stawowy et al., 2005), (Jin et al., 2007)*	*11*
PCSK6	Upregulated in unstable carotid atherosclerotic plaques. Regulates blood pressure. Possible role in HDL homeostasis. Upregulated by oxysterols present in plaques. Released by cardiomyocytes in response to hypoxia and increases fibrosis after MI		*(Perisic et al., 2013), (Rykaczewska et al., 2020), (Chen et al., 2015), (Jin et al., 2007), (Testa et al., 2021), (Kuhn et al., 2020)*	*26*
PCSK7	SNPs associated with small dense LDL cholesterol and TG levels.	*rs508487, rs1784119*	*(Hoogeveen et al., 2014)*	*11*
MBTPS1	Role in lipid homeostasis through cleavage of SREBPs.		*(Sakai et al., 1998)*	*4*
PCSK9	Activating mutations lead to excessive LDLR degradation and increased cholesterol levels.	*rs28942111, rs505151*	*(Abifadel et al., 2003)*	*2,661*

Supplementary Table I: Summary of publications addressing the role of PCSKs in cardiovascular disease until Dec 2021. Source Pubmed, search words used *PCSK* and *cardiovascular*.

Supplementary Table II:

	**Genetic Variants and CVD**		
	**Coding SNPs**	**non-coding SNPs linked to eQTL**	**Average**
Figures/Results	*Figure 2A and 2B*	*Figure 2C*	
PCSK1	2	1	**1,5**
PCSK2	1	NA	**1**
**PCSK3**	3	3	**3**
PCSK4	0	NA	**0**
**PCSK5**	2	NA	**2**
**PCSK6**	2	NA	**2**
**PCSK7**	3	2	**2,5**
PCSK8	1	NA	**1**
PCSK9	2	3	**2,5**
**Scoring**	*0 no association with CVD or CVD risk factor*	*0 No association*	
	1 moderate association with CVD risk factors	*1 Associated with medium p values CVD risk factors*	
	2 strong association with CVD risk factors	*2 Associated with strong p values CVD risk factors*	
	3 strong association with CVD and CVD risk factors	*3 Associated with CVD and CVD risk factors*	
	**Tissue Expression pattern and CVD**		
	**Public RNA seq data**	**BiKE RNA transcriptomics/Proteomics**	**Average**
Figures/Results	*Supp Figure 1*	*Figure 3*	
PCSK1	1	1	**1**
PCSK2	1	1	**1**
**PCSK3**	0	2	**1**
PCSK4	1	1	**1**
**PCSK5**	2	2	**2**
**PCSK6**	3	3	**3**
**PCSK7**	2	2	**2**
PCSK8	0	2	**1**
PCSK9	3	1	**2**
**Scoring**	*0 ubiquitous expression*	*0 not expressed*	
	1 Detected in irrelevant tissue (for CVD)	*1 Expressed at low/moderate levels in disease tissue without any sig change between disease and control*	
	2 restricted expression pattern in CVD tissue	*2 Expressed at high level in disease tissue with sig change between disease and control (either up or down)*	
	3 restricted expression pattern in CVD tissue and CVD relevant tissue (such as the liver)	*3 Expressed at high level in disease tissue with sig change between disease and control (either up or down) and shows differential expression in symptomatic patients*	
	**Only pursued FURIN, PCSK5,PCSK6 and PCSK7**		
	**Regulation and Downstream Pathways**		
	**Transcription factors**	**GSEA**	**Average**
Figures/Results	*Figure 4B*	*Figure 4C*	
PCSK1			
PCSK2			
**PCSK3**	1	1	**1**
PCSK4			
**PCSK5**	2	2	**2**
**PCSK6**	3	3	**3**
**PCSK7**	1	0	**0,5**
PCSK8			
PCSK9			
**Scoring**	*0 popular common TF involved in the regulation of associated genes*	*0 No CVD related pathways*	
	1 Some common TF involved in the regulation of associated genes	*1 Low number CVD related pathways*	
	2 Some unique TF involved in the regulation of associated genes	*2 Moderate number of CVD related pathways*	
	3 Evidenced unique TF involved in the regulation of associated genes	*3 High number of CVD related pathways*	
	**Cell Type Correlation and localisation**		
	**Cell type correlation**	**Cell localization by IHC**	**Average**
Figures/Results	*Figure 5*	*Figure 6*	
PCSK1			
PCSK2			
**PCSK3**	1	1	**1**
PCSK4			
**PCSK5**	1	2	**1,5**
**PCSK6**	3	3	**3**
**PCSK7**	2	1	**1,5**
PCSK8			
PCSK9		2	
**Scoring**	*0 No correlation in carotid disease arteries*	*0 No presence in carotid plaques*	
	1 Week correlation with inflammatory cells in carotid arteries	*1 Week co-localization with cells in carotid plaque (EC, Ly, macrophages, SMC)*	
	2 Moderate correlation with inflammatory cells in carotid arteries	*2 Moderate co-localization with cells in carotid plaque*	
	3 High correlation with inflammatory cells in carotid arteries	*3 High co-localization with cells in carotid plaque*	
	**Clinical Associations**		
	**CAD and CAD risk factors**	**Correlation with MATX, LRNC, CAL**	**Average**
Figures/Results	*Figure 7A*	*Figure 7B,C,D,E*	
PCSK1			
PCSK2			
**PCSK3**	1	1	**1**
PCSK4			
**PCSK5**	2	1	**1,5**
**PCSK6**	3	2	**2,5**
**PCSK7**	2	0	**1**
PCSK8			
PCSK9			
**Scoring**	*0 No association*	*0 No association*	
	1 Association with CVD Risk factors	*1 Association with 1 factor*	
	2 Association with CVD	*2 Association with 2 factors*	
	3 Association with CVD + CVD Risk factors	*3 Association with 3 factors*	

Supplementary Table II: Overview of the scoring system used to evaluate the druggability potential of PCSKs in CVD.

Supplementary Table III:

varId	alt	beta	chromosome	n	pValue	phenotype	position	reference	stdErr	zScore	consequence	dbSNP	nearest
5:95728974:G:C	C	0.017	5	3737160	8.012e-26	BMI	95728974	G	9,00E-04	10.507	missense_variant	rs6234	PCSK1
5:95728898:C:G	G	3,00E-04	5	1432280	5.58e-23	HEIGHT	95728898	C	2,00E-04	9.871	missense_variant	rs6235	PCSK1
5:95728898:C:G	G	-0.0171	5	468542	8.26e-23	BS	95728898	C	0.0016	-9.831	missense_variant	rs6235	PCSK1
5:95728974:G:C	C	-0.0171	5	468542	9.169e-23	BS	95728974	G	0.0016	-9.821	missense_variant	rs6234	PCSK1
5:95728898:C:G	G	0.0169	5	3723380	2.685e-22	BMI	95728898	C	9,00E-04	9.712	missense_variant	rs6235	PCSK1
5:95728974:G:C	C	4,00E-04	5	1438460	9.332e-20	HEIGHT	95728974	G	2,00E-04	9.096	missense_variant	rs6234	PCSK1
5:95728898:G:C	C	-0.058	5	10701	7.708e-14	PI	95728898	G	0.0078	-7.475	missense_variant	NA	PCSK1
5:95728974:G:C	C	0.058	5	10701	1.65e-13	PI	95728974	G	0.0078	7.374	missense_variant	rs6234	PCSK1
5:95728974:G:C	C	-0.0214	5	66151	4.324e-10	FGadjBMI	95728974	G	0.0035	-6.242	missense_variant	rs6234	PCSK1
5:95728974:G:C	C	-0.0178	5	147738	7.026e-10	FG	95728974	G	0.0025	-6.165	missense_variant	rs6234	PCSK1
5:95728898:G:C	C	0.02	5	58074	3.148e-08	FGadjBMI	95728898	G	0.0036	5.533	missense_variant	NA	PCSK1
15:101910550:G:A	A	0.0103	15	602760	2.944e-08	NeutCount	101910550	G	0.0018	5.545	missense_variant	rs20543	PCSK6
11:117076972:C:A	A	0.0507	11	336679	2.59e-20	TG	117076972	C	0.0055	9.235	missense_variant	rs45574931	PCSK7
11:117100257:C:G	G	0.1132	11	326434	2.12e-17	TG	117100257	C	0.013333	8.490212255	missense_variant	rs11542139	PCSK7
11:117076741:G:A	A	-0.0359	11	439488	3.76e-08	TG	117076741	G	0.0065	-5.502	missense_variant	rs201598301	PCSK7
1:55505647:G:T	T	-0.4049	1	334901	8.13e-302	LDL	55505647	G	0.0084	-37.133	missense_variant	rs11591147	PCSK9
1:55518467:A:G	G	-0.0487	1	459525	3.73e-281	LDL	55518467	A	0.002	-35.83	splice_region_variant	rs2495477	PCSK9
1:55509585:C:T	T	-0.5425	1	44985	7.62e-77	LDL	55509585	C	0.0292	-18.554	missense_variant	rs151193009	PCSK9
1:55509585:C:T	T	-0.5081	1	46025	3.96e-70	CHOL	55509585	C	0.0287	-17.703	missense_variant	rs151193009	PCSK9
1:55505647:G:T	T	-0.4304	1	21060	8.933e-59	CHOL	55505647	G	0.0264	-16.165	missense_variant	rs11591147	PCSK9
1:55518316:C:T	T	-0.0352	1	459525	1.382e-57	LDL	55518316	C	0.0021	-15.995	splice_region_variant	rs2483205	PCSK9
1:55529187:G:A	A	-0.0755	1	409905	6.964e-42	LDL	55529187	G	0.0056	-13.559	missense_variant	rs505151	PCSK9
1:55505647:G:T	T	-0.4202	1	10987	8.766e-35	IDLchol	55505647	G	0.034	-12.303	missense_variant	rs11591147	PCSK9
1:55505647:G:T	T	-0.3968	1	10984	3.212e-31	IDLpart	55505647	G	0.034	-11.621	missense_variant	rs11591147	PCSK9
1:55505647:G:T	T	-0.2341	1	649567	1.023e-30	CAD	55505647	G	0.0202	-11.522	missense_variant	rs11591147	PCSK9
1:55518467:A:G	G	-0.0382	1	205969	6.891e-30	CHOL	55518467	A	0.0032	-11.356	splice_region_variant	rs2495477	PCSK9
1:55505647:G:T	T	-0.3326	1	14459	3.661e-28	ApoB	55505647	G	0.0302	-11.004	missense_variant	rs11591147	PCSK9
1:55505647:G:T	T	-0.6523	1	56375	4.206e-28	Dyslipid	55505647	G	0.0593	-10.991	missense_variant	rs11591147	PCSK9
1:55518316:C:T	T	-0.037	1	205969	8.06e-27	CHOL	55518316	C	0.0033	-10.722	splice_region_variant	rs2483205	PCSK9
1:55524197:A:G	G	0.0331	1	457431	1.42e-25	LDL	55524197	A	0.0026	10.453	missense_variant	rs540796	PCSK9
1:55524237:G:A	A	0.0336	1	456084	9.044e-25	LDL	55524237	G	0.0027	10.276	missense_variant	rs562556	PCSK9
1:55505647:G:T	T	-0.3084	1	10984	2.681e-19	RemnantC	55505647	G	0.0343	-8.981	missense_variant	rs11591147	PCSK9
1:55518467:A:G	G	-0.1063	1	10987	2.336e-14	IDLchol	55518467	A	0.0139	-7.631	splice_region_variant	rs2495477	PCSK9
1:55551718:G:A	A	0.0413	1	337039	9.827e-14	LDL	55551718	G	0.0055	7.443	splice_region_variant	rs11583974	PCSK9
1:55518467:A:G	G	-0.1004	1	10984	5.683e-13	IDLpart	55518467	A	0.0139	-7.208	splice_region_variant	rs2495477	PCSK9
1:55518467:A:G	G	-0.082	1	14459	1.989e-11	ApoB	55518467	A	0.0122	-6.707	splice_region_variant	rs2495477	PCSK9
1:55505647:G:T	T	-0.2086	1	10970	7.529e-10	SM	55505647	G	0.0339	-6.155	missense_variant	rs11591147	PCSK9
1:55518316:C:T	T	-0.0822	1	10987	3.069e-09	IDLchol	55518316	C	0.0139	-5.928	splice_region_variant	rs2483205	PCSK9
1:55524197:A:G	G	0.0976	1	14459	6.89e-09	ApoB	55524197	A	0.0168	5.794	missense_variant	rs540796	PCSK9
1:55524237:G:A	A	0.0974	1	14459	7.332e-09	ApoB	55524237	G	0.0168	5.783	missense_variant	rs562556	PCSK9
1:55524197:A:G	G	0.11	1	10987	8.033e-09	IDLchol	55524197	A	0.0191	5.768	missense_variant	rs540796	PCSK9
1:55524237:G:A	A	0.11	1	10987	8.033e-09	IDLchol	55524237	G	0.0191	5.768	missense_variant	rs562556	PCSK9
1:55505668:C:G	G	-0.0342	1	153537	1.235e-08	LDL	55505668	C	0.0062	-5.51612903225806	missense_variant	rs11583680	PCSK9
1:55524197:A:G	G	0.1078	1	10984	1.536e-08	IDLpart	55524197	A	0.0191	5.657	missense_variant	rs540796	PCSK9
1:55524237:G:A	A	0.1078	1	10984	1.536e-08	IDLpart	55524237	G	0.0191	5.657	missense_variant	rs562556	PCSK9
1:55505647:G:T	T	-0.2127	1	8751	2.495e-08	n6FA182	55505647	G	0.0381	-5.574	missense_variant	rs11591147	PCSK9
1:55505647:G:T	T	0.0157	1	349743	3.176e-08	BILIRUBIN	55505647	G	0.0028	5.531	missense_variant	rs11591147	PCSK9
1:55518467:A:G	G	-0.0775	1	10984	3.237e-08	RemnantC	55518467	A	0.014	-5.528	splice_region_variant	rs2495477	PCSK9

Supplementary Table III: Coding variants around each PCSK genetic locus (+/-50KB) with a known GWAS association.

Supplementary Table IV:

dbSNP	beta	pValue	phenotype	consequence	Gene.Symbol	P.Value	NES	Tissue	representative
rs10036439	-0.017	2.76e-23	BS	intron_variant	PCSK1	2.1e-10	-0.3	Heart - Atrial Appendage	FALSE
rs10036439	-0.017	2.76e-23	BS	intron_variant	PCSK1	3.3e-06	-0.15	Artery - Tibial	FALSE
rs10036439	-0.017	2.76e-23	BS	intron_variant	PCSK1	1.4e-05	-0.18	Artery - Aorta	FALSE
rs10036439	-0.017	2.76e-23	BS	intron_variant	PCSK1	3.5e-10	-0.19	Adipose - Subcutaneous	FALSE
rs10213823	-0.0171	1.886e-23	BS	intron_variant	PCSK1	1.7e-10	-0.31	Heart - Atrial Appendage	TRUE
rs10213823	-0.0171	1.886e-23	BS	intron_variant	PCSK1	8.1e-06	-0.15	Artery - Tibial	TRUE
rs10213823	-0.0171	1.886e-23	BS	intron_variant	PCSK1	3.1e-07	-0.21	Artery - Aorta	TRUE
rs10213823	-0.0171	1.886e-23	BS	intron_variant	PCSK1	1.8e-10	-0.2	Adipose - Subcutaneous	TRUE
rs10476552	-0.017	2.634e-23	BS	intron_variant	PCSK1	1.7e-06	-0.2	Artery - Aorta	FALSE
rs10476552	-0.017	2.634e-23	BS	intron_variant	PCSK1	7,00E-06	-0.15	Artery - Tibial	FALSE
rs10476552	-0.017	2.634e-23	BS	intron_variant	PCSK1	1.7e-10	-0.31	Heart - Atrial Appendage	FALSE
rs10476552	-0.017	2.634e-23	BS	intron_variant	PCSK1	2.7e-10	-0.2	Adipose - Subcutaneous	FALSE
rs10476553	-0.017	2.139e-23	BS	intron_variant	PCSK1	8.1e-06	-0.15	Artery - Tibial	FALSE
rs10476553	-0.017	2.139e-23	BS	intron_variant	PCSK1	1.7e-10	-0.31	Heart - Atrial Appendage	FALSE
rs10476553	-0.017	2.139e-23	BS	intron_variant	PCSK1	3.1e-07	-0.21	Artery - Aorta	FALSE
rs10476553	-0.017	2.139e-23	BS	intron_variant	PCSK1	1.8e-10	-0.2	Adipose - Subcutaneous	FALSE
rs10515237	-0.0154	7.071e-19	BS	intron_variant	PCSK1	5.7e-11	-0.33	Heart - Atrial Appendage	FALSE
rs10515237	-0.0154	7.071e-19	BS	intron_variant	PCSK1	1.5e-05	-0.19	Artery - Aorta	FALSE
rs10515237	-0.0154	7.071e-19	BS	intron_variant	PCSK1	2.9e-05	-0.14	Artery - Tibial	FALSE
rs10515237	-0.0154	7.071e-19	BS	intron_variant	PCSK1	1.6e-07	-0.17	Adipose - Subcutaneous	FALSE
rs10515237	-0.0168	1.694e-14	FG	intron_variant	PCSK1	5.7e-11	-0.33	Heart - Atrial Appendage	FALSE
rs10515237	-0.0168	1.694e-14	FG	intron_variant	PCSK1	1.5e-05	-0.19	Artery - Aorta	FALSE
rs10515237	-0.0168	1.694e-14	FG	intron_variant	PCSK1	2.9e-05	-0.14	Artery - Tibial	FALSE
rs10515237	-0.0168	1.694e-14	FG	intron_variant	PCSK1	1.6e-07	-0.17	Adipose - Subcutaneous	FALSE
rs11395420	-0.0187	3.724e-27	BS	intron_variant	PCSK1	4.4e-15	-0.39	Heart - Atrial Appendage	TRUE
rs11395420	-0.0187	3.724e-27	BS	intron_variant	PCSK1	2.6e-05	-0.14	Artery - Tibial	TRUE
rs11395420	-0.0187	3.724e-27	BS	intron_variant	PCSK1	7.7e-08	-0.17	Adipose - Subcutaneous	TRUE
rs11395420	-0.0187	3.724e-27	BS	intron_variant	PCSK1	5.5e-06	-0.19	Artery - Aorta	TRUE
rs12109272	-0.0169	8.959e-23	BS	intron_variant	PCSK1	6.6e-08	-0.23	Artery - Aorta	FALSE
rs12109272	-0.0169	8.959e-23	BS	intron_variant	PCSK1	4.8e-06	-0.15	Artery - Tibial	FALSE
rs12109272	-0.0169	8.959e-23	BS	intron_variant	PCSK1	3.9e-09	-0.18	Adipose - Subcutaneous	FALSE
rs12109272	-0.0169	8.959e-23	BS	intron_variant	PCSK1	6.1e-16	-0.38	Heart - Atrial Appendage	FALSE
rs12332295	-0.017	2.258e-23	BS	intron_variant	PCSK1	3.1e-07	-0.21	Artery - Aorta	FALSE
rs12332295	-0.017	2.258e-23	BS	intron_variant	PCSK1	1.7e-10	-0.31	Heart - Atrial Appendage	FALSE
rs12332295	-0.017	2.258e-23	BS	intron_variant	PCSK1	1.8e-10	-0.2	Adipose - Subcutaneous	FALSE
rs12332295	-0.017	2.258e-23	BS	intron_variant	PCSK1	8.1e-06	-0.15	Artery - Tibial	FALSE
rs13159579	0.059	3.009e-13	PI	intron_variant	PCSK1	2.6e-05	-0.19	Artery - Aorta	TRUE
rs13159579	0.059	3.009e-13	PI	intron_variant	PCSK1	5,00E-11	-0.33	Heart - Atrial Appendage	TRUE
rs13159579	0.059	3.009e-13	PI	intron_variant	PCSK1	2.3e-07	-0.16	Adipose - Subcutaneous	TRUE
rs13159579	0.059	3.009e-13	PI	intron_variant	PCSK1	9.8e-05	-0.13	Artery - Tibial	TRUE
rs13159579	-0.0167	1.577e-20	BS	intron_variant	PCSK1	2.6e-05	-0.19	Artery - Aorta	TRUE
rs13159579	-0.0167	1.577e-20	BS	intron_variant	PCSK1	5,00E-11	-0.33	Heart - Atrial Appendage	TRUE
rs13159579	-0.0167	1.577e-20	BS	intron_variant	PCSK1	2.3e-07	-0.16	Adipose - Subcutaneous	TRUE
rs13159579	-0.0167	1.577e-20	BS	intron_variant	PCSK1	9.8e-05	-0.13	Artery - Tibial	TRUE
rs13168460	-0.0173	3.443e-23	BS	intron_variant	PCSK1	3.8e-12	-0.35	Heart - Atrial Appendage	FALSE
rs13168460	-0.0173	3.443e-23	BS	intron_variant	PCSK1	1.2e-05	-0.19	Artery - Aorta	FALSE
rs13168460	-0.0173	3.443e-23	BS	intron_variant	PCSK1	9.1e-09	-0.19	Adipose - Subcutaneous	FALSE
rs13168460	-0.0173	3.443e-23	BS	intron_variant	PCSK1	6.1e-06	-0.16	Artery - Tibial	FALSE
rs13169290	-0.0171	7.7e-23	BS	intron_variant	PCSK1	1.4e-05	-0.19	Artery - Aorta	FALSE
rs13169290	-0.0171	7.7e-23	BS	intron_variant	PCSK1	4.3e-12	-0.35	Heart - Atrial Appendage	FALSE
rs13169290	-0.0171	7.7e-23	BS	intron_variant	PCSK1	9.5e-09	-0.19	Adipose - Subcutaneous	FALSE
rs13169290	-0.0171	7.7e-23	BS	intron_variant	PCSK1	4.9e-06	-0.16	Artery - Tibial	FALSE
rs1353797	0.046	6.426e-10	PI	intron_variant	PCSK1	1.5e-07	-0.24	Heart - Atrial Appendage	FALSE
rs144489757	-0.0168	9.575e-23	BS	intron_variant	PCSK1	8.5e-08	-0.23	Artery - Aorta	FALSE
rs144489757	-0.0168	9.575e-23	BS	intron_variant	PCSK1	1.9e-12	-0.35	Heart - Atrial Appendage	FALSE
rs144489757	-0.0168	9.575e-23	BS	intron_variant	PCSK1	2,00E-06	-0.16	Artery - Tibial	FALSE
rs144489757	-0.0168	9.575e-23	BS	intron_variant	PCSK1	1.6e-10	-0.21	Adipose - Subcutaneous	FALSE
rs145266997	-0.0177	5.299e-09	BS	intron_variant	PCSK1	1.9e-05	-0.36	Heart - Atrial Appendage	FALSE
rs145266997	-0.0177	5.299e-09	BS	intron_variant	PCSK1	1,00E-04	-0.23	Adipose - Subcutaneous	FALSE
rs155995	-0.045	2.508e-09	PI	intron_variant	PCSK1	1,00E-05	0.21	Heart - Atrial Appendage	FALSE
rs17085655	-0.0171	1.926e-23	BS	intron_variant	PCSK1	1.7e-10	-0.31	Heart - Atrial Appendage	FALSE
rs17085655	-0.0171	1.926e-23	BS	intron_variant	PCSK1	8.1e-06	-0.15	Artery - Tibial	FALSE
rs17085655	-0.0171	1.926e-23	BS	intron_variant	PCSK1	3.1e-07	-0.21	Artery - Aorta	FALSE
rs17085655	-0.0171	1.926e-23	BS	intron_variant	PCSK1	1.8e-10	-0.2	Adipose - Subcutaneous	FALSE
rs17085658	-0.017	2.395e-23	BS	intron_variant	PCSK1	2.2e-07	-0.22	Artery - Aorta	FALSE
rs17085658	-0.017	2.395e-23	BS	intron_variant	PCSK1	8.5e-06	-0.15	Artery - Tibial	FALSE
rs17085658	-0.017	2.395e-23	BS	intron_variant	PCSK1	1.8e-10	-0.2	Adipose - Subcutaneous	FALSE
rs17085658	-0.017	2.395e-23	BS	intron_variant	PCSK1	2,00E-10	-0.31	Heart - Atrial Appendage	FALSE
rs17085665	-0.017	2.873e-23	BS	intron_variant	PCSK1	1.6e-06	-0.16	Artery - Tibial	FALSE
rs17085665	-0.017	2.873e-23	BS	intron_variant	PCSK1	1.5e-11	-0.32	Heart - Atrial Appendage	FALSE
rs17085665	-0.017	2.873e-23	BS	intron_variant	PCSK1	4.7e-07	-0.21	Artery - Aorta	FALSE
rs17085665	-0.017	2.873e-23	BS	intron_variant	PCSK1	2.2e-09	-0.19	Adipose - Subcutaneous	FALSE
rs17085675	-0.0169	5.912e-23	BS	3_prime_UTR_variant	PCSK1	5.3e-15	-0.37	Heart - Atrial Appendage	TRUE
rs17085675	-0.0169	5.912e-23	BS	3_prime_UTR_variant	PCSK1	1.7e-09	-0.19	Adipose - Subcutaneous	TRUE
rs17085675	-0.0169	5.912e-23	BS	3_prime_UTR_variant	PCSK1	4.4e-09	-0.24	Artery - Aorta	TRUE
rs17085675	-0.0169	5.912e-23	BS	3_prime_UTR_variant	PCSK1	9.5e-07	-0.16	Artery - Tibial	TRUE
rs1820176	-0.0171	2.079e-23	BS	intron_variant	PCSK1	6.9e-06	-0.18	Artery - Aorta	FALSE
rs1820176	-0.0171	2.079e-23	BS	intron_variant	PCSK1	3.5e-10	-0.19	Adipose - Subcutaneous	FALSE
rs1820176	-0.0171	2.079e-23	BS	intron_variant	PCSK1	3.5e-10	-0.3	Heart - Atrial Appendage	FALSE
rs1820176	-0.0171	2.079e-23	BS	intron_variant	PCSK1	1,00E-05	-0.15	Artery - Tibial	FALSE
rs1820177	-0.017	3.082e-23	BS	intron_variant	PCSK1	2.9e-10	-0.19	Adipose - Subcutaneous	FALSE
rs1820177	-0.017	3.082e-23	BS	intron_variant	PCSK1	3.3e-06	-0.15	Artery - Tibial	FALSE
rs1820177	-0.017	3.082e-23	BS	intron_variant	PCSK1	2.1e-10	-0.3	Heart - Atrial Appendage	FALSE
rs1820177	-0.017	3.082e-23	BS	intron_variant	PCSK1	1.4e-05	-0.18	Artery - Aorta	FALSE
rs1874929	0.047	2.086e-10	PI	intron_variant	PCSK1	1.8e-07	-0.25	Heart - Atrial Appendage	FALSE
rs193069188	-0.0175	1.62e-23	BS	intron_variant	PCSK1	7.7e-08	-0.17	Adipose - Subcutaneous	FALSE
rs193069188	-0.0175	1.62e-23	BS	intron_variant	PCSK1	2.6e-05	-0.14	Artery - Tibial	FALSE
rs193069188	-0.0175	1.62e-23	BS	intron_variant	PCSK1	5.5e-06	-0.19	Artery - Aorta	FALSE
rs193069188	-0.0175	1.62e-23	BS	intron_variant	PCSK1	4.4e-15	-0.39	Heart - Atrial Appendage	FALSE
rs271939	0.048	1.345e-10	PI	intron_variant	PCSK1	1.2e-07	-0.25	Heart - Atrial Appendage	FALSE
rs2882298	-0.017	2.691e-23	BS	3_prime_UTR_variant	PCSK1	4.4e-09	-0.24	Artery - Aorta	TRUE
rs2882298	-0.017	2.691e-23	BS	3_prime_UTR_variant	PCSK1	5.2e-16	-0.38	Heart - Atrial Appendage	TRUE
rs2882298	-0.017	2.691e-23	BS	3_prime_UTR_variant	PCSK1	3.1e-06	-0.15	Artery - Tibial	TRUE
rs2882298	-0.017	2.691e-23	BS	3_prime_UTR_variant	PCSK1	4.3e-09	-0.18	Adipose - Subcutaneous	TRUE
rs34874677	-0.0173	3.613e-23	BS	intron_variant	PCSK1	1.4e-06	-0.16	Artery - Tibial	FALSE
rs34874677	-0.0173	3.613e-23	BS	intron_variant	PCSK1	1.1e-10	-0.3	Heart - Atrial Appendage	FALSE
rs34874677	-0.0173	3.613e-23	BS	intron_variant	PCSK1	1.9e-10	-0.2	Adipose - Subcutaneous	FALSE
rs34874677	-0.0173	3.613e-23	BS	intron_variant	PCSK1	1.2e-06	-0.2	Artery - Aorta	FALSE
rs35247507	-0.0166	4.15e-22	BS	intron_variant	PCSK1	1.9e-06	-0.16	Artery - Tibial	FALSE
rs35247507	-0.0166	4.15e-22	BS	intron_variant	PCSK1	2.5e-16	-0.39	Heart - Atrial Appendage	FALSE
rs35247507	-0.0166	4.15e-22	BS	intron_variant	PCSK1	2.7e-08	-0.23	Artery - Aorta	FALSE
rs35247507	-0.0166	4.15e-22	BS	intron_variant	PCSK1	4.3e-09	-0.18	Adipose - Subcutaneous	FALSE
rs35648612	-0.017	3.309e-23	BS	intron_variant	PCSK1	2.5e-08	-0.23	Artery - Aorta	FALSE
rs35648612	-0.017	3.309e-23	BS	intron_variant	PCSK1	4,00E-16	-0.38	Heart - Atrial Appendage	FALSE
rs35648612	-0.017	3.309e-23	BS	intron_variant	PCSK1	3.2e-06	-0.15	Artery - Tibial	FALSE
rs35648612	-0.017	3.309e-23	BS	intron_variant	PCSK1	7.4e-09	-0.18	Adipose - Subcutaneous	FALSE
rs36115340	-0.0168	2.233e-22	BS	intron_variant	PCSK1	1.5e-16	-0.39	Heart - Atrial Appendage	TRUE
rs36115340	-0.0168	2.233e-22	BS	intron_variant	PCSK1	3.7e-09	-0.19	Adipose - Subcutaneous	TRUE
rs36115340	-0.0168	2.233e-22	BS	intron_variant	PCSK1	0.00012	-0.13	Adipose - Visceral (Omentum)	TRUE
rs36115340	-0.0168	2.233e-22	BS	intron_variant	PCSK1	2.6e-05	-0.14	Artery - Tibial	TRUE
rs36115340	-0.0168	2.233e-22	BS	intron_variant	PCSK1	1.2e-08	-0.24	Artery - Aorta	TRUE
rs369734422	-0.0186	5.397e-27	BS	intron_variant	PCSK1	4.4e-15	-0.39	Heart - Atrial Appendage	FALSE
rs369734422	-0.0186	5.397e-27	BS	intron_variant	PCSK1	5.5e-06	-0.19	Artery - Aorta	FALSE
rs369734422	-0.0186	5.397e-27	BS	intron_variant	PCSK1	7.7e-08	-0.17	Adipose - Subcutaneous	FALSE
rs369734422	-0.0186	5.397e-27	BS	intron_variant	PCSK1	2.6e-05	-0.14	Artery - Tibial	FALSE
rs373259621	-0.0186	5.397e-27	BS	intron_variant	PCSK1	2.6e-05	-0.14	Artery - Tibial	FALSE
rs373259621	-0.0186	5.397e-27	BS	intron_variant	PCSK1	5.5e-06	-0.19	Artery - Aorta	FALSE
rs373259621	-0.0186	5.397e-27	BS	intron_variant	PCSK1	7.7e-08	-0.17	Adipose - Subcutaneous	FALSE
rs373259621	-0.0186	5.397e-27	BS	intron_variant	PCSK1	4.4e-15	-0.39	Heart - Atrial Appendage	FALSE
rs3811951	-0.0154	9.89e-19	BS	intron_variant	PCSK1	5.7e-11	-0.33	Heart - Atrial Appendage	FALSE
rs3811951	-0.0154	9.89e-19	BS	intron_variant	PCSK1	2.9e-05	-0.14	Artery - Tibial	FALSE
rs3811951	-0.0154	9.89e-19	BS	intron_variant	PCSK1	3.8e-05	-0.18	Artery - Aorta	FALSE
rs3811951	-0.0154	9.89e-19	BS	intron_variant	PCSK1	1.6e-07	-0.17	Adipose - Subcutaneous	FALSE
rs436321	-0.045	2.06e-09	PI	intron_variant	PCSK1	9.9e-06	0.21	Heart - Atrial Appendage	FALSE
rs456709	-0.043	9.287e-09	PI	intron_variant	PCSK1	9.9e-06	0.21	Heart - Atrial Appendage	FALSE
rs59139497	-0.017	2.872e-23	BS	intron_variant	PCSK1	1.6e-06	-0.16	Artery - Tibial	FALSE
rs59139497	-0.017	2.872e-23	BS	intron_variant	PCSK1	3.3e-09	-0.19	Adipose - Subcutaneous	FALSE
rs59139497	-0.017	2.872e-23	BS	intron_variant	PCSK1	2.1e-16	-0.39	Heart - Atrial Appendage	FALSE
rs59139497	-0.017	2.872e-23	BS	intron_variant	PCSK1	8.8e-08	-0.22	Artery - Aorta	FALSE
rs6863434	0.055	5.298e-13	PI	intron_variant	PCSK1	2.9e-10	-0.19	Adipose - Subcutaneous	TRUE
rs6863434	0.055	5.298e-13	PI	intron_variant	PCSK1	1.4e-10	-0.3	Heart - Atrial Appendage	TRUE
rs6863434	0.055	5.298e-13	PI	intron_variant	PCSK1	1.1e-05	-0.18	Artery - Aorta	TRUE
rs6863434	0.055	5.298e-13	PI	intron_variant	PCSK1	3.3e-06	-0.15	Artery - Tibial	TRUE
rs6863434	-0.0169	4.923e-23	BS	intron_variant	PCSK1	2.9e-10	-0.19	Adipose - Subcutaneous	TRUE
rs6863434	-0.0169	4.923e-23	BS	intron_variant	PCSK1	1.4e-10	-0.3	Heart - Atrial Appendage	TRUE
rs6863434	-0.0169	4.923e-23	BS	intron_variant	PCSK1	1.1e-05	-0.18	Artery - Aorta	TRUE
rs6863434	-0.0169	4.923e-23	BS	intron_variant	PCSK1	3.3e-06	-0.15	Artery - Tibial	TRUE
rs6863434	-0.02	5.259e-09	FGadjBMI	intron_variant	PCSK1	2.9e-10	-0.19	Adipose - Subcutaneous	TRUE
rs6863434	-0.02	5.259e-09	FGadjBMI	intron_variant	PCSK1	1.4e-10	-0.3	Heart - Atrial Appendage	TRUE
rs6863434	-0.02	5.259e-09	FGadjBMI	intron_variant	PCSK1	1.1e-05	-0.18	Artery - Aorta	TRUE
rs6863434	-0.02	5.259e-09	FGadjBMI	intron_variant	PCSK1	3.3e-06	-0.15	Artery - Tibial	TRUE
rs6869253	-0.0169	4.367e-23	BS	intron_variant	PCSK1	7.8e-06	-0.15	Artery - Tibial	FALSE
rs6869253	-0.0169	4.367e-23	BS	intron_variant	PCSK1	1.7e-10	-0.31	Heart - Atrial Appendage	FALSE
rs6869253	-0.0169	4.367e-23	BS	intron_variant	PCSK1	2.8e-10	-0.2	Adipose - Subcutaneous	FALSE
rs6869253	-0.0169	4.367e-23	BS	intron_variant	PCSK1	1.4e-06	-0.2	Artery - Aorta	FALSE
rs6869570	-0.0166	5.487e-20	BS	intron_variant	PCSK1	6.1e-05	-0.14	Artery - Tibial	FALSE
rs6869570	-0.0166	5.487e-20	BS	intron_variant	PCSK1	3.9e-08	-0.18	Adipose - Subcutaneous	FALSE
rs6869570	-0.0166	5.487e-20	BS	intron_variant	PCSK1	9.6e-12	-0.34	Heart - Atrial Appendage	FALSE

rs6869570	-0.0166	5.487e-20	BS	intron_variant	PCSK1	4,00E-05	-0.18	Artery - Aorta	FALSE
rs6876986	-0.017	4.233e-23	BS	intron_variant	PCSK1	2.5e-10	-0.2	Adipose - Subcutaneous	FALSE
rs6876986	-0.017	4.233e-23	BS	intron_variant	PCSK1	2.8e-10	-0.3	Heart - Atrial Appendage	FALSE
rs6876986	-0.017	4.233e-23	BS	intron_variant	PCSK1	1.4e-06	-0.2	Artery - Aorta	FALSE
rs6876986	-0.017	4.233e-23	BS	intron_variant	PCSK1	1.6e-05	-0.14	Artery - Tibial	FALSE
rs6889272	-0.0165	8.313e-20	BS	intron_variant	PCSK1	9.5e-05	-0.13	Artery - Tibial	FALSE
rs6889272	-0.0165	8.313e-20	BS	intron_variant	PCSK1	2.5e-07	-0.17	Adipose - Subcutaneous	FALSE
rs6889272	-0.0165	8.313e-20	BS	intron_variant	PCSK1	9.4e-06	-0.19	Artery - Aorta	FALSE
rs6889272	-0.0165	8.313e-20	BS	intron_variant	PCSK1	7.6e-12	-0.34	Heart - Atrial Appendage	FALSE
rs6890994	-0.017	3.241e-23	BS	intron_variant	PCSK1	8.8e-08	-0.22	Artery - Aorta	FALSE
rs6890994	-0.017	3.241e-23	BS	intron_variant	PCSK1	2.1e-16	-0.39	Heart - Atrial Appendage	FALSE
rs6890994	-0.017	3.241e-23	BS	intron_variant	PCSK1	1.6e-06	-0.16	Artery - Tibial	FALSE
rs6890994	-0.017	3.241e-23	BS	intron_variant	PCSK1	3.3e-09	-0.19	Adipose - Subcutaneous	FALSE
rs7700417	-0.0171	1.97e-23	BS	intron_variant	PCSK1	2.1e-10	-0.31	Heart - Atrial Appendage	FALSE
rs7700417	-0.0171	1.97e-23	BS	intron_variant	PCSK1	8.3e-06	-0.15	Artery - Tibial	FALSE
rs7700417	-0.0171	1.97e-23	BS	intron_variant	PCSK1	1,00E-06	-0.2	Artery - Aorta	FALSE
rs7700417	-0.0171	1.97e-23	BS	intron_variant	PCSK1	2.8e-10	-0.2	Adipose - Subcutaneous	FALSE
rs7713317	-0.017	3.097e-23	BS	intron_variant	PCSK1	1.1e-05	-0.15	Artery - Tibial	TRUE
rs7713317	-0.017	3.097e-23	BS	intron_variant	PCSK1	1.2e-08	-0.24	Artery - Aorta	TRUE
rs7713317	-0.017	3.097e-23	BS	intron_variant	PCSK1	5.7e-17	-0.4	Heart - Atrial Appendage	TRUE
rs7713317	-0.017	3.097e-23	BS	intron_variant	PCSK1	2.8e-09	-0.19	Adipose - Subcutaneous	TRUE
rs7713317	-0.0194	1.252e-17	FG	intron_variant	PCSK1	1.1e-05	-0.15	Artery - Tibial	TRUE
rs7713317	-0.0194	1.252e-17	FG	intron_variant	PCSK1	1.2e-08	-0.24	Artery - Aorta	TRUE
rs7713317	-0.0194	1.252e-17	FG	intron_variant	PCSK1	5.7e-17	-0.4	Heart - Atrial Appendage	TRUE
rs7713317	-0.0194	1.252e-17	FG	intron_variant	PCSK1	2.8e-09	-0.19	Adipose - Subcutaneous	TRUE
rs7722200	-0.017	2.214e-23	BS	intron_variant	PCSK1	1.7e-10	-0.31	Heart - Atrial Appendage	FALSE
rs7722200	-0.017	2.214e-23	BS	intron_variant	PCSK1	7.8e-06	-0.15	Artery - Tibial	FALSE
rs7722200	-0.017	2.214e-23	BS	intron_variant	PCSK1	1.4e-06	-0.2	Artery - Aorta	FALSE
rs7722200	-0.017	2.214e-23	BS	intron_variant	PCSK1	2.8e-10	-0.2	Adipose - Subcutaneous	FALSE
rs9285019	-0.017	2.371e-23	BS	intron_variant	PCSK1	1.3e-05	-0.14	Artery - Tibial	FALSE
rs9285019	-0.017	2.371e-23	BS	intron_variant	PCSK1	1,00E-09	-0.19	Adipose - Subcutaneous	FALSE
rs9285019	-0.017	2.371e-23	BS	intron_variant	PCSK1	9.8e-10	-0.29	Heart - Atrial Appendage	FALSE
rs9285019	-0.017	2.371e-23	BS	intron_variant	PCSK1	1.5e-06	-0.2	Artery - Aorta	FALSE
NA	NA	NA	NA	NA	NA	NA	NA	NA	FALSE
rs11372849	0.0617	2.892e-09	HYPERTENSION	intron_variant	FURIN	1.2e-07	0.099	Artery - Tibial	FALSE
rs11372849	0.0617	2.892e-09	HYPERTENSION	intron_variant	FURIN	3.4e-06	0.094	Artery - Aorta	FALSE
rs11372849	0.062	7.62e-13	CAD	intron_variant	FURIN	1.2e-07	0.099	Artery - Tibial	FALSE
rs11372849	0.062	7.62e-13	CAD	intron_variant	FURIN	3.4e-06	0.094	Artery - Aorta	FALSE
rs11539637	-0.0641	3.151e-10	HYPERTENSION	synonymous_variant	FURIN	5,00E-05	-0.081	Artery - Aorta	TRUE
rs11539637	-0.0641	3.151e-10	HYPERTENSION	synonymous_variant	FURIN	1.4e-05	-0.08	Artery - Tibial	TRUE
rs11539637	-0.0599	5.584e-27	CAD	synonymous_variant	FURIN	5,00E-05	-0.081	Artery - Aorta	TRUE
rs11539637	-0.0599	5.584e-27	CAD	synonymous_variant	FURIN	1.4e-05	-0.08	Artery - Tibial	TRUE
rs11539637	-0.0299	1.344e-50	DBP	synonymous_variant	FURIN	5,00E-05	-0.081	Artery - Aorta	TRUE
rs11539637	-0.0299	1.344e-50	DBP	synonymous_variant	FURIN	1.4e-05	-0.08	Artery - Tibial	TRUE
rs11539637	-0.0308	3.784e-49	SBP	synonymous_variant	FURIN	5,00E-05	-0.081	Artery - Aorta	TRUE
rs11539637	-0.0308	3.784e-49	SBP	synonymous_variant	FURIN	1.4e-05	-0.08	Artery - Tibial	TRUE
rs11638635	-0.0552	3.86e-23	CAD	non_coding_transcript_exon_variant	FURIN	2.7e-05	-0.084	Artery - Aorta	FALSE
rs11638635	-0.0271	1.104e-41	SBP	non_coding_transcript_exon_variant	FURIN	2.7e-05	-0.084	Artery - Aorta	FALSE
rs11638635	-0.0271	2.525e-39	DBP	non_coding_transcript_exon_variant	FURIN	2.7e-05	-0.084	Artery - Aorta	FALSE
rs12909124	-0.0271	2.5e-39	DBP	non_coding_transcript_exon_variant	FURIN	2.1e-05	-0.085	Artery - Aorta	TRUE
rs12909124	-0.0271	1.298e-41	SBP	non_coding_transcript_exon_variant	FURIN	2.1e-05	-0.085	Artery - Aorta	TRUE
rs12909124	-0.0551	5.959e-22	CAD	non_coding_transcript_exon_variant	FURIN	2.1e-05	-0.085	Artery - Aorta	TRUE
rs12917588	-0.0269	1.92e-09	DBP	intergenic_variant	FURIN	4.9e-05	-0.16	Artery - Aorta	TRUE
rs12917588	-0.0264	1.108e-09	SBP	intergenic_variant	FURIN	4.9e-05	-0.16	Artery - Aorta	TRUE
rs1573643	0.0349	6.096e-53	DBP	intron_variant	FURIN	5,00E-06	0.091	Artery - Aorta	TRUE
rs1573643	0.0349	6.096e-53	DBP	intron_variant	FURIN	0.00013	0.074	Artery - Tibial	TRUE
rs1573643	0.0346	1.042e-54	SBP	intron_variant	FURIN	5,00E-06	0.091	Artery - Aorta	TRUE
rs1573643	0.0346	1.042e-54	SBP	intron_variant	FURIN	0.00013	0.074	Artery - Tibial	TRUE
rs1573643	0.0616	1.363e-25	CAD	intron_variant	FURIN	5,00E-06	0.091	Artery - Aorta	TRUE
rs1573643	0.0616	1.363e-25	CAD	intron_variant	FURIN	0.00013	0.074	Artery - Tibial	TRUE
rs1573643	0.085	3.049e-14	HYPERTENSION	intron_variant	FURIN	5,00E-06	0.091	Artery - Aorta	TRUE
rs1573643	0.085	3.049e-14	HYPERTENSION	intron_variant	FURIN	0.00013	0.074	Artery - Tibial	TRUE
rs17514846	0.0293	9.139e-55	DBP	intron_variant	FURIN	4.6e-06	0.087	Artery - Tibial	FALSE
rs17514846	0.0293	9.139e-55	DBP	intron_variant	FURIN	4.9e-05	0.083	Artery - Aorta	FALSE
rs17514846	0.0305	2.452e-45	SBP	intron_variant	FURIN	4.6e-06	0.087	Artery - Tibial	FALSE
rs17514846	0.0305	2.452e-45	SBP	intron_variant	FURIN	4.9e-05	0.083	Artery - Aorta	FALSE
rs17514846	0.0165	1.622e-09	HYPERTENSION	intron_variant	FURIN	4.6e-06	0.087	Artery - Tibial	FALSE
rs17514846	0.0165	1.622e-09	HYPERTENSION	intron_variant	FURIN	4.9e-05	0.083	Artery - Aorta	FALSE
rs17514846	0.0555	2.326e-36	CAD	intron_variant	FURIN	4.6e-06	0.087	Artery - Tibial	FALSE
rs17514846	0.0555	2.326e-36	CAD	intron_variant	FURIN	4.9e-05	0.083	Artery - Aorta	FALSE
rs1894401	-0.0305	3.086e-53	SBP	intron_variant	FURIN	6.9e-06	-0.089	Artery - Aorta	TRUE
rs1894401	-0.0305	3.086e-53	SBP	intron_variant	FURIN	9.8e-06	-0.081	Artery - Tibial	TRUE
rs1894401	-0.0293	3.3e-46	DBP	intron_variant	FURIN	6.9e-06	-0.089	Artery - Aorta	TRUE
rs1894401	-0.0293	3.3e-46	DBP	intron_variant	FURIN	9.8e-06	-0.081	Artery - Tibial	TRUE
rs1894401	-0.0648	2.223e-10	HYPERTENSION	intron_variant	FURIN	6.9e-06	-0.089	Artery - Aorta	TRUE
rs1894401	-0.0648	2.223e-10	HYPERTENSION	intron_variant	FURIN	9.8e-06	-0.081	Artery - Tibial	TRUE
rs1894401	-0.059	4.372e-26	CAD	intron_variant	FURIN	6.9e-06	-0.089	Artery - Aorta	TRUE
rs1894401	-0.059	4.372e-26	CAD	intron_variant	FURIN	9.8e-06	-0.081	Artery - Tibial	TRUE
rs200266974	0.0549	7.47e-10	CAD	intron_variant	FURIN	0.00011	0.071	Artery - Tibial	FALSE
rs2071382	-0.031	7.675e-53	SBP	intron_variant	FURIN	3.4e-05	-0.076	Artery - Tibial	FALSE
rs2071382	-0.0608	1.33e-27	CAD	intron_variant	FURIN	3.4e-05	-0.076	Artery - Tibial	FALSE
rs2071382	-0.064	3.395e-10	HYPERTENSION	intron_variant	FURIN	3.4e-05	-0.076	Artery - Tibial	FALSE
rs2071382	-0.0299	6.65e-48	DBP	intron_variant	FURIN	3.4e-05	-0.076	Artery - Tibial	FALSE
rs2071384	-0.0295	7.711e-44	DBP	intron_variant	FURIN	7.6e-06	-0.09	Artery - Aorta	FALSE
rs2071384	-0.03	5.484e-45	SBP	intron_variant	FURIN	7.6e-06	-0.09	Artery - Aorta	FALSE
rs2071384	-0.0601	3.101e-25	CAD	intron_variant	FURIN	7.6e-06	-0.09	Artery - Aorta	FALSE
rs2071410	0.0354	4.43e-52	DBP	intron_variant	FURIN	5.9e-05	0.081	Artery - Tibial	TRUE
rs2071410	0.0354	4.43e-52	DBP	intron_variant	FURIN	3,00E-05	0.088	Artery - Aorta	TRUE
rs2071410	0.0626	5.522e-28	CAD	intron_variant	FURIN	5.9e-05	0.081	Artery - Tibial	TRUE
rs2071410	0.0626	5.522e-28	CAD	intron_variant	FURIN	3,00E-05	0.088	Artery - Aorta	TRUE
rs2071410	0.0352	2.255e-49	SBP	intron_variant	FURIN	5.9e-05	0.081	Artery - Tibial	TRUE
rs2071410	0.0352	2.255e-49	SBP	intron_variant	FURIN	3,00E-05	0.088	Artery - Aorta	TRUE
rs2071410	0.0824	2.582e-13	HYPERTENSION	intron_variant	FURIN	5.9e-05	0.081	Artery - Tibial	TRUE
rs2071410	0.0824	2.582e-13	HYPERTENSION	intron_variant	FURIN	3,00E-05	0.088	Artery - Aorta	TRUE
rs2521501	0.0359	2.198e-57	SBP	intron_variant	FURIN	3.1e-05	0.086	Artery - Aorta	FALSE
rs2521501	0.0662	7.807e-30	CAD	intron_variant	FURIN	3.1e-05	0.086	Artery - Aorta	FALSE
rs2521501	0.0361	1.602e-55	DBP	intron_variant	FURIN	3.1e-05	0.086	Artery - Aorta	FALSE
rs2521501	0.0829	2.172e-13	HYPERTENSION	intron_variant	FURIN	3.1e-05	0.086	Artery - Aorta	FALSE
rs4533264	-0.0585	3.219e-24	CAD	intron_variant	FURIN	4.3e-06	-0.091	Artery - Aorta	FALSE
rs4533264	-0.0292	1.502e-43	DBP	intron_variant	FURIN	4.3e-06	-0.091	Artery - Aorta	FALSE
rs4533264	-0.0289	4.304e-45	SBP	intron_variant	FURIN	4.3e-06	-0.091	Artery - Aorta	FALSE
rs4548831	-0.0289	2.671e-45	SBP	intron_variant	FURIN	4.3e-06	-0.091	Artery - Aorta	FALSE
rs4548831	-0.0585	2.905e-24	CAD	intron_variant	FURIN	4.3e-06	-0.091	Artery - Aorta	FALSE
rs4548831	-0.0293	1.231e-43	DBP	intron_variant	FURIN	4.3e-06	-0.091	Artery - Aorta	FALSE
rs4702	0.0453	2.674e-18	CAD	3_prime_UTR_variant	FURIN	1.8e-09	0.11	Artery - Tibial	TRUE
rs4702	0.024	8.166e-28	SBP	3_prime_UTR_variant	FURIN	1.8e-09	0.11	Artery - Tibial	TRUE
rs4702	0.0208	4.24e-22	DBP	3_prime_UTR_variant	FURIN	1.8e-09	0.11	Artery - Tibial	TRUE
rs4932179	-0.029	9.167e-47	SBP	intron_variant	FURIN	4,00E-05	-0.083	Artery - Aorta	FALSE
rs4932179	-0.06	2.962e-25	CAD	intron_variant	FURIN	4,00E-05	-0.083	Artery - Aorta	FALSE
rs4932179	-0.0289	2.951e-43	DBP	intron_variant	FURIN	4,00E-05	-0.083	Artery - Aorta	FALSE
rs4932370	0.0317	5.163e-46	DBP	TF_binding_site_variant	FURIN	8.9e-05	0.078	Artery - Tibial	FALSE
rs4932370	0.0549	6.634e-30	CAD	TF_binding_site_variant	FURIN	8.9e-05	0.078	Artery - Tibial	FALSE
rs4932370	0.0697	3.597e-10	HYPERTENSION	TF_binding_site_variant	FURIN	8.9e-05	0.078	Artery - Tibial	FALSE
rs4932370	0.0316	5.554e-47	SBP	TF_binding_site_variant	FURIN	8.9e-05	0.078	Artery - Tibial	FALSE
rs4932371	0.0632	7.729e-09	HYPERTENSION	TF_binding_site_variant	FURIN	6.9e-05	0.079	Artery - Tibial	TRUE
rs4932371	0.0506	1.368e-26	CAD	TF_binding_site_variant	FURIN	6.9e-05	0.079	Artery - Tibial	TRUE
rs4932371	0.0299	8.691e-47	SBP	TF_binding_site_variant	FURIN	6.9e-05	0.079	Artery - Tibial	TRUE
rs4932371	0.0291	2.017e-45	DBP	TF_binding_site_variant	FURIN	6.9e-05	0.079	Artery - Tibial	TRUE
rs4932373	0.0351	2.193e-56	SBP	intron_variant	FURIN	7.1e-05	0.085	Artery - Aorta	FALSE
rs4932373	0.0652	1.056e-27	CAD	intron_variant	FURIN	7.1e-05	0.085	Artery - Aorta	FALSE
rs4932373	0.0356	3.534e-55	DBP	intron_variant	FURIN	7.1e-05	0.085	Artery - Aorta	FALSE
rs4932373	0.0856	3.53e-14	HYPERTENSION	intron_variant	FURIN	7.1e-05	0.085	Artery - Aorta	FALSE
rs4932374	-0.0286	2.852e-42	DBP	intron_variant	FURIN	4.3e-06	-0.091	Artery - Aorta	FALSE
rs4932374	-0.0286	5.922e-46	SBP	intron_variant	FURIN	4.3e-06	-0.091	Artery - Aorta	FALSE
rs4932374	-0.0585	2.376e-24	CAD	intron_variant	FURIN	4.3e-06	-0.091	Artery - Aorta	FALSE
rs4932376	-0.0599	2.217e-25	CAD	intron_variant	FURIN	3.6e-05	-0.084	Artery - Aorta	FALSE
rs4932376	-0.0288	2.169e-44	SBP	intron_variant	FURIN	3.6e-05	-0.084	Artery - Aorta	FALSE
rs4932376	-0.0292	2.293e-43	DBP	intron_variant	FURIN	3.6e-05	-0.084	Artery - Aorta	FALSE
rs57515981	0.0595	8.513e-09	HYPERTENSION	intron_variant	FURIN	0.00011	0.071	Artery - Tibial	FALSE
rs6224	0.0606699999999966	1.64e-12	CAD	intron_variant	FURIN	5.4e-06	0.091	Artery - Aorta	FALSE
rs6224	0.0606699999999966	1.64e-12	CAD	intron_variant	FURIN	2.5e-07	0.095	Artery - Tibial	FALSE
rs6224	0.0606699999999966	1.64e-12	CAD	intron_variant	FURIN	5.4e-06	0.091	Artery - Aorta	FALSE
rs6224	0.0606699999999966	1.64e-12	CAD	intron_variant	FURIN	2.5e-07	0.095	Artery - Tibial	FALSE
rs6226	0.0151	2.885e-10	SBP	synonymous_variant	FURIN	1.6e-09	0.12	Artery - Tibial	TRUE
rs6227	0.0356	3.878e-54	SBP	3_prime_UTR_variant	FURIN	4.1e-05	0.082	Artery - Tibial	TRUE
rs6227	0.0358	2.356e-53	DBP	3_prime_UTR_variant	FURIN	4.1e-05	0.082	Artery - Tibial	TRUE
rs6227	0.083	1.835e-13	HYPERTENSION	3_prime_UTR_variant	FURIN	4.1e-05	0.082	Artery - Tibial	TRUE
rs6227	0.0627	2.463e-28	CAD	3_prime_UTR_variant	FURIN	4.1e-05	0.082	Artery - Tibial	TRUE
rs7166599	0.032	2.101e-46	SBP	upstream_gene_variant	FURIN	7,00E-05	0.083	Artery - Aorta	TRUE
rs7166599	0.0684	7.923e-10	HYPERTENSION	upstream_gene_variant	FURIN	7,00E-05	0.083	Artery - Aorta	TRUE
rs7166599	0.032	3.177e-46	DBP	upstream_gene_variant	FURIN	7,00E-05	0.083	Artery - Aorta	TRUE
rs7166599	0.0578	8.852e-22	CAD	upstream_gene_variant	FURIN	7,00E-05	0.083	Artery - Aorta	TRUE
rs8027450	0.0828	2.151e-13	HYPERTENSION	intron_variant	FURIN	9.1e-05	0.08	Artery - Tibial	FALSE
rs8027450	0.0828	2.151e-13	HYPERTENSION	intron_variant	FURIN	4.1e-05	0.088	Artery - Aorta	FALSE
rs8027450	0.0354	8.291e-55	SBP	intron_variant	FURIN	9.1e-05	0.08	Artery - Tibial	FALSE
rs8027450	0.0354	8.291e-55	SBP	intron_variant	FURIN	4.1e-05	0.088	Artery - Aorta	FALSE
rs8027450	0.0645	2.569e-27	CAD	intron_variant	FURIN	9.1e-05	0.08	Artery - Tibial	FALSE
rs8027450	0.0645	2.569e-27	CAD	intron_variant	FURIN	4.1e-05	0.088	Artery - Aorta	FALSE
rs8027450	0.0359	6.385e-55	DBP	intron_variant	FURIN	9.1e-05	0.08	Artery - Tibial	FALSE
rs8027450	0.0359	6.385e-55	DBP	intron_variant	FURIN	4.1e-05	0.088	Artery - Aorta	FALSE

rs8032315	0.0829	1.966e-13	HYPERTENSION	intron_variant	FURIN	6,00E-05	0.083	Artery - Aorta	FALSE
rs8032315	0.0829	1.966e-13	HYPERTENSION	intron_variant	FURIN	9.3e-05	0.077	Artery - Tibial	FALSE
rs8032315	0.0351	7.685e-54	SBP	intron_variant	FURIN	6,00E-05	0.083	Artery - Aorta	FALSE
rs8032315	0.0351	7.685e-54	SBP	intron_variant	FURIN	9.3e-05	0.077	Artery - Tibial	FALSE
rs8032315	0.0358	1.442e-54	DBP	intron_variant	FURIN	6,00E-05	0.083	Artery - Aorta	FALSE
rs8032315	0.0358	1.442e-54	DBP	intron_variant	FURIN	9.3e-05	0.077	Artery - Tibial	FALSE
rs8032315	0.0635	2.535e-30	CAD	intron_variant	FURIN	6,00E-05	0.083	Artery - Aorta	FALSE
rs8032315	0.0635	2.535e-30	CAD	intron_variant	FURIN	9.3e-05	0.077	Artery - Tibial	FALSE
rs8039305	0.0302	2.399e-50	DBP	intron_variant	FURIN	5.8e-08	0.1	Artery - Tibial	TRUE
rs8039305	0.0302	2.399e-50	DBP	intron_variant	FURIN	3.1e-06	0.093	Artery - Aorta	TRUE
rs8039305	0.0637	3.706e-10	HYPERTENSION	intron_variant	FURIN	5.8e-08	0.1	Artery - Tibial	TRUE
rs8039305	0.0637	3.706e-10	HYPERTENSION	intron_variant	FURIN	3.1e-06	0.093	Artery - Aorta	TRUE
rs8039305	0.0316	1.136e-55	SBP	intron_variant	FURIN	5.8e-08	0.1	Artery - Tibial	TRUE
rs8039305	0.0316	1.136e-55	SBP	intron_variant	FURIN	3.1e-06	0.093	Artery - Aorta	TRUE
rs8039305	0.06	5.199e-27	CAD	intron_variant	FURIN	5.8e-08	0.1	Artery - Tibial	TRUE
rs8039305	0.06	5.199e-27	CAD	intron_variant	FURIN	3.1e-06	0.093	Artery - Aorta	TRUE
NA	NA	NA	NA	NA	NA	NA	NA	NA	FALSE
NA	NA	NA	NA	NA	NA	NA	NA	NA	FALSE
NA	NA	NA	NA	NA	NA	NA	NA	NA	FALSE
rs10750103	-0.044	3.45e-256	TG	intron_variant	PCSK7	1.8e-08	-0.097	Whole Blood	FALSE
rs10750103	-0.044	3.45e-256	TG	intron_variant	PCSK7	1.6e-05	-0.2	Pancreas	FALSE
rs10750103	-0.0158	3.661e-13	LDL	intron_variant	PCSK7	1.8e-08	-0.097	Whole Blood	FALSE
rs10750103	-0.0158	3.661e-13	LDL	intron_variant	PCSK7	1.6e-05	-0.2	Pancreas	FALSE
rs10790175	-0.0261	3.738e-28	TG	non_coding_transcript_exon_variant	PCSK7	3.8e-06	-0.085	Whole Blood	FALSE
rs10790177	-0.0274	1.199e-29	TG	upstream_gene_variant	PCSK7	4.3e-06	-0.084	Whole Blood	FALSE
rs10892080	-0.0447	7.79e-260	TG	intron_variant	PCSK7	1.8e-08	-0.097	Whole Blood	TRUE
rs10892080	-0.0447	7.79e-260	TG	intron_variant	PCSK7	1.6e-05	-0.2	Pancreas	TRUE
rs10892080	-0.016	1.78e-13	LDL	intron_variant	PCSK7	1.8e-08	-0.097	Whole Blood	TRUE
rs10892080	-0.016	1.78e-13	LDL	intron_variant	PCSK7	1.6e-05	-0.2	Pancreas	TRUE
rs10892083	-0.0128	8.977e-09	LDL	intron_variant	PCSK7	7.4e-05	-0.068	Whole Blood	FALSE
rs10892083	-0.0286	9.699e-33	TG	intron_variant	PCSK7	7.4e-05	-0.068	Whole Blood	FALSE
rs111809212	0.0194	2.095e-10	LDL	intron_variant	PCSK7	2.7e-06	0.29	Pancreas	FALSE
rs111809212	0.0395	9.159e-34	TG	intron_variant	PCSK7	2.7e-06	0.29	Pancreas	FALSE
rs11216338	0.0146	8.452e-10	TG	intron_variant	PCSK7	1.6e-05	0.075	Whole Blood	FALSE
rs115787357	0.0456	5.138e-37	TG	intron_variant	PCSK7	6,00E-07	0.35	Pancreas	TRUE
rs115787357	0.0219	3.779e-10	LDL	intron_variant	PCSK7	6,00E-07	0.35	Pancreas	TRUE
rs117279127	-0.0702	1.578e-21	TG	non_coding_transcript_exon_variant	PCSK7	5.5e-05	-0.28	Adipose - Subcutaneous	TRUE
rs117680585	0.0386	2.261e-14	LDL	upstream_gene_variant	PCSK7	6.1e-05	0.42	Pancreas	FALSE
rs117680585	0.0752	1.287e-33	TG	upstream_gene_variant	PCSK7	6.1e-05	0.42	Pancreas	FALSE
rs117933040	0.0389	1.413e-14	LDL	5_prime_UTR_variant	PCSK7	6.1e-05	0.42	Pancreas	TRUE
rs117933040	0.0749	2.954e-38	TG	5_prime_UTR_variant	PCSK7	6.1e-05	0.42	Pancreas	TRUE
rs118155116	-0.0701	1.448e-21	TG	intron_variant	PCSK7	5.5e-05	-0.28	Adipose - Subcutaneous	FALSE
rs1242127	0.0318	7.403e-25	TG	3_prime_UTR_variant	PCSK7	7.1e-06	0.082	Whole Blood	FALSE
rs1242229	-0.0264	1.291e-21	LDL	non_coding_transcript_exon_variant	PCSK7	2.1e-05	-0.11	Whole Blood	FALSE
rs1242229	-0.0894	6.015e-63	TG	non_coding_transcript_exon_variant	PCSK7	2.1e-05	-0.11	Whole Blood	FALSE
rs1242230	-0.0259	1.665e-21	LDL	non_coding_transcript_exon_variant	PCSK7	2.1e-05	-0.11	Whole Blood	FALSE
rs143668212	-0.0703	1.176e-21	TG	intron_variant	PCSK7	5.5e-05	-0.28	Adipose - Subcutaneous	FALSE
rs171052	0.025	1.822e-24	TG	non_coding_transcript_exon_variant	PCSK7	1.4e-06	0.088	Whole Blood	FALSE
rs17120523	-0.0411	1.623e-10	CHOL	5_prime_UTR_variant	PCSK7	9.4e-05	0.21	Adipose - Subcutaneous	TRUE
rs17120523	-0.0295	3.155e-12	LDL	5_prime_UTR_variant	PCSK7	9.4e-05	0.21	Adipose - Subcutaneous	TRUE
rs17120523	-0.072	9.468e-52	TG	5_prime_UTR_variant	PCSK7	9.4e-05	0.21	Adipose - Subcutaneous	TRUE
rs1784042	-0.0275	1.783e-30	TG	intron_variant	PCSK7	4.5e-05	-0.07	Whole Blood	FALSE
rs1784042	-0.0129	7.194e-09	LDL	intron_variant	PCSK7	4.5e-05	-0.07	Whole Blood	FALSE
rs1794116	-0.0276	5.745e-29	TG	intron_variant	PCSK7	1.6e-06	-0.088	Whole Blood	FALSE
rs186782888	0.1964	1,00E-300	TG	non_coding_transcript_exon_variant	PCSK7	3,00E-05	0.68	Pancreas	FALSE
rs186782888	0.0664	2.201e-10	LDL	non_coding_transcript_exon_variant	PCSK7	3,00E-05	0.68	Pancreas	FALSE
rs2075547	0.0408	7.765e-35	TG	upstream_gene_variant	PCSK7	1.2e-06	0.34	Pancreas	TRUE
rs2075547	0.0211	1.18e-11	LDL	upstream_gene_variant	PCSK7	1.2e-06	0.34	Pancreas	TRUE
rs2127905	-0.0962	1.536e-40	TG	intron_variant	PCSK7	3.7e-05	-0.11	Whole Blood	FALSE
rs2127905	-0.0263	5.371e-23	LDL	intron_variant	PCSK7	3.7e-05	-0.11	Whole Blood	FALSE
rs2306473	0.0201	1.283e-10	LDL	synonymous_variant	PCSK7	3.7e-06	0.28	Pancreas	TRUE
rs2306473	0.0382	5.845e-09	TGtoHDL	synonymous_variant	PCSK7	3.7e-06	0.28	Pancreas	TRUE
rs2306473	0.0418	2.194e-39	TG	synonymous_variant	PCSK7	3.7e-06	0.28	Pancreas	TRUE
rs236910	0.0246	3.677e-24	TG	non_coding_transcript_exon_variant	PCSK7	1.2e-06	0.089	Whole Blood	FALSE
rs236915	0.0402	6.84e-243	TG	non_coding_transcript_exon_variant	PCSK7	1.4e-05	0.21	Pancreas	FALSE
rs236915	0.0402	6.84e-243	TG	non_coding_transcript_exon_variant	PCSK7	5.8e-09	0.1	Whole Blood	FALSE
rs236915	0.0147	2.434e-11	LDL	non_coding_transcript_exon_variant	PCSK7	1.4e-05	0.21	Pancreas	FALSE
rs236915	0.0147	2.434e-11	LDL	non_coding_transcript_exon_variant	PCSK7	5.8e-09	0.1	Whole Blood	FALSE
rs236919	0.0351	1.18e-216	TG	intron_variant	PCSK7	1.1e-08	0.099	Whole Blood	TRUE
rs236919	0.0351	1.18e-216	TG	intron_variant	PCSK7	3.2e-05	0.2	Pancreas	TRUE
rs236919	0.0223	3.509e-11	HDL	intron_variant	PCSK7	1.1e-08	0.099	Whole Blood	TRUE
rs236919	0.0223	3.509e-11	HDL	intron_variant	PCSK7	3.2e-05	0.2	Pancreas	TRUE
rs236919	0.0137	8.769e-10	LDL	intron_variant	PCSK7	1.1e-08	0.099	Whole Blood	TRUE
rs236919	0.0137	8.769e-10	LDL	intron_variant	PCSK7	3.2e-05	0.2	Pancreas	TRUE
rs2509210	0.0145	1.265e-09	TG	intron_variant	PCSK7	1.3e-05	0.077	Whole Blood	FALSE
rs2929176	0.0154	1.634e-10	TG	intron_variant	PCSK7	2.1e-05	0.075	Whole Blood	FALSE
rs3016582	0.0154	1.677e-10	TG	intron_variant	PCSK7	2.1e-05	0.075	Whole Blood	FALSE
rs35186251	0.0256	5.987e-25	TG	3_prime_UTR_variant	PCSK7	3.3e-06	0.084	Whole Blood	TRUE
rs3736120	-0.0301	3.857e-33	TG	intron_variant	PCSK7	3.4e-06	-0.085	Whole Blood	FALSE
rs4604928	-0.0263	2.837e-22	LDL	intron_variant	PCSK7	9.4e-05	-0.1	Whole Blood	FALSE
rs472785	0.0144	1.551e-09	TG	intron_variant	PCSK7	1.3e-05	0.077	Whole Blood	FALSE
rs488962	0.0267	1.215e-27	TG	5_prime_UTR_variant	PCSK7	1.8e-06	0.087	Whole Blood	FALSE
rs492151	0.0143	1.452e-09	TG	intron_variant	PCSK7	8.9e-06	0.079	Whole Blood	FALSE
rs493139	0.0144	1.336e-09	TG	intron_variant	PCSK7	5.9e-06	0.079	Whole Blood	FALSE
rs4938354	-0.0276	1.559e-29	TG	intron_variant	PCSK7	4.7e-06	-0.084	Whole Blood	FALSE
rs4938355	0.0401	1.971e-33	TG	intron_variant	PCSK7	1.2e-07	0.35	Pancreas	FALSE
rs4938355	0.0206	3.847e-11	LDL	intron_variant	PCSK7	1.2e-07	0.35	Pancreas	FALSE
rs4938357	0.0199	1.213e-10	LDL	non_coding_transcript_exon_variant	PCSK7	4.4e-07	0.34	Pancreas	FALSE
rs4938357	0.0385	1.212e-31	TG	non_coding_transcript_exon_variant	PCSK7	4.4e-07	0.34	Pancreas	FALSE
rs4938361	0.0392	7.495e-34	TG	intron_variant	PCSK7	2.7e-06	0.29	Pancreas	FALSE
rs4938361	0.0198	8.967e-11	LDL	intron_variant	PCSK7	2.7e-06	0.29	Pancreas	FALSE
rs4938362	0.0195	1.623e-10	LDL	intron_variant	PCSK7	2.7e-06	0.29	Pancreas	FALSE
rs4938362	0.0392	7.558e-34	TG	intron_variant	PCSK7	2.7e-06	0.29	Pancreas	FALSE
rs495438	0.0144	1.293e-09	TG	intron_variant	PCSK7	8.9e-06	0.079	Whole Blood	FALSE
rs500389	0.0202	3.951e-09	HDL	5_prime_UTR_variant	PCSK7	4.2e-05	0.19	Pancreas	TRUE
rs500389	0.0202	3.951e-09	HDL	5_prime_UTR_variant	PCSK7	5.2e-09	0.1	Whole Blood	TRUE
rs500389	0.0305	2.89e-212	TG	5_prime_UTR_variant	PCSK7	4.2e-05	0.19	Pancreas	TRUE
rs500389	0.0305	2.89e-212	TG	5_prime_UTR_variant	PCSK7	5.2e-09	0.1	Whole Blood	TRUE
rs505045	0.014	1.8e-09	TG	non_coding_transcript_exon_variant	PCSK7	6.5e-06	0.08	Whole Blood	FALSE
rs507880	0.0145	1.254e-09	TG	intron_variant	PCSK7	1.3e-05	0.077	Whole Blood	FALSE
rs512188	0.0144	1.222e-09	TG	intron_variant	PCSK7	1.3e-05	0.077	Whole Blood	FALSE
rs523366	0.0148	4.367e-10	TG	intron_variant	PCSK7	1.4e-05	0.077	Whole Blood	FALSE
rs529471	0.0154	1.519e-10	TG	intron_variant	PCSK7	1.3e-05	0.077	Whole Blood	FALSE
rs535602	0.0153	3.993e-10	TG	intron_variant	PCSK7	1.2e-05	0.078	Whole Blood	FALSE
rs540922	0.0144	1.582e-09	TG	intron_variant	PCSK7	1.3e-05	0.077	Whole Blood	FALSE
rs552176	0.0144	1.62e-09	TG	intron_variant	PCSK7	1.3e-05	0.077	Whole Blood	FALSE
rs558590	0.0145	1.246e-09	TG	intron_variant	PCSK7	1.3e-05	0.077	Whole Blood	FALSE
rs56061158	0.0386	9.941e-32	TG	non_coding_transcript_exon_variant	PCSK7	1.8e-06	0.33	Pancreas	FALSE
rs56061158	0.02	1.098e-10	LDL	non_coding_transcript_exon_variant	PCSK7	1.8e-06	0.33	Pancreas	FALSE
rs56081989	0.0199	1.428e-10	LDL	non_coding_transcript_exon_variant	PCSK7	4.4e-07	0.34	Pancreas	FALSE
rs56081989	0.0383	2.171e-31	TG	non_coding_transcript_exon_variant	PCSK7	4.4e-07	0.34	Pancreas	FALSE
rs56141924	0.0408	1.667e-36	TG	intron_variant	PCSK7	2.7e-06	0.29	Pancreas	FALSE
rs56141924	0.0207	7.675e-12	LDL	intron_variant	PCSK7	2.7e-06	0.29	Pancreas	FALSE
rs56371319	0.0205	1.848e-11	LDL	intron_variant	PCSK7	1.3e-06	0.31	Pancreas	FALSE
rs56371319	0.0405	1.624e-35	TG	intron_variant	PCSK7	1.3e-06	0.31	Pancreas	FALSE
rs573956	0.0144	1.318e-09	TG	intron_variant	PCSK7	1.3e-05	0.077	Whole Blood	FALSE
rs585849	-0.0263	7.41e-27	TG	upstream_gene_variant	PCSK7	2.1e-06	-0.087	Whole Blood	TRUE
rs588763	0.0146	6.768e-10	TG	intron_variant	PCSK7	1.9e-05	0.076	Whole Blood	FALSE
rs61905477	-0.0686	4.451e-21	TG	intron_variant	PCSK7	5.1e-05	-0.29	Adipose - Subcutaneous	TRUE
rs61905518	-0.0699	1.674e-21	TG	3_prime_UTR_variant	PCSK7	5.5e-05	-0.28	Adipose - Subcutaneous	FALSE
rs61905519	-0.07	1.633e-21	TG	intron_variant	PCSK7	5.5e-05	-0.28	Adipose - Subcutaneous	FALSE
rs61905521	-0.07	1.547e-21	TG	upstream_gene_variant	PCSK7	5.5e-05	-0.28	Adipose - Subcutaneous	FALSE
rs61905522	-0.0701	1.419e-21	TG	upstream_gene_variant	PCSK7	5.5e-05	-0.28	Adipose - Subcutaneous	TRUE
rs61905525	-0.07	1.168e-21	TG	non_coding_transcript_exon_variant	PCSK7	0.00011	-0.27	Adipose - Subcutaneous	FALSE
rs61905526	-0.0702	1.34e-21	TG	3_prime_UTR_variant	PCSK7	5.5e-05	-0.28	Adipose - Subcutaneous	TRUE
rs61905527	-0.0699	1.811e-21	TG	upstream_gene_variant	PCSK7	5.5e-05	-0.28	Adipose - Subcutaneous	FALSE
rs61905530	-0.0691	2.039e-20	TG	intron_variant	PCSK7	5.5e-05	-0.28	Adipose - Subcutaneous	TRUE
rs61905530	0.1579	8.032e-09	HDL2chol	intron_variant	PCSK7	5.5e-05	-0.28	Adipose - Subcutaneous	TRUE
rs61906485	-0.07	3.416e-21	TG	non_coding_transcript_exon_variant	PCSK7	5.5e-05	-0.28	Adipose - Subcutaneous	FALSE
rs61906486	-0.0702	1.389e-21	TG	non_coding_transcript_exon_variant	PCSK7	9.5e-05	-0.28	Adipose - Subcutaneous	FALSE
rs61906487	-0.0702	1.65e-21	TG	non_coding_transcript_exon_variant	PCSK7	5.9e-05	-0.28	Adipose - Subcutaneous	FALSE
rs61906488	-0.0701	1.748e-21	TG	non_coding_transcript_exon_variant	PCSK7	5.9e-05	-0.28	Adipose - Subcutaneous	FALSE
rs61906489	-0.0702	1.729e-21	TG	non_coding_transcript_exon_variant	PCSK7	5.5e-05	-0.28	Adipose - Subcutaneous	FALSE
rs619772	0.0145	1.144e-09	TG	intron_variant	PCSK7	1.4e-05	0.075	Whole Blood	FALSE
rs621858	0.0144	1.607e-09	TG	intron_variant	PCSK7	4.6e-06	0.081	Whole Blood	FALSE
rs634960	0.0266	3.956e-27	TG	5_prime_UTR_variant	PCSK7	2,00E-06	0.087	Whole Blood	FALSE
rs644215	0.0144	1.226e-09	TG	intron_variant	PCSK7	6.6e-06	0.079	Whole Blood	FALSE
rs652455	-0.0512	1.16e-250	TG	intron_variant	PCSK7	4.2e-06	-0.096	Whole Blood	FALSE
rs655023	0.0144	1.557e-09	TG	intron_variant	PCSK7	5.6e-06	0.08	Whole Blood	FALSE
rs6589602	-0.0162	1.163e-13	LDL	intron_variant	PCSK7	1.6e-05	-0.2	Pancreas	TRUE
rs6589602	-0.0162	1.163e-13	LDL	intron_variant	PCSK7	1.8e-08	-0.097	Whole Blood	TRUE
rs6589602	-0.0448	4.62e-256	TG	intron_variant	PCSK7	1.6e-05	-0.2	Pancreas	TRUE
rs6589602	-0.0448	4.62e-256	TG	intron_variant	PCSK7	1.8e-08	-0.097	Whole Blood	TRUE
rs6589603	-0.094	1.45000000002174e-314	TG	intron_variant	PCSK7	2.1e-05	-0.11	Whole Blood	FALSE
rs6589603	-0.0281	4.973e-22	LDL	intron_variant	PCSK7	2.1e-05	-0.11	Whole Blood	FALSE
rs659955	0.0276	2.593e-23	LDL	non_coding_transcript_exon_variant	PCSK7	3.3e-05	0.11	Whole Blood	FALSE
rs664011	0.0151	2.772e-10	TG	intron_variant	PCSK7	8.3e-06	0.079	Whole Blood	FALSE
rs664082	-0.0257	4.796e-26	TG	intron_variant	PCSK7	2.7e-06	-0.086	Whole Blood	FALSE
rs664922	-0.0266	1.382e-27	TG	intron_variant	PCSK7	2.7e-06	-0.086	Whole Blood	FALSE
rs664971	-0.0268	1.309e-27	TG	intron_variant	PCSK7	2.1e-06	-0.087	Whole Blood	FALSE

rs67201490	0.02	9.414e-11	LDL	non_coding_transcript_exon_variant	PCSK7	4.4e-07	0.34	Pancreas	TRUE
rs67201490	0.0386	7.37e-32	TG	non_coding_transcript_exon_variant	PCSK7	4.4e-07	0.34	Pancreas	TRUE
rs672177	0.0146	8.213e-10	TG	intron_variant	PCSK7	1.3e-05	0.077	Whole Blood	FALSE
rs673738	0.0145	1.118e-09	TG	intron_variant	PCSK7	1.3e-05	0.077	Whole Blood	FALSE
rs678837	0.0147	4.632e-10	TG	intron_variant	PCSK7	2.1e-05	0.075	Whole Blood	FALSE
rs68137714	0.0207	1.986e-11	LDL	intron_variant	PCSK7	1.2e-07	0.35	Pancreas	FALSE
rs68137714	0.0401	8.325e-34	TG	intron_variant	PCSK7	1.2e-07	0.35	Pancreas	FALSE
rs7107152	-0.0272	5.15e-29	TG	intron_variant	PCSK7	2.8e-06	-0.086	Whole Blood	FALSE
rs7120565	-0.0441	4.64e-256	TG	intron_variant	PCSK7	1.8e-08	-0.097	Whole Blood	FALSE
rs7120565	-0.0441	4.64e-256	TG	intron_variant	PCSK7	1.6e-05	-0.2	Pancreas	FALSE
rs7120565	-0.0159	3.147e-13	LDL	intron_variant	PCSK7	1.8e-08	-0.097	Whole Blood	FALSE
rs7120565	-0.0159	3.147e-13	LDL	intron_variant	PCSK7	1.6e-05	-0.2	Pancreas	FALSE
rs7122944	-0.0945	5.873e-39	TG	non_coding_transcript_exon_variant	PCSK7	9.4e-05	-0.1	Whole Blood	FALSE
rs7122944	-0.0316	1.75e-10	TG	non_coding_transcript_exon_variant	PCSK7	9.4e-05	-0.1	Whole Blood	FALSE
rs7122944	-0.0262	3.139e-21	LDL	non_coding_transcript_exon_variant	PCSK7	9.4e-05	-0.1	Whole Blood	FALSE
rs73008568	0.0407	1.164e-34	TG	intron_variant	PCSK7	7,00E-07	0.35	Pancreas	TRUE
rs73008568	0.0209	1.563e-11	LDL	intron_variant	PCSK7	7,00E-07	0.35	Pancreas	TRUE
rs74830	0.0134	2.254e-09	LDL	non_coding_transcript_exon_variant	PCSK7	9.5e-06	0.21	Pancreas	TRUE
rs74830	0.0134	2.254e-09	LDL	non_coding_transcript_exon_variant	PCSK7	4.4e-09	0.1	Whole Blood	TRUE
rs74830	0.0343	1.7e-214	TG	non_coding_transcript_exon_variant	PCSK7	9.5e-06	0.21	Pancreas	TRUE
rs74830	0.0343	1.7e-214	TG	non_coding_transcript_exon_variant	PCSK7	4.4e-09	0.1	Whole Blood	TRUE
rs74830	0.0236	3.635e-11	HDL	non_coding_transcript_exon_variant	PCSK7	9.5e-06	0.21	Pancreas	TRUE
rs74830	0.0236	3.635e-11	HDL	non_coding_transcript_exon_variant	PCSK7	4.4e-09	0.1	Whole Blood	TRUE
rs76830818	-0.07	1.792e-21	TG	non_coding_transcript_exon_variant	PCSK7	5.9e-05	-0.28	Adipose - Subcutaneous	FALSE
rs77086432	-0.07	1.538e-21	TG	3_prime_UTR_variant	PCSK7	5.5e-05	-0.28	Adipose - Subcutaneous	FALSE
rs7925044	0.0143	1.531e-09	TG	intron_variant	PCSK7	4.4e-06	0.081	Whole Blood	FALSE
rs7941150	0.0199	1.281e-10	LDL	non_coding_transcript_exon_variant	PCSK7	3.3e-08	0.36	Pancreas	TRUE
rs7941150	0.0381	3.913e-31	TG	non_coding_transcript_exon_variant	PCSK7	3.3e-08	0.36	Pancreas	TRUE
rs7946257	-0.0259	1.181e-27	TG	non_coding_transcript_exon_variant	PCSK7	3.5e-06	-0.085	Whole Blood	FALSE
rs8521	-0.0262	3.113e-27	TG	3_prime_UTR_variant	PCSK7	4.1e-06	-0.084	Whole Blood	FALSE
NA	NA	NA	NA	NA	NA	NA	NA	NA	FALSE
rs10888896	0.0384	2.95e-263	LDL	intron_variant	PCSK9	2.7e-05	-0.28	Adipose - Visceral (Omentum)	FALSE
rs111835689	0.0467	5.22e-284	LDL	intergenic_variant	PCSK9	6,00E-13	0.54	Adipose - Visceral (Omentum)	FALSE
rs11206506	0.0417	2.245e-40	LDL	intergenic_variant	PCSK9	1.1e-14	0.57	Adipose - Visceral (Omentum)	FALSE
rs11206507	0.0417	2.226e-40	LDL	intergenic_variant	PCSK9	2.3e-14	0.55	Adipose - Visceral (Omentum)	FALSE
rs11206508	0.0406	8.692e-37	LDL	intergenic_variant	PCSK9	5.3e-15	0.57	Adipose - Visceral (Omentum)	FALSE
rs11206509	0.0425	4.645e-42	LDL	regulatory_region_variant	PCSK9	1.7e-15	0.59	Adipose - Visceral (Omentum)	FALSE
rs11206510	-0.0598	1.72e-257	LDL	intergenic_variant	PCSK9	1.5e-06	-0.15	Whole Blood	FALSE
rs11206510	-0.0487	7.448e-17	CAD	intergenic_variant	PCSK9	1.5e-06	-0.15	Whole Blood	FALSE
rs11206510	-0.0482	2.629e-17	CHOL	intergenic_variant	PCSK9	1.5e-06	-0.15	Whole Blood	FALSE
rs112636762	-0.0218	2.441e-18	LDL	intergenic_variant	PCSK9	1.8e-09	-0.34	Adipose - Visceral (Omentum)	FALSE
rs11488228	0.0487	1,00E-300	LDL	intergenic_variant	PCSK9	5.4e-05	0.14	Whole Blood	FALSE
rs11488228	0.0487	1,00E-300	LDL	intergenic_variant	PCSK9	3,00E-13	0.53	Adipose - Visceral (Omentum)	FALSE
rs11583723	-0.0229	7.994e-16	LDL	5_prime_UTR_variant	PCSK9	7.5e-08	-0.21	Whole Blood	TRUE
rs11583723	-0.0229	7.994e-16	LDL	5_prime_UTR_variant	PCSK9	1.1e-05	-0.42	Pancreas	TRUE
rs11583723	-0.0363	2.287e-14	CHOL	5_prime_UTR_variant	PCSK9	7.5e-08	-0.21	Whole Blood	TRUE
rs11583723	-0.0363	2.287e-14	CHOL	5_prime_UTR_variant	PCSK9	1.1e-05	-0.42	Pancreas	TRUE
rs11583974	0.0413	9.827e-14	LDL	splice_region_variant	PCSK9	3.5e-05	0.26	Whole Blood	FALSE
rs11588151	-0.0576	2.19e-282	LDL	regulatory_region_variant	PCSK9	1.4e-06	-0.15	Whole Blood	FALSE
rs11588151	-0.0576	2.19e-282	LDL	regulatory_region_variant	PCSK9	2.5e-06	-0.34	Adipose - Visceral (Omentum)	FALSE
rs11588151	-0.0491	2.336e-15	CHOL	regulatory_region_variant	PCSK9	1.4e-06	-0.15	Whole Blood	FALSE
rs11588151	-0.0491	2.336e-15	CHOL	regulatory_region_variant	PCSK9	2.5e-06	-0.34	Adipose - Visceral (Omentum)	FALSE
rs11588151	-0.0534	2.422e-16	CAD	regulatory_region_variant	PCSK9	1.4e-06	-0.15	Whole Blood	FALSE
rs11588151	-0.0534	2.422e-16	CAD	regulatory_region_variant	PCSK9	2.5e-06	-0.34	Adipose - Visceral (Omentum)	FALSE
rs1165285	0.0272	1.273e-36	LDL	intron_variant	PCSK9	3.3e-09	0.15	Whole Blood	FALSE
rs1165285	0.0234	4.714e-11	CHOL	intron_variant	PCSK9	3.3e-09	0.15	Whole Blood	FALSE
rs1165287	0.0481	1.35e-293	LDL	intron_variant	PCSK9	1.1e-06	0.35	Liver	FALSE
rs1165287	0.0481	1.35e-293	LDL	intron_variant	PCSK9	1.2e-11	0.17	Whole Blood	FALSE
rs1165287	0.0337	1.91e-20	CHOL	intron_variant	PCSK9	1.1e-06	0.35	Liver	FALSE
rs1165287	0.0337	1.91e-20	CHOL	intron_variant	PCSK9	1.2e-11	0.17	Whole Blood	FALSE
rs12046679	0.0346	1.531e-52	LDL	intergenic_variant	PCSK9	3.3e-23	0.56	Adipose - Visceral (Omentum)	FALSE
rs12046679	0.0346	1.531e-52	LDL	intergenic_variant	PCSK9	6.9e-05	0.1	Whole Blood	FALSE
rs12046679	0.0257	1.185e-09	CHOL	intergenic_variant	PCSK9	3.3e-23	0.56	Adipose - Visceral (Omentum)	FALSE
rs12046679	0.0257	1.185e-09	CHOL	intergenic_variant	PCSK9	6.9e-05	0.1	Whole Blood	FALSE
rs12117661	-0.0422	1.067e-17	CHOL	regulatory_region_variant	PCSK9	2.4e-05	-0.21	Artery - Tibial	TRUE
rs12117661	-0.0422	1.067e-17	CHOL	regulatory_region_variant	PCSK9	1.2e-12	-0.46	Adipose - Visceral (Omentum)	TRUE
rs12117661	-0.0422	1.067e-17	CHOL	regulatory_region_variant	PCSK9	8.3e-11	-0.19	Whole Blood	TRUE
rs12117661	-0.0422	1.067e-17	CHOL	regulatory_region_variant	PCSK9	7.9e-05	-0.21	Adipose - Subcutaneous	TRUE
rs12117661	-0.0471	2.321e-15	CAD	regulatory_region_variant	PCSK9	2.4e-05	-0.21	Artery - Tibial	TRUE
rs12117661	-0.0471	2.321e-15	CAD	regulatory_region_variant	PCSK9	1.2e-12	-0.46	Adipose - Visceral (Omentum)	TRUE
rs12117661	-0.0471	2.321e-15	CAD	regulatory_region_variant	PCSK9	8.3e-11	-0.19	Whole Blood	TRUE
rs12117661	-0.0471	2.321e-15	CAD	regulatory_region_variant	PCSK9	7.9e-05	-0.21	Adipose - Subcutaneous	TRUE
rs12117661	-0.0622	5.24e-289	LDL	regulatory_region_variant	PCSK9	2.4e-05	-0.21	Artery - Tibial	TRUE
rs12117661	-0.0622	5.24e-289	LDL	regulatory_region_variant	PCSK9	1.2e-12	-0.46	Adipose - Visceral (Omentum)	TRUE
rs12117661	-0.0622	5.24e-289	LDL	regulatory_region_variant	PCSK9	8.3e-11	-0.19	Whole Blood	TRUE
rs12117661	-0.0622	5.24e-289	LDL	regulatory_region_variant	PCSK9	7.9e-05	-0.21	Adipose - Subcutaneous	TRUE
rs12739394	-0.0338	1.168e-50	LDL	intergenic_variant	PCSK9	9,00E-24	-0.57	Adipose - Visceral (Omentum)	FALSE
rs12739394	-0.0338	1.168e-50	LDL	intergenic_variant	PCSK9	2.5e-05	-0.11	Whole Blood	FALSE
rs12739394	-0.024	2.417e-11	CHOL	intergenic_variant	PCSK9	9,00E-24	-0.57	Adipose - Visceral (Omentum)	FALSE
rs12739394	-0.024	2.417e-11	CHOL	intergenic_variant	PCSK9	2.5e-05	-0.11	Whole Blood	FALSE
rs12739506	-0.0347	1.702e-52	LDL	intergenic_variant	PCSK9	3.4e-05	-0.11	Whole Blood	FALSE
rs12739506	-0.0347	1.702e-52	LDL	intergenic_variant	PCSK9	6.6e-24	-0.57	Adipose - Visceral (Omentum)	FALSE
rs12739506	-0.0254	1.924e-09	CHOL	intergenic_variant	PCSK9	3.4e-05	-0.11	Whole Blood	FALSE
rs12739506	-0.0254	1.924e-09	CHOL	intergenic_variant	PCSK9	6.6e-24	-0.57	Adipose - Visceral (Omentum)	FALSE
rs12748266	-0.042	1,00E-12	CHOL	intergenic_variant	PCSK9	9.5e-08	-0.37	Adipose - Visceral (Omentum)	FALSE
rs12748266	-0.042	1,00E-12	CHOL	intergenic_variant	PCSK9	1.3e-07	-0.16	Whole Blood	FALSE
rs12748266	-0.0573	5.34e-279	LDL	intergenic_variant	PCSK9	9.5e-08	-0.37	Adipose - Visceral (Omentum)	FALSE
rs12748266	-0.0573	5.34e-279	LDL	intergenic_variant	PCSK9	1.3e-07	-0.16	Whole Blood	FALSE
rs12748266	-0.0518	1.18e-13	CAD	intergenic_variant	PCSK9	9.5e-08	-0.37	Adipose - Visceral (Omentum)	FALSE
rs12748266	-0.0518	1.18e-13	CAD	intergenic_variant	PCSK9	1.3e-07	-0.16	Whole Blood	FALSE
rs138483078	0.0446	5.71e-282	LDL	intergenic_variant	PCSK9	1.7e-15	0.59	Adipose - Visceral (Omentum)	FALSE
rs17111483	-0.0298	1.884e-14	LDL	intergenic_variant	PCSK9	1.3e-07	-0.46	Adipose - Visceral (Omentum)	FALSE
rs17192725	0.0419	2.172e-25	LDL	intergenic_variant	PCSK9	1.9e-11	0.57	Adipose - Visceral (Omentum)	FALSE
rs1887552	-0.0232	6.206e-11	CHOL	regulatory_region_variant	PCSK9	2.7e-05	-0.11	Whole Blood	FALSE
rs1887552	-0.0232	6.206e-11	CHOL	regulatory_region_variant	PCSK9	4.1e-24	-0.58	Adipose - Visceral (Omentum)	FALSE
rs1887552	-0.033	7.365e-48	LDL	regulatory_region_variant	PCSK9	2.7e-05	-0.11	Whole Blood	FALSE
rs1887552	-0.033	7.365e-48	LDL	regulatory_region_variant	PCSK9	4.1e-24	-0.58	Adipose - Visceral (Omentum)	FALSE
rs2479391	-0.0303	3.399e-12	CHOL	intergenic_variant	PCSK9	4.7e-09	-0.53	Adipose - Visceral (Omentum)	FALSE
rs2479391	-0.0253	1.348e-14	LDL	intergenic_variant	PCSK9	4.7e-09	-0.53	Adipose - Visceral (Omentum)	FALSE
rs2479392	-0.0277	6.67e-10	CHOL	intergenic_variant	PCSK9	5.9e-06	-0.36	Adipose - Visceral (Omentum)	FALSE
rs2479393	-0.0276	7.605e-14	CHOL	TF_binding_site_variant	PCSK9	1,00E-06	-0.13	Whole Blood	TRUE
rs2479393	-0.0276	7.605e-14	CHOL	TF_binding_site_variant	PCSK9	1.6e-25	-0.59	Adipose - Visceral (Omentum)	TRUE
rs2479393	-0.038	1.02e-282	LDL	TF_binding_site_variant	PCSK9	1,00E-06	-0.13	Whole Blood	TRUE
rs2479393	-0.038	1.02e-282	LDL	TF_binding_site_variant	PCSK9	1.6e-25	-0.59	Adipose - Visceral (Omentum)	TRUE
rs2479394	-0.0233	4.536e-11	CHOL	intergenic_variant	PCSK9	2.3e-05	-0.11	Whole Blood	FALSE
rs2479394	-0.0233	4.536e-11	CHOL	intergenic_variant	PCSK9	8.9e-24	-0.57	Adipose - Visceral (Omentum)	FALSE
rs2479394	-0.0316	2.474e-44	LDL	intergenic_variant	PCSK9	2.3e-05	-0.11	Whole Blood	FALSE
rs2479394	-0.0316	2.474e-44	LDL	intergenic_variant	PCSK9	8.9e-24	-0.57	Adipose - Visceral (Omentum)	FALSE
rs2479408	-0.0397	1.796e-42	LDL	upstream_gene_variant	PCSK9	3.1e-05	0.29	Adipose - Visceral (Omentum)	TRUE
rs2479409	-0.0348	1.94e-206	LDL	upstream_gene_variant	PCSK9	4.2e-05	-0.23	Spleen	TRUE
rs2479413	-0.0485	5.84e-289	LDL	intron_variant	PCSK9	2.9e-05	-0.1	Whole Blood	FALSE
rs2479413	-0.0348	1.651e-20	CHOL	intron_variant	PCSK9	2.9e-05	-0.1	Whole Blood	FALSE
rs2479415	-0.033	2.12e-259	LDL	regulatory_region_variant	PCSK9	8.1e-06	0.29	Pancreas	TRUE
rs2479417	-0.0194	9.468e-20	LDL	intergenic_variant	PCSK9	1.3e-10	-0.36	Adipose - Visceral (Omentum)	FALSE
rs2479420	-0.0387	1,00E-300	LDL	intergenic_variant	PCSK9	4.6e-06	-0.12	Whole Blood	TRUE
rs2479420	-0.0387	1,00E-300	LDL	intergenic_variant	PCSK9	2.3e-28	-0.62	Adipose - Visceral (Omentum)	TRUE
rs2495477	-0.0382	6.891e-30	CHOL	splice_region_variant	PCSK9	6.3e-12	-0.17	Whole Blood	TRUE
rs2495477	-0.0487	3.73e-281	LDL	splice_region_variant	PCSK9	6.3e-12	-0.17	Whole Blood	TRUE
rs2495477	-0.1063	2.336e-14	IDLchol	splice_region_variant	PCSK9	6.3e-12	-0.17	Whole Blood	TRUE
rs2495477	-0.1004	5.683e-13	IDLpart	splice_region_variant	PCSK9	6.3e-12	-0.17	Whole Blood	TRUE
rs2495477	-0.082	1.989e-11	ApoB	splice_region_variant	PCSK9	6.3e-12	-0.17	Whole Blood	TRUE
rs2495495	-0.0232	2.509e-13	LDL	intergenic_variant	PCSK9	8.6e-08	-0.46	Adipose - Visceral (Omentum)	FALSE
rs2495495	-0.0215	9.834e-09	CHOL	intergenic_variant	PCSK9	8.6e-08	-0.46	Adipose - Visceral (Omentum)	FALSE
rs2495497	-0.0247	6.493e-15	LDL	intergenic_variant	PCSK9	2.4e-10	-0.54	Adipose - Visceral (Omentum)	FALSE
rs2495497	-0.0263	4.534e-11	CHOL	intergenic_variant	PCSK9	2.4e-10	-0.54	Adipose - Visceral (Omentum)	FALSE
rs2495498	-0.0276	5.958e-12	CHOL	intergenic_variant	PCSK9	7.9e-09	-0.52	Adipose - Visceral (Omentum)	FALSE
rs2495498	-0.0253	2.273e-14	LDL	intergenic_variant	PCSK9	7.9e-09	-0.52	Adipose - Visceral (Omentum)	FALSE
rs2495500	-0.0331	3.734e-48	LDL	regulatory_region_variant	PCSK9	2.7e-05	-0.11	Whole Blood	TRUE
rs2495500	-0.0331	3.734e-48	LDL	regulatory_region_variant	PCSK9	4.1e-24	-0.58	Adipose - Visceral (Omentum)	TRUE
rs2495500	-0.0233	5.344e-11	CHOL	regulatory_region_variant	PCSK9	2.7e-05	-0.11	Whole Blood	TRUE
rs2495500	-0.0233	5.344e-11	CHOL	regulatory_region_variant	PCSK9	4.1e-24	-0.58	Adipose - Visceral (Omentum)	TRUE
rs2495501	-0.0282	3.746e-12	CHOL	intergenic_variant	PCSK9	4.7e-09	-0.53	Adipose - Visceral (Omentum)	FALSE
rs2495501	-0.0238	1.996e-13	LDL	intergenic_variant	PCSK9	4.7e-09	-0.53	Adipose - Visceral (Omentum)	FALSE
rs2495503	-0.0371	3.67e-283	LDL	intergenic_variant	PCSK9	1.1e-06	-0.13	Whole Blood	TRUE
rs2495503	-0.0371	3.67e-283	LDL	intergenic_variant	PCSK9	1.3e-26	-0.61	Adipose - Visceral (Omentum)	TRUE
rs2495503	-0.0282	3.392e-14	CHOL	intergenic_variant	PCSK9	1.1e-06	-0.13	Whole Blood	TRUE
rs2495503	-0.0282	3.392e-14	CHOL	intergenic_variant	PCSK9	1.3e-26	-0.61	Adipose - Visceral (Omentum)	TRUE
rs2495504	-0.032	4.507e-46	LDL	intergenic_variant	PCSK9	5.4e-05	-0.1	Whole Blood	FALSE
rs2495504	-0.032	4.507e-46	LDL	intergenic_variant	PCSK9	2.6e-23	-0.56	Adipose - Visceral (Omentum)	FALSE
rs2495504	-0.024	1.753e-11	CHOL	intergenic_variant	PCSK9	5.4e-05	-0.1	Whole Blood	FALSE
rs2495504	-0.024	1.753e-11	CHOL	intergenic_variant	PCSK9	2.6e-23	-0.56	Adipose - Visceral (Omentum)	FALSE
rs28385704	-0.0357	3.794e-14	CHOL	intron_variant	PCSK9	7.5e-08	-0.21	Whole Blood	FALSE
rs28385704	-0.0357	3.794e-14	CHOL	intron_variant	PCSK9	1.1e-05	-0.42	Pancreas	FALSE
rs28385704	-0.0229	8.437e-16	LDL	intron_variant	PCSK9	7.5e-08	-0.21	Whole Blood	FALSE
rs28385704	-0.0229	8.437e-16	LDL	intron_variant	PCSK9	1.1e-05	-0.42	Pancreas	FALSE
rs28862456	0.049	1,00E-300	LDL	intergenic_variant	PCSK9	1.8e-14	0.58	Adipose - Visceral (Omentum)	FALSE
rs28882490	0.049	1,00E-300	LDL	intergenic_variant	PCSK9	4.5e-14	0.57	Adipose - Visceral (Omentum)	FALSE
rs2986945	-0.0357	3.507e-14	CHOL	intergenic_variant	PCSK9	5.2e-09	-0.51	Adipose - Visceral (Omentum)	FALSE
rs2986945	-0.0278	3.625e-19	LDL	intergenic_variant	PCSK9	5.2e-09	-0.51	Adipose - Visceral (Omentum)	FALSE
rs3118721	-0.0386	1,00E-300	LDL	intergenic_variant	PCSK9	2.5e-27	-0.61	Adipose - Visceral (Omentum)	FALSE

rs3118721	-0.0386	1,00E-300	LDL	intergenic_variant	PCSK9	9.4e-06	-0.12	Whole Blood	FALSE
rs3118722	-0.0386	1,00E-300	LDL	intergenic_variant	PCSK9	9.4e-06	-0.12	Whole Blood	FALSE
rs3118722	-0.0386	1,00E-300	LDL	intergenic_variant	PCSK9	2.5e-27	-0.61	Adipose - Visceral (Omentum)	FALSE
rs34232196	-0.0624	1.19e-290	LDL	intergenic_variant	PCSK9	1.4e-12	-0.46	Adipose - Visceral (Omentum)	TRUE
rs34232196	-0.0624	1.19e-290	LDL	intergenic_variant	PCSK9	9.5e-05	-0.2	Adipose - Subcutaneous	TRUE
rs34232196	-0.0624	1.19e-290	LDL	intergenic_variant	PCSK9	7.3e-11	-0.19	Whole Blood	TRUE
rs34232196	-0.0624	1.19e-290	LDL	intergenic_variant	PCSK9	1.8e-05	-0.22	Artery - Tibial	TRUE
rs34232196	-0.0483	8.817e-14	CAD	intergenic_variant	PCSK9	1.4e-12	-0.46	Adipose - Visceral (Omentum)	TRUE
rs34232196	-0.0483	8.817e-14	CAD	intergenic_variant	PCSK9	9.5e-05	-0.2	Adipose - Subcutaneous	TRUE
rs34232196	-0.0483	8.817e-14	CAD	intergenic_variant	PCSK9	7.3e-11	-0.19	Whole Blood	TRUE
rs34232196	-0.0483	8.817e-14	CAD	intergenic_variant	PCSK9	1.8e-05	-0.22	Artery - Tibial	TRUE
rs34232196	-0.0438	2.242e-18	CHOL	intergenic_variant	PCSK9	1.4e-12	-0.46	Adipose - Visceral (Omentum)	TRUE
rs34232196	-0.0438	2.242e-18	CHOL	intergenic_variant	PCSK9	9.5e-05	-0.2	Adipose - Subcutaneous	TRUE
rs34232196	-0.0438	2.242e-18	CHOL	intergenic_variant	PCSK9	7.3e-11	-0.19	Whole Blood	TRUE
rs34232196	-0.0438	2.242e-18	CHOL	intergenic_variant	PCSK9	1.8e-05	-0.22	Artery - Tibial	TRUE
rs41294819	-0.0353	7.936e-14	CHOL	upstream_gene_variant	PCSK9	1.4e-05	-0.41	Pancreas	TRUE
rs41294819	-0.0353	7.936e-14	CHOL	upstream_gene_variant	PCSK9	5.3e-07	-0.19	Whole Blood	TRUE
rs41294819	-0.0226	1.652e-15	LDL	upstream_gene_variant	PCSK9	1.4e-05	-0.41	Pancreas	TRUE
rs41294819	-0.0226	1.652e-15	LDL	upstream_gene_variant	PCSK9	5.3e-07	-0.19	Whole Blood	TRUE
rs41294823	-0.0836	5.825e-18	CHOL	intron_variant	PCSK9	9,00E-05	-0.16	Whole Blood	FALSE
rs41294823	-0.0845	5.723e-14	LDL	intron_variant	PCSK9	9,00E-05	-0.16	Whole Blood	FALSE
rs41294825	-0.0415	1.672e-16	LDL	intron_variant	PCSK9	8.6e-05	-0.19	Whole Blood	FALSE
rs4500361	-0.0608	1,00E-300	LDL	intergenic_variant	PCSK9	2.7e-05	-0.22	Adipose - Subcutaneous	TRUE
rs4500361	-0.0608	1,00E-300	LDL	intergenic_variant	PCSK9	4,00E-10	-0.18	Whole Blood	TRUE
rs4500361	-0.0608	1,00E-300	LDL	intergenic_variant	PCSK9	1.6e-11	-0.44	Adipose - Visceral (Omentum)	TRUE
rs45448095	-0.0221	1.647e-14	LDL	5_prime_UTR_variant	PCSK9	7.5e-08	-0.21	Whole Blood	FALSE
rs45448095	-0.0221	1.647e-14	LDL	5_prime_UTR_variant	PCSK9	1.1e-05	-0.42	Pancreas	FALSE
rs45448095	-0.0338	1.027e-12	CHOL	5_prime_UTR_variant	PCSK9	7.5e-08	-0.21	Whole Blood	FALSE
rs45448095	-0.0338	1.027e-12	CHOL	5_prime_UTR_variant	PCSK9	1.1e-05	-0.42	Pancreas	FALSE
rs45530931	-0.0355	1.164e-13	CHOL	intron_variant	PCSK9	3.7e-06	-0.17	Whole Blood	FALSE
rs45530931	-0.0212	1.783e-13	LDL	intron_variant	PCSK9	3.7e-06	-0.17	Whole Blood	FALSE
rs45613943	-0.0405	4.718e-16	LDL	intron_variant	PCSK9	2,00E-05	-0.2	Whole Blood	FALSE
rs4609471	-0.0637	1,00E-300	LDL	intergenic_variant	PCSK9	6.6e-11	-0.19	Whole Blood	TRUE
rs4609471	-0.0637	1,00E-300	LDL	intergenic_variant	PCSK9	8.8e-12	-0.45	Adipose - Visceral (Omentum)	TRUE
rs471705	0.0332	2.088e-20	CHOL	intron_variant	PCSK9	9.4e-12	0.17	Whole Blood	FALSE
rs471705	0.0332	2.088e-20	CHOL	intron_variant	PCSK9	1.1e-06	0.35	Liver	FALSE
rs471705	0.0332	2.088e-20	CHOL	intron_variant	PCSK9	3.7e-05	0.23	Spleen	FALSE
rs471705	0.0479	1.88e-293	LDL	intron_variant	PCSK9	9.4e-12	0.17	Whole Blood	FALSE
rs471705	0.0479	1.88e-293	LDL	intron_variant	PCSK9	1.1e-06	0.35	Liver	FALSE
rs471705	0.0479	1.88e-293	LDL	intron_variant	PCSK9	3.7e-05	0.23	Spleen	FALSE
rs472495	0.05	1.94e-294	LDL	intron_variant	PCSK9	8.8e-14	0.19	Whole Blood	TRUE
rs472495	0.05	1.94e-294	LDL	intron_variant	PCSK9	1.3e-05	0.23	Spleen	TRUE
rs472495	0.05	1.94e-294	LDL	intron_variant	PCSK9	7.7e-06	0.33	Liver	TRUE
rs472495	0.0336	1.484e-20	CHOL	intron_variant	PCSK9	8.8e-14	0.19	Whole Blood	TRUE
rs472495	0.0336	1.484e-20	CHOL	intron_variant	PCSK9	1.3e-05	0.23	Spleen	TRUE
rs472495	0.0336	1.484e-20	CHOL	intron_variant	PCSK9	7.7e-06	0.33	Liver	TRUE
rs479832	0.023	1.759e-18	LDL	intron_variant	PCSK9	2.7e-06	0.36	Pancreas	FALSE
rs479832	0.0326	3.03e-14	CHOL	intron_variant	PCSK9	2.7e-06	0.36	Pancreas	FALSE
rs479910	0.0468	1.8e-305	LDL	intron_variant	PCSK9	2.6e-05	0.25	Adipose - Visceral (Omentum)	TRUE
rs479910	0.0468	1.8e-305	LDL	intron_variant	PCSK9	1.1e-09	0.15	Whole Blood	TRUE
rs479910	0.0468	1.8e-305	LDL	intron_variant	PCSK9	1.3e-05	0.32	Liver	TRUE
rs479910	0.044	3.585e-21	CHOL	intron_variant	PCSK9	2.6e-05	0.25	Adipose - Visceral (Omentum)	TRUE
rs479910	0.044	3.585e-21	CHOL	intron_variant	PCSK9	1.1e-09	0.15	Whole Blood	TRUE
rs479910	0.044	3.585e-21	CHOL	intron_variant	PCSK9	1.3e-05	0.32	Liver	TRUE
rs483462	0.0188	2.508e-14	LDL	intron_variant	PCSK9	1.3e-05	0.12	Whole Blood	FALSE
rs4927191	-0.0583	1,00E-300	LDL	intergenic_variant	PCSK9	2.3e-14	-0.47	Adipose - Visceral (Omentum)	TRUE
rs4927191	-0.0583	1,00E-300	LDL	intergenic_variant	PCSK9	2.9e-11	-0.18	Whole Blood	TRUE
rs4927191	-0.0583	1,00E-300	LDL	intergenic_variant	PCSK9	0.00011	-0.19	Adipose - Subcutaneous	TRUE
rs4927191	-0.0583	1,00E-300	LDL	intergenic_variant	PCSK9	4.4e-06	-0.22	Artery - Tibial	TRUE
rs4927193	-0.0358	3.321e-14	CHOL	intron_variant	PCSK9	3.7e-05	-0.15	Whole Blood	FALSE
rs4927193	-0.0208	6.67e-13	LDL	intron_variant	PCSK9	3.7e-05	-0.15	Whole Blood	FALSE
rs494198	0.0932	3.414e-11	IDLpart	intron_variant	PCSK9	2.1e-08	0.14	Whole Blood	TRUE
rs494198	0.0483	8.09e-290	LDL	intron_variant	PCSK9	2.1e-08	0.14	Whole Blood	TRUE
rs494198	0.1017	4.943e-13	IDLchol	intron_variant	PCSK9	2.1e-08	0.14	Whole Blood	TRUE
rs494198	0.038	4.673e-27	CHOL	intron_variant	PCSK9	2.1e-08	0.14	Whole Blood	TRUE
rs494198	0.0789	1.71e-10	ApoB	intron_variant	PCSK9	2.1e-08	0.14	Whole Blood	TRUE
rs499718	0.0344	5.265e-16	CHOL	intron_variant	PCSK9	2.7e-06	0.36	Pancreas	FALSE
rs499718	0.0205	1.56e-15	LDL	intron_variant	PCSK9	2.7e-06	0.36	Pancreas	FALSE
rs499883	0.0329	3.856e-19	CHOL	intron_variant	PCSK9	6.3e-12	0.17	Whole Blood	FALSE
rs499883	0.0471	5.86e-287	LDL	intron_variant	PCSK9	6.3e-12	0.17	Whole Blood	FALSE
rs502576	-0.0379	5.188e-41	LDL	intron_variant	PCSK9	5,00E-07	0.37	Adipose - Visceral (Omentum)	TRUE
rs521662	0.033	1.13e-18	CHOL	intron_variant	PCSK9	1.1e-08	0.15	Whole Blood	FALSE
rs521662	0.0475	2.84e-287	LDL	intron_variant	PCSK9	1.1e-08	0.15	Whole Blood	FALSE
rs529787	-0.0386	7.824e-45	LDL	intron_variant	PCSK9	7.3e-07	0.37	Adipose - Visceral (Omentum)	FALSE
rs531701	0.0342	1.486e-15	CHOL	intron_variant	PCSK9	1,00E-06	0.38	Pancreas	FALSE
rs531701	0.0222	1.728e-17	LDL	intron_variant	PCSK9	1,00E-06	0.38	Pancreas	FALSE
rs534473	0.0336	4.579e-15	CHOL	intron_variant	PCSK9	1,00E-06	0.38	Pancreas	FALSE
rs534473	0.0219	3.603e-17	LDL	intron_variant	PCSK9	1,00E-06	0.38	Pancreas	FALSE
rs535471	0.0331	1.097e-14	CHOL	intron_variant	PCSK9	4,00E-07	0.4	Pancreas	TRUE
rs535471	0.022	2.619e-17	LDL	intron_variant	PCSK9	4,00E-07	0.4	Pancreas	TRUE
rs536607133	0.0486	1,00E-300	LDL	intergenic_variant	PCSK9	5.7e-14	0.56	Adipose - Visceral (Omentum)	FALSE
rs553741	0.0476	7.87e-294	LDL	intron_variant	PCSK9	6,00E-07	0.36	Liver	TRUE
rs553741	0.0476	7.87e-294	LDL	intron_variant	PCSK9	2.9e-11	0.17	Whole Blood	TRUE
rs553741	0.0338	1.4e-20	CHOL	intron_variant	PCSK9	6,00E-07	0.36	Liver	TRUE
rs553741	0.0338	1.4e-20	CHOL	intron_variant	PCSK9	2.9e-11	0.17	Whole Blood	TRUE
rs557211	0.0218	6.639e-17	LDL	intron_variant	PCSK9	2.7e-06	0.36	Pancreas	FALSE
rs557211	0.0321	1.019e-13	CHOL	intron_variant	PCSK9	2.7e-06	0.36	Pancreas	FALSE
rs557435	0.0327	5.436e-28	LDL	intron_variant	PCSK9	6.1e-05	0.28	Spleen	FALSE
rs55862049	0.0461	1,00E-300	LDL	intergenic_variant	PCSK9	3.7e-15	0.58	Adipose - Visceral (Omentum)	FALSE
rs565436	0.0324	2.169e-39	LDL	intron_variant	PCSK9	7,00E-06	0.12	Whole Blood	FALSE
rs568052	0.0179	3.11e-14	LDL	intron_variant	PCSK9	1.3e-05	0.12	Whole Blood	FALSE
rs58506955	-0.0236	9.882e-21	LDL	intergenic_variant	PCSK9	1.1e-05	-0.12	Whole Blood	FALSE
rs58506955	-0.0236	9.882e-21	LDL	intergenic_variant	PCSK9	4.6e-27	-0.6	Adipose - Visceral (Omentum)	FALSE
rs613855	0.0186	2.335e-21	LDL	non_coding_transcript_exon_variant	PCSK9	9.7e-06	0.24	Spleen	TRUE
rs613855	0.0307	3.24e-19	CHOL	non_coding_transcript_exon_variant	PCSK9	9.7e-06	0.24	Spleen	TRUE
rs615563	0.0171	9.54e-13	LDL	intron_variant	PCSK9	1.5e-05	0.12	Whole Blood	FALSE
rs624612	0.0251	2.186e-36	LDL	non_coding_transcript_exon_variant	PCSK9	3.6e-05	0.23	Spleen	TRUE
rs624612	0.0251	2.186e-36	LDL	non_coding_transcript_exon_variant	PCSK9	1.4e-06	0.13	Whole Blood	TRUE
rs624612	0.035	2.042e-23	CHOL	non_coding_transcript_exon_variant	PCSK9	3.6e-05	0.23	Spleen	TRUE
rs624612	0.035	2.042e-23	CHOL	non_coding_transcript_exon_variant	PCSK9	1.4e-06	0.13	Whole Blood	TRUE
rs625619	0.0288	1.224e-16	CHOL	intron_variant	PCSK9	0.00011	0.097	Whole Blood	FALSE
rs625619	0.0334	1.374e-50	LDL	intron_variant	PCSK9	0.00011	0.097	Whole Blood	FALSE
rs630431	0.0185	1.042e-13	LDL	intron_variant	PCSK9	1.1e-05	0.12	Whole Blood	FALSE
rs634272	0.0313	1.243e-14	CHOL	intron_variant	PCSK9	0.00011	0.11	Whole Blood	FALSE
rs634272	0.0442	3.33e-276	LDL	intron_variant	PCSK9	0.00011	0.11	Whole Blood	FALSE
rs639750	0.0334	2.278e-19	CHOL	intron_variant	PCSK9	1.1e-08	0.15	Whole Blood	FALSE
rs639750	0.0475	1.17e-286	LDL	intron_variant	PCSK9	1.1e-08	0.15	Whole Blood	FALSE
rs667353	0.0236	4.063e-11	CHOL	intron_variant	PCSK9	1.3e-08	0.15	Whole Blood	FALSE
rs667353	0.0272	1.167e-36	LDL	intron_variant	PCSK9	1.3e-08	0.15	Whole Blood	FALSE
rs6681159	0.038	6.09e-264	LDL	intron_variant	PCSK9	5.8e-05	-0.25	Adipose - Visceral (Omentum)	FALSE
rs676297	-0.0376	3.052e-43	LDL	intron_variant	PCSK9	6.2e-07	0.37	Adipose - Visceral (Omentum)	FALSE
rs693668	0.0511	1.63e-302	LDL	intron_variant	PCSK9	8.8e-14	0.19	Whole Blood	TRUE
rs693668	0.0511	1.63e-302	LDL	intron_variant	PCSK9	7.7e-06	0.33	Liver	TRUE
rs693668	0.0511	1.63e-302	LDL	intron_variant	PCSK9	1.3e-05	0.23	Spleen	TRUE
rs693668	0.0333	1.933e-20	CHOL	intron_variant	PCSK9	8.8e-14	0.19	Whole Blood	TRUE
rs693668	0.0333	1.933e-20	CHOL	intron_variant	PCSK9	7.7e-06	0.33	Liver	TRUE
rs693668	0.0333	1.933e-20	CHOL	intron_variant	PCSK9	1.3e-05	0.23	Spleen	TRUE
rs72660536	0.0416	2.238e-40	LDL	intergenic_variant	PCSK9	5.3e-15	0.57	Adipose - Visceral (Omentum)	FALSE
rs72660539	0.0466	4.56e-283	LDL	intergenic_variant	PCSK9	1.8e-14	0.58	Adipose - Visceral (Omentum)	FALSE
rs7546522	-0.0264	4.916e-20	LDL	non_coding_transcript_exon_variant	PCSK9	2.1e-05	-0.15	Whole Blood	FALSE
rs7546522	-0.04	2.728e-16	CHOL	non_coding_transcript_exon_variant	PCSK9	2.1e-05	-0.15	Whole Blood	FALSE
rs7552841	0.0253	1.491e-24	LDL	intron_variant	PCSK9	3.4e-06	0.12	Whole Blood	FALSE
rs9436961	-0.0388	8.8e-283	LDL	intergenic_variant	PCSK9	1.6e-25	-0.59	Adipose - Visceral (Omentum)	FALSE
rs9436961	-0.0388	8.8e-283	LDL	intergenic_variant	PCSK9	1.6e-06	-0.13	Whole Blood	FALSE
rs9436961	-0.0277	6.773e-14	CHOL	intergenic_variant	PCSK9	1.6e-25	-0.59	Adipose - Visceral (Omentum)	FALSE
rs9436961	-0.0277	6.773e-14	CHOL	intergenic_variant	PCSK9	1.6e-06	-0.13	Whole Blood	FALSE
rs9700736	-0.0259	6.096e-09	CHOL	intergenic_variant	PCSK9	2,00E-06	-0.37	Adipose - Visceral (Omentum)	FALSE
rs9700736	-0.017	6.214e-09	LDL	intergenic_variant	PCSK9	2,00E-06	-0.37	Adipose - Visceral (Omentum)	FALSE

Supplementary Table IV: All CVD associated GWAS hits in non-coding regions of the respective PCSK (+/-50KB) that are identified as eQTLs regulating the expression of that PCSK in different tissues (eQTL-expression quantitative trait locus).

Supplementary Table V:

#0:PFC	1:LLR_GRG	2:Gene1	3:Gene2	4:Tissue
0.953	NA	HUWE1	CTSD	All Tissues
0.983	NA	PPT1	AP1B1	All Tissues
1.000	NA	ARFGEF1	ARFGEF2	All Tissues
0.985	NA	AP2M1	CTSD	All Tissues
0.877	NA	SERPINB8	FURIN	All Tissues
0.952	NA	ATP6V0A1	ARFGEF1	All Tissues
0.907	NA	HUWE1	ATP6V0A1	All Tissues
0.851	NA	CLGN	PCSK9	All Tissues
0.908	NA	CTSD	ARHGAP1	All Tissues
0.930	NA	PCSK7	ARFGEF2	All Tissues
0.860	NA	SCG5	PCSK2	All Tissues
0.992	NA	FURIN	PXYLP1	All Tissues
0.953	NA	PCSK7	XPNPEP1	All Tissues
0.975	NA	HUWE1	FURIN	All Tissues
0.952	NA	AP2M1	ANXA2	All Tissues
0.980	NA	ERGIC3	NUCB1	All Tissues
0.807	NA	NUCB1	PYGL	All Tissues
0.918	NA	FURIN	MDP1	All Tissues
0.951	NA	PTPRN	PCSK2	All Tissues
0.964	NA	MBTPS1	HDAC1	All Tissues
0.962	NA	SLC25A1	PYGL	All Tissues
0.999	NA	AP2M1	FURIN	All Tissues
0.890	NA	AP1B1	XPNPEP1	All Tissues
0.811	NA	PPT1	ANXA2	All Tissues
1.000	NA	CTSD	PPT1	All Tissues
0.998	NA	AP1B1	ARFGEF2	All Tissues
0.993	NA	CTSD	AP1B1	All Tissues
0.968	NA	FURIN	PPT1	All Tissues
0.843	NA	HUWE1	AP1B1	All Tissues
0.921	NA	MBTPS1	PEX19	All Tissues
0.974	NA	CLTB	AP1B1	All Tissues
0.895	NA	AP2M1	ATP6V0A1	All Tissues
0.942	NA	ERGIC3	AP1B1	All Tissues
0.998	NA	FURIN	MMP14	All Tissues
0.998	NA	CTSD	XPNPEP1	All Tissues
0.816	NA	AP2M1	PPT1	All Tissues
0.976	NA	PCSK7	ARFGEF1	All Tissues
0.960	NA	CPE	PCSK2	All Tissues
0.884	NA	CLTB	NUCB1	All Tissues
0.945	NA	ATP6V0A1	NUCB1	All Tissues
1.000	NA	AP2M1	AP1B1	All Tissues
0.880	NA	FURIN	ATP6V0A1	All Tissues
0.888	NA	MBTPS1	NUCB1	All Tissues
0.914	NA	ERGIC3	FURIN	All Tissues
1.000	NA	RHOT1	PEX19	All Tissues
0.958	NA	CTSD	ANXA2	All Tissues
0.988	NA	RHOT1	SLC25A1	All Tissues
0.998	NA	ATP6V0A1	AP1B1	All Tissues
0.872	NA	AP1B1	ARHGAP1	All Tissues
0.879	NA	FURIN	CLTB	All Tissues
0.985	NA	MBTPS1	GOSR1	All Tissues
0.999	NA	AP1B1	ARFGEF1	All Tissues
0.928	NA	CTSD	NUCB1	All Tissues
0.976	NA	HUWE1	ARFGEF1	All Tissues
0.994	NA	ATP6V0A1	ARFGEF2	All Tissues
0.925	NA	FURIN	CTSD	All Tissues
0.998	NA	CTSD	PYGL	All Tissues
0.927	NA	HUWE1	PYGL	All Tissues
0.943	NA	SLC25A1	AP1B1	All Tissues
0.804	NA	ATP6V0A1	PYGL	All Tissues
0.909	NA	SCG5	CPE	All Tissues
0.968	NA	AP1B1	NUCB1	All Tissues
0.876	NA	RHOT1	PCSK9	All Tissues
0.997	NA	ANXA2	PCSK9	All Tissues
0.875	NA	FURIN	ARHGAP1	All Tissues
0.948	NA	HUWE1	ARFGEF2	All Tissues
0.964	NA	CTSD	ATP6V0A1	All Tissues
0.844	NA	CLTB	ARFGEF2	All Tissues
0.942	NA	FURIN	DDX56	All Tissues
0.921	NA	SLC25A1	PCSK9	All Tissues
0.847	NA	PPT1	ATP6V0A1	All Tissues
0.958	NA	AP2M1	NUCB1	All Tissues
0.920	NA	PYGL	ANXA2	All Tissues
0.863	NA	FURIN	PYGL	All Tissues
0.804	NA	PPT1	HDAC1	All Tissues
0.839	NA	CTSD	SLC25A1	All Tissues
0.866	NA	ATP6V0A1	ARHGAP1	All Tissues
0.874	NA	FURIN	AP1B1	All Tissues
0.843	NA	ATP6V0A1	XPNPEP1	All Tissues
0.861	NA	PPT1	ARHGAP1	All Tissues
0.812	NA	NUCB1	ARHGAP1	All Tissues
1.000	NA	LDLR	PCSK9	All Tissues
1.000	NA	AP2M1	CLTB	All Tissues
-	NA	FURIN	XPNPEP1	All Tissues
-	NA	PPT1	SLC25A1	All Tissues
-	NA	AP2M1	DDX56	All Tissues
-	NA	PPT1	PCSK5	All Tissues
-	NA	MBTPS1	PCSK5	All Tissues
-	NA	MBTPS1	PPT1	All Tissues

Supplementary Table V: Protein-protein interaction network data.

Supplementary Table VI:

Protein	Number of patents	Disease	CVD connection	Classification of patents
PCSK1	5	T2D, Polycystic kidney disease, Apoe diseases (Alzheimer)	y	SNP, screening
PCSK2	7	Pancreatic neuroendocrine tumors, T1D, Malignant thyroid, Apoe diseases (Alzheimer)	y	Biomarker
PCSK3	36	HIV-1, lung adenocarcinoma, cancer	y	Biomarker, medication
PCSK4	1	NA		Medication
PCSK5	1	Colon cancer		Biomarker
PCSK6	13	Renal, Breast cancer, Dysplasia	y	Medication, biomarker
PCSK7	3	Rheumatoid arthritis		SNP, biomarker
PCSK8	3	Skeletal disease, MBTPS1 related disease		Medication
PCSK9	1097	LDL	y	Medication, biomarker
**Sum**	**1166**			

Supplementary Table VI: Overview of the patents found in relation to the PCSK family in PatentScope public database, as of Dec 2021.

2 References

Abifadel, M., Varret, M., Rabes, J.P., Allard, D., Ouguerram, K., Devillers, M., et al. (2003). Mutations in PCSK9 cause autosomal dominant hypercholesterolemia. *Nat Genet* 34(2)**,** 154-156. doi: 10.1038/ng1161.

Chen, S., Cao, P., Dong, N., Peng, J., Zhang, C., Wang, H., et al. (2015). PCSK6-mediated corin activation is essential for normal blood pressure. *Nat Med* 21(9)**,** 1048-1053. doi: 10.1038/nm.3920.

Fujimaki, T., Kato, K., Yokoi, K., Oguri, M., Yoshida, T., Watanabe, S., et al. (2010). Association of genetic variants in SEMA3F, CLEC16A, LAMA3, and PCSK2 with myocardial infarction in Japanese individuals. *Atherosclerosis* 210(2)**,** 468-473. doi: 10.1016/j.atherosclerosis.2009.11.050.

Ghosh, S., Vivar, J., Nelson, C.P., Willenborg, C., Segre, A.V., Makinen, V.P., et al. (2015). Systems Genetics Analysis of Genome-Wide Association Study Reveals Novel Associations Between Key Biological Processes and Coronary Artery Disease. *Arterioscler Thromb Vasc Biol* 35(7)**,** 1712-1722. doi: 10.1161/atvbaha.115.305513.

Hoogeveen, R.C., Gaubatz, J.W., Sun, W., Dodge, R.C., Crosby, J.R., Jiang, J., et al. (2014). Small Dense Low-Density Lipoprotein-Cholesterol Concentrations Predict Risk for Coronary Heart DiseaseSignificance. doi: 10.1161/ATVBAHA.114.303284.

Jackson, R.S., Creemers, J.W., Ohagi, S., Raffin-Sanson, M.L., Sanders, L., Montague, C.T., et al. (1997). Obesity and impaired prohormone processing associated with mutations in the human prohormone convertase 1 gene. *Nat Genet* 16(3)**,** 303-306. doi: 10.1038/ng0797-303.

Jin, W., Wang, X., Millar, J.S., Quertermous, T., Rothblat, G.H., Glick, J.M., et al. (2007). Hepatic proprotein convertases modulate HDL metabolism. *Cell Metab* 6(2)**,** 129-136. doi: 10.1016/j.cmet.2007.07.009.

Kuhn, T.C., Knobel, J., Burkert-Rettenmaier, S., Li, X., Meyer, I.S., Jungmann, A., et al. (2020). Secretome Analysis of Cardiomyocytes Identifies PCSK6 (Proprotein Convertase Subtilisin/Kexin Type 6) as a Novel Player in Cardiac Remodeling After Myocardial Infarction. *Circulation* 141(20)**,** 1628-1644. doi: 10.1161/Circulationaha.119.044914.

Leak, T.S., Keene, K.L., Langefeld, C.D., Gallagher, C.J., Mychaleckyj, J.C., Freedman, B.I., et al. (2007). Association of the proprotein convertase subtilisin/kexin-type 2 (PCSK2) gene with type 2 diabetes in an African American population. *Mol Genet Metab* 92(1-2)**,** 145-150. doi: 10.1016/j.ymgme.2007.05.014.

Li, N.F., Luo, W.L., Juhong, Z., Yang, J., Wang, H.M., Zhou, L., et al. (2010). Associations between genetic variations in the FURIN gene and hypertension. *Bmc Medical Genetics* 11. doi: Artn 124 10.1186/1471-2350-11-124.

O'Rahilly, S., Gray, H., Humphreys, P.J., Krook, A., Polonsky, K.S., White, A., et al. (1995). Brief report: impaired processing of prohormones associated with abnormalities of glucose homeostasis and adrenal function. *N Engl J Med* 333(21)**,** 1386-1390. doi: 10.1056/NEJM199511233332104.

Perisic, L., Hedin, E., Razuvaev, A., Lengquist, M., Osterholm, C., Folkersen, L., et al. (2013). Profiling of atherosclerotic lesions by gene and tissue microarrays reveals PCSK6 as a novel protease in unstable carotid atherosclerosis. *Arterioscler Thromb Vasc Biol* 33(10)**,** 2432-2443. doi: 10.1161/atvbaha.113.301743.

Rykaczewska, U., Suur, B.E., Rohl, S., Razuvaev, A., Lengquist, M., Sabater-Lleal, M., et al. (2020). PCSK6 Is a Key Protease in the Control of Smooth Muscle Cell Function in Vascular Remodeling. *Circ Res* 126(5)**,** 571-585. doi: 10.1161/CIRCRESAHA.119.316063.

Sakai, J., Nohturfft, A., Goldstein, J.L., and Brown, M.S. (1998). Cleavage of sterol regulatory element-binding proteins (SREBPs) at site-1 requires interaction with SREBP cleavage-activating protein - Evidence from in vivo competition studies. *Journal of Biological Chemistry* 273(10)**,** 5785-5793. doi: DOI 10.1074/jbc.273.10.5785.

Stawowy, P., Kallisch, H., Borges Pereira Stawowy, N., Stibenz, D., Veinot, J.P., Grafe, M., et al. (2005). Immunohistochemical localization of subtilisin/kexin-like proprotein convertases in human atherosclerosis. *Virchows Arch* 446(4)**,** 351-359. doi: 10.1007/s00428-004-1198-7.

Stawowy, P., Kallisch, H., Stawowy, N.B.P., Stiebenz, D., Veinot, J.P., Chretien, M., et al. (2004). Furin and PC5 are localized in macrophages/foam cells in human atherosclerotic plaques and are required for integrin alpha v and MT1-MMP activation. *Circulation* 110(17)**,** 247-247.
Testa, G., Staurenghi, E., Giannelli, S., Sottero, B., Gargiulo, S., Poli, G., et al. (2021). Up-regulation of PCSK6 by lipid oxidation products: A possible role in atherosclerosis. *Biochimie* 181**,** 191-203. doi: 10.1016/j.biochi.2020.12.012.
